# Synthesis and Biological Evaluation of New Glycoconjugated LDH Inhibitors as Anticancer Agents †

**DOI:** 10.3390/molecules24193520

**Published:** 2019-09-28

**Authors:** Felicia D’Andrea, Giulia Vagelli, Carlotta Granchi, Lorenzo Guazzelli, Tiziano Tuccinardi, Giulio Poli, Dalila Iacopini, Filippo Minutolo, Valeria Di Bussolo

**Affiliations:** 1 Dipartimento di Farmacia, Università di Pisa, Via Bonanno 33, 56126 Pisa, Italy; carlotta.granchi@unipi.it (C.G.); lorenzo.guazzelli@unipi.it (L.G.); tiziano.tuccinardi@unipi.it (T.T.); giulio.poli@unipi.it (G.P.); filippo.minutolo@unipi.it (F.M.); 2 Dipartimento di Chimica e Chimica Industriale, Università di Pisa, Via G. Moruzzi 3, 56124 Pisa, Italy; vagelligiulia@gmail.com (G.V.); dalila.iacopini@phd.unipi.it (D.I.)

**Keywords:** LDH inhibitors, glycoconjugates, anticancer agents

## Abstract

Conjugation of known biologically active molecules to carbohydrate frameworks represents a valuable option for the preparation of hybrid, structurally-related families of compounds with the aim of modulating their biological response. Therefore, we present here a study on the preparation of d-*galacto*, d-*manno*, d-*gluco*, and d-*lactose* glycoconjugates of an established *N*-hydroxyindole-based (NHI) inhibitor of lactated dehydrogenase (LDH). Structural variations involved the sugar stereochemistry and size as well as the anchoring point of the NHI on the carbohydrate frame (either C-1 or C-6). In the case of the galactose anomeric glycoconjugate (C-1), intriguing solvent-dependent effects were observed in the glycosylation stereochemical outcome. The biological activity of the deprotected glycoconjugates in contrasting lactate formation and cancer cell proliferation are described.

## 1. Introduction

Deregulation of normal cell metabolism is one of the most important hallmark of cancer cells. In fact, metabolic activities are often reprogrammed in neoplastic masses relative to healthy tissues [1]. The Warburg effect, consisting of an augmented aerobic glycolysis, represents a typical case of an altered metabolic pathway in cancer [2]. This effect is characterized by an increased glucose uptake and lactate production in cancer cells, regardless of hypoxic/normoxic conditions surrounding the cells. Such an increase in glycolytic flux produces energy and glycolytic intermediates that are needed by rapidly proliferating cells.

Numerous research lines have so far focused on the development of new therapeutic agents that can potentially take advantage of this characteristic metabolic profile present in cancer cells by acting as anti-glycolytic agents [3,4]. Furthermore, the consequential overexpression of glucose transporters (GLUTs) in the membrane of cancer cells can be either targeted by modulators of the carbohydrate flux through these proteins [5] or exploited for the preferential intracellular accumulation of glycoconjugates containing cytotoxic drugs [6].

One of the key enzymes in glycolysis is lactate dehydrogenase (LDH), which catalyzes the reversible transformation of pyruvate into lactate. This enzyme is constituted by four monomeric subunits (LDH-A and LDH-B) and may, therefore, be found in humans as five different isoforms (hLDH1-5). Cancer cells are often found to overexpress subunit LDH-A and, thus, its tetrameric form hLDH5, therefore may represent a promising therapeutic target [7]. In fact, numerous research efforts have been so far committed to the discovery of novel potent inhibitors of hLDH5 as potential anticancer drugs, even though very often these inhibitors display poor cell membrane permeability due to the high polar nature of their structures [8].

**Figure 1:**
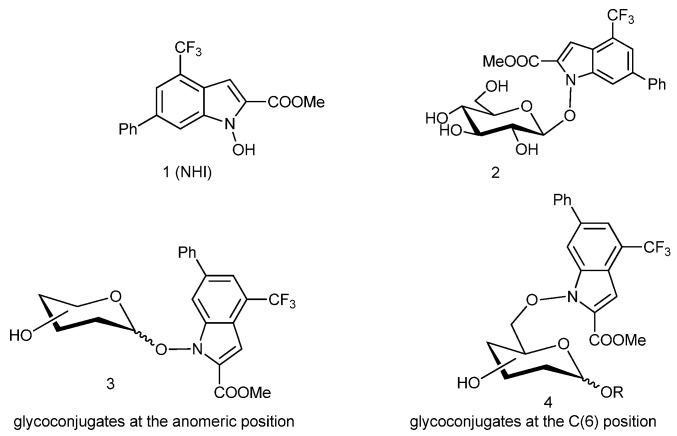
Structure of NHI (**1**) and its known (**2**) and novel (**3**, **4**) glycoconjugates.

We have previously developed a class of *N*-hydroxyindole-based compounds (NHIs), such as **1** (Figure 1), which are able to inhibit *h*LDH5. Specifically, these compounds inhibit *h*LDH5 with *K*_i_ values in the micromolar range in enzymatic assays. Considering that the high rate of glycolysis in cancer cells is associated with a striking glucose avidity, we set out to exploit this feature in order to improve the tumor-specificity of this approach and minimize off-target effects. We recently reported that glucoconjugated NHIs, in particular **2** (Figure 1), remarkably reduced the cellular production of lactic acid in cancer cells and blocked the growth of tumor cells [9,10].

In order to get a further insight into the potential role played by the carbohydrate structure on the inhibitor activity and on the cellular uptake through facilitative glucose transporters—namely its size, stereochemistry, and anchoring point, herein we describe a structural modulation of this class of glycoconjugate inhibitors, exemplified by **3** and **4** (Figure 1), with a *N*-hydroxyindole moiety linked to the anomeric or the C(6) position of monosaccharides of the d-*galacto*, d-*manno*, d-*gluco* series and of d-*lactose*.

## 2. Results and Discussion

### 2.1. Preparation of Glycoconjugates

**Scheme 1:**
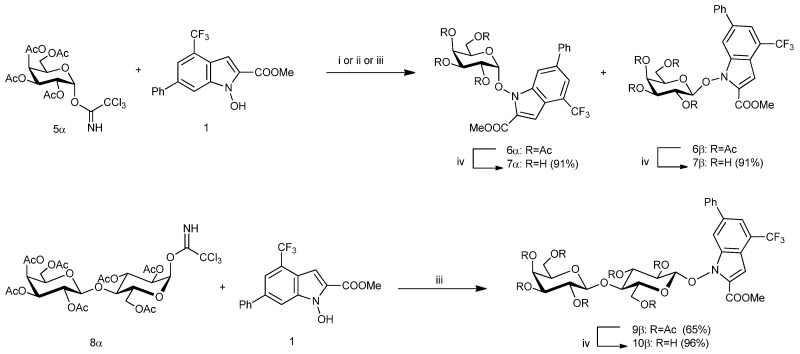
Preparation of glycosyl derivatives **6**, **7** and **9**, **10**. Reagents and conditions: (**i**) TMSOTf, CH_2_Cl_2_, molecular sieves AW 300, 0 °C (30 min) and RT (3.5 h), (**6α**: 29%, **6β**: 42%); (**ii**) TMSOTf, CH_3_CN, molecular sieves AW 300, 0 °C (30 min) and RT (2.5 h), (**6α**: 54%, **6β**: 13%); (**iii**) BF_3_·Et_2_O, CH_2_Cl_2_, molecular sieves AW 300, −10 °C to RT (2 h), (**6β**: 78%); (**iv**) MeONa-MeOH 0.33M, 2:3 CH_2_Cl_2_-MeOH, 0 °C to RT.

In our general approach to the synthesis of galactose glycosides **6α**, **6β**, and lactose glycoside **8β**, known glycosyl donors **5α** [11] and **8α** [12,13] were reacted under several conditions with the NHI **1 [14]** fragment, which was used as the glycosyl acceptor (Scheme 1 and Table 1). The glycosylation reactions have been carried out with trichloroacetimidate glycosyl donors in the presence of the two most widely used promoters (TMSOTf and BF_3_·Et_2_O) [15,16] and proceeded with good yields. Of a certain interest was observing how the nature of the promoter and the solvent both affected the α/β ratio in the formation of compound **6** (Table 1). In particular, when donors **5α** and **8α** were coupled with acceptors **1** using BF_3_·Et_2_O (1.3 eq) in dry CH_2_Cl_2_, a high selectivity for the beta anomer was obtained (**6α**:**6β** 5:95, **9α**:**9β** < 1:99).

Moreover, when the glycosylation of NHI **1** using **5α** (Scheme 1 and Table 1) was carried out with TMSOTf (0.2 eq) as the promoter in dry CH_2_Cl_2_ the reaction proceeded, surprisingly, with poor stereoselectivity (**6α**:**6β** 35:65). The use of dry MeCN as the solvent gave a more satisfactory, albeit reversed, stereoselectivity (**6α**:**6β** 78:22). Although the known “nitrile effect” [17] could be invoked when MeCN is used as the solvent, the results obtained confirm how reaction conditions and donor/acceptor structures can affect the stereochemical outcome of a glycosylation reaction. Indeed, in both **5α** and **8α** substrates, it can be speculated that the participation of the neighboring 2-*O*-acyl substituent can lead to the formation of an acyloxonium intermediate. Consequently, in the presence of BF_3_·Et_2_O, the C-O bond on the anomeric carbon of the acyloxonium intermediate is only weakened, to afford the formation of 1,2-*trans* linkage with a very high selectivity and a S_N_2-like displacement from the glycosyl acceptor NHI **1** affords **6β** and **9β**. Conversely, in the presence of a stronger promoter such as TMSOTf in CH_2_Cl_2_, the acyloxonium mainly evolves towards the classic oxacarbenium ion to afford **6α** and **6β** in 35/65 ratio.

In all cases, glycosides **6α**, **6β**, and **9β** were efficiently separated by chromatographic means and fully characterized. NMR data confirmed the glycoside structures and, in particular, the high values (8.1 and 7.8 Hz) of the *J*_1,2_ coupling constants, which is in agreement with an axial–axial disposition of H-1 and H-2, confirmed the beta configuration of the glycosidic bonds in **6β** and **9β**. In the case of **6α** the equatorial–axial disposition of H-1 and H-2 was confirmed by the low value (4.0 Hz) of the *J*_1,2_ coupling constant.

The deacetylation of **6α**, **6β**, and **9β** (Scheme 1) by treatment with MeONa in CH_2_Cl_2_-MeOH afforded deprotected glycoconjugates **7α**, **7β**, and **10β** in very good yields (91–96%).

**Scheme 2:**
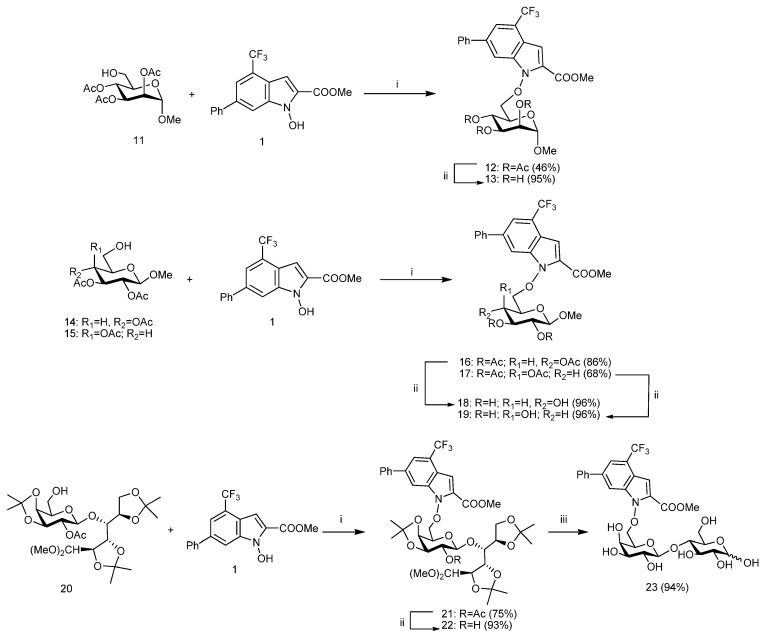
Preparation of glycosyl derivatives **12**, **13**, **16**–**19**, and **21**–**23**. Reagents and conditions: (**i**) PPh_3_, DIAD or DMEAD, dry THF, 0 °C to RT (3 h); (**ii**) MeONa-MeOH 0.33 M, 2:3 CH_2_Cl_2_-MeOH, 0 °C to RT; (**iii**) 80% aq AcOH, 80 °C, 4 h.

The synthesis of the protected glycoconjugates **12**, **16**, and **17** is described in Scheme 2. A Mitsunobu reaction [18] was performed between the *N*-hydroxyindole derivative **1** and known carbohydrate building blocks **11** [19], **14** [20], and **15** [21] in the presence of diisopropyl azodicarboxylate (DIAD) or di-2-methoxyethyl azodicarboxylate (DMEAD) and triphenylphosphine (PPh_3_) affording glycoconjugates **12** (46% yield), **16** (86% yield), and **17** (68% yield) respectively.

Poliacetonide lactose derivatives have been widely used as suitable protected forms of the natural disaccharide. Indeed, only one or two unprotected hydroxyl groups are left on the *galacto* non-reducing moiety, which allows for consecutive feasible modifications [22,23,24,25]. Herein, known poliacetonide derivatives **20** [26] was coupled with NHI **1** again through a Mitsunobu condensation with PPh_3_, DMEAD in dry THF giving disaccharide derivative **21** in a good yield (75%, Scheme 2).

The deacetylation of **12**, **16**, **18**, and **21** (Scheme 2) via base-catalyzed transesterification with MeONa in CH_2_Cl_2_-MeOH afforded deprotected glycoconjugates **13**, **17**, **19**, and **22** in very good yields (93–96%). Finally, the acetals protecting groups were cleaved from **22** by acid hydrolysis (80% aq AcOH) to afford disaccharide derivative **23** (94% yield) mostly as the α-pyranose anomer (>95%, NMR, DMSO-d_6_).

All compounds were characterized and their mono- and two-dimensional NMR analyses (^1^H, ^13^C, DEPT-135, COSY, and HSQC) were consistent with their structures (see experimental section).

### 2.2. Enzymatic Assay

The inhibitory activities of the newly synthesized sugar conjugates were measured as IC_50_ values on purified human enzyme isoform *h*LDH5 (Table 2). Galloflavin was used as reference compound, because it is a well-known phenolic LDH inhibitor which is often used in enzymatic LDH assays [27,28], since it is easily synthesizable and it is also commercially available. Among the newly synthesized sugar conjugates, compounds **7α**, **7β**, and **10β**, which are the glyco-conjugates bearing the *N*-hydroxyindole moiety at the anomeric position, showed a moderate inhibition potency towards *h*LDH5, with IC_50_ values ranging from 229 to 246 μM, compared to the IC_50_ values of the parent gluco-conjugate **2** (IC_50_ = 112.8 ± 5.6 µM) and of its aglycone moiety **1** (IC_50_ = 10.8 ± 3.5 µM) Conversely, compounds **13**, **18**, **19**, and **23**, in which the *N*-hydroxyindole portion is linked to the C(6) position of mono- or disaccharide units, were inactive at the maximum tested concentration of 500 μM.

### 2.3. Molecular Modeling Studies

**Figure 2:**
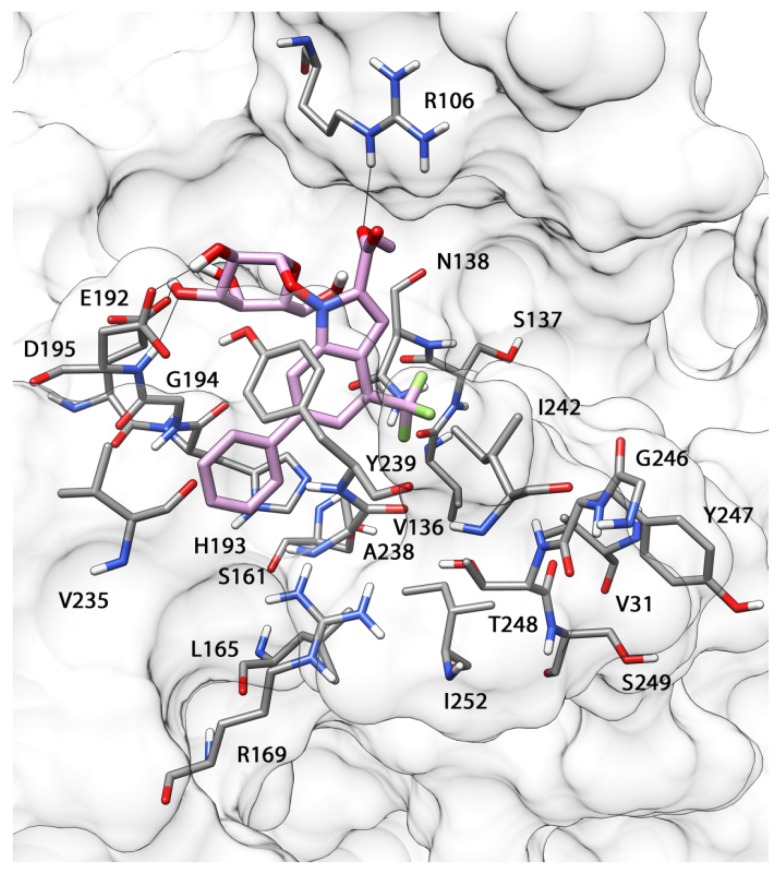
Binding disposition of **7α** into *h*LDH5.

Docking studies were carried out in order to explore the binding disposition of these ligands into the *h*LDH5 binding site. The seven compounds were thus docked into the minimized average structure of *h*LDH5 previously obtained [14] by applying a robust Autodock procedure that had shown good results in virtual screening studies and in the prediction of ligands’ binding poses [30,31]. Figure 2 illustrates the predicted binding disposition of **7α**. The methyl ester group shows an H-bond with R106, the phenyl ring is inserted into a lipophilic cavity mainly delimited by H193, V235, and A238, whereas the trifluoromethyl substituent shows an H-bond interaction with N138. With regards to the glycosidic portion of the molecule, it shows H-bonds to the carboxylic portion of E192 and D195 and also with the backbone nitrogen of this latter residue.

As shown in Figure S1 compound **7β** shows a binding orientation very similar to that obtained for **7α** with the same interactions, except for the loss of the H-bond with the carboxylic group of D195. According to our model, the substitution of the monosaccharide with a disaccharide unit does not seem to determine important modifications of the binding orientation of the resulting compound. As shown in Figure S2, the docking analysis of **10β** highlights a slight shift of the phenyl-indole fragment of the ligand and the loss of the interaction of its trifluoromethyl substituent with N138. However, the H-bond of the ligand methyl ester group with R106 and the lipophilic interactions of the phenyl ring are maintained. With regards to the disaccharide fragment, it shows H-bonds with R106, E192, and D195. The addition of the *N*-hydroxyindole portion to the C(6) position of mono- or disaccharide unit determines a general inactivity. This behavior, according to our model, is likely due to the different geometry of these ligands and, in particular, their impossibility to adopt the binding orientation highlighted for the anomeric derivatives; as shown in Figure S3, compounds **13**–**23** show a very similar binding disposition that is completely different from that shown by compounds **7α**, **7β**, and **10β**, thus supporting their inactivity. The phenyl ring shows lipophilic interactions with I252, the methyl ester group and the trifluoromethyl substituent do not show any interaction and result to be water-exposed, whereas the glycosidic portions show H-bonds with R169, H193 and the backbone of T248.

### 2.4. Cancer Cell Antiproliferative Potency Assays

The cytotoxicity of glycoconjugates on HeLa and A549 cancer cells was assessed and compared to that of parent glucose derivative **2**. Cells were incubated for 72 h with different concentrations of the compounds, then cell death was quantified by the Sulforhodamine B (SRB) assay [10]. The antiproliferative activities are expressed as IC_50_ values (Table 3). Glycoconjugates **7α** and **7β** showed good inhibition potencies in A549 cells, with IC_50_ values comparable to that of reference compound **2**. Moreover, they were more effective in reducing cancer cell growth in HeLa cells (about two-fold for **7β** or three-fold for **7α**) than in A549 cells. Lactose-conjugated **10β** was less potent in both cell lines: it was inactive at 100 μM in A549 cells, and it reached an IC_50_ value of 81.5 μM in HeLa cells.

### 2.5. Cellular Lactate Production Inhibition Assay

**Figure 3:**
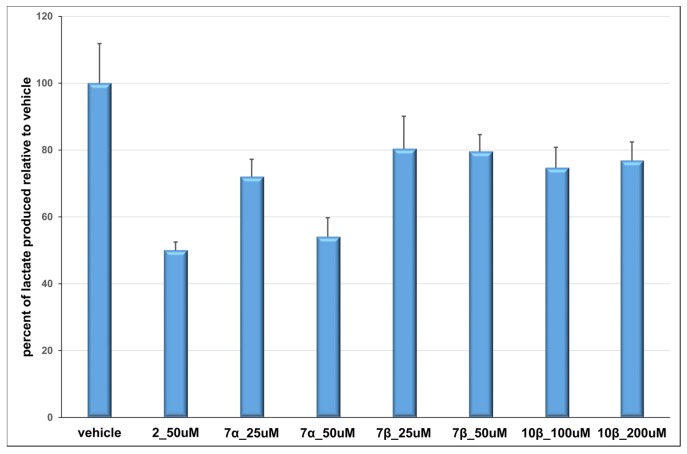
Lactate production inhibition of glycoconjugates in HeLa human cancer cells after 8 h incubation. Averages from three or more independent experiments are depicted, with error bars depicting standard error (*n* = 3).

The ability of glycoconjugates **7α**, **7β**, and **10β** to inhibit lactate production in cancer cells was evaluated using the previously reported technique [10] and the results are displayed in Figure 3. Compounds **7α** and **7β** were tested at two different concentrations, 25 and 50 µM, whereas the less cytotoxic derivative **10β** was assayed at 100 and 200 µM for their ability to reduce lactate production in HeLa cells following an 8 h incubation. The glucose derivative **2** was able to reduce lactate production of about 50% at 50 µM, in agreement with previous results [9,10]. A noticeable dose-dependent reduction in lactate production was found with the **α**-galacto-conjugate **7α**, which displayed a reduction of lactate production greater than 60% at 50 µM.

## 3. Materials and Methods

Melting points were determined with a Kofler hot-stage apparatus and are uncorrected. Optical rotations were measured with an ATAGO AP-300 Automatic Polarimeter at 25±2 °C. ^1^H NMR spectra were recorded in appropriate solvents with a Bruker Avance II operating at 250.13 MHz. ^13^C NMR spectra were recorded with the spectrometers operating at 62.9 MHz. The assignments were made, when possible, with the aid of DEPT, COSY, HSQC experiments. The first order proton chemical shifts *δ* are referenced to either residual CDCl_3_ (*δ*_H_ 7.26, *δ*_C_ 77.0), residual CD_3_CN (*δ*_H_ 1.94, *δ*_C_ 1.28), residual CD_3_OD (*δ*_H_ 3.31, *δ*_C_ 49.0) or residual DMSO-*d6* (*δ*_H_ 2.49, *δ*_C_ 39.5) and *J*-values are given in Hz. Spin multiplicity was indicated by s = singlet, d = doublet, t = triplet, bt = broad triplet, q = quartet, m = multiplet. Coupling constants *J* were reported in Hertz (Hz). All reactions were followed by TLC on Kieselgel 60 F_254_ with detection by UV light and/or with ethanolic 10% phosphomolybdic or sulfuric acid, and heating. Kieselgel 60 (Merck, S.p.A., Milano, Italy, 70–230 and 230–400 mesh, respectively) was used for column and flash chromatography. Some of flash chromatography were conducted by the automated system Isolera Four SV^TM^ (Biotage^®^, Uppsala, Sweden), equipped with UV detector with variable wavelength (200–400 nm). Solvents were dried by distillation according to standard procedures, and storage over 4Å molecular sieves activated for at least 24 h at 200 °C. All reagents were purchased from Aldrich Chemical Co. and were used without further purification. Molecular sieves (4Å and AW-300) and PPh_3_ were activated (2–8 h at 150 °C.) before use. All reactions involving air- or moisture-sensitive reagents were performed under an argon atmosphere using anhydrous solvents. Anhydrous dimethylformamide (DMF), dichloromethane (CH_2_Cl_2_), 1,2-dichloethane (DCE), and THF were purchased from Sigma-Aldrich. Other dried solvents were obtained by distillation according to standard procedure and stored over 4Å molecular sieves activated. MgSO_4_ or Na_2_SO_4_ were used as the drying agents for solutions. 1-Hydroxy-6-phenyl-4-(trifluoromethyl)-1H-indole-2-methyl-carboxylate (**1**) [14], 2,3,4,6-tetra-*O*-acetyl-α-d-galactopyranosyl trichloroacetimidate (**5α**) [11], 4-*O*-(2,3,4,6-tetra-*O*-acetyl-β-d-galactopyranosyl)-2,3,6-tri-*O*-acetyl-α-d-glucopyranosyl trichloroacetimidate (**8α**) [12,13], methyl 2,3,4-tri-*O*-acetyl-α-d-mannopyranoside (**11**) [19], methyl 2,3,4-tri-*O*-acetyl-β-d-glucopyranoside (**14**) [20], methyl 2,3,4-tri-*O*-acetyl-β-d-galactopyranoside (**15**) [21], and 4-*O*-(2-*O*-acetyl-3,4-*O*-isopropylidene-β-d-galactopyranosyl)-2,3:5,6-di-*O*-isopropylidene-*aldehydo*-d-glucose dimethyl acetal (**20**) [26] were prepared according to the reported procedure.

### 3.1. General Procedures for Glycosylation

#### 3.1.1. Method A (with TMSOTf in CH_2_Cl_2_ or CH_3_CN)

A solution of the glycosyl donor **5α** [11] (1.30 mmol, 1.3 equiv) and *N*-hydroxyindole derivative **1** [14] (1.00 mmol, 1.0 equiv) in anhyd toluene (10.0 mL) was concentrated under diminished pressure (2 h) for removed any traces of water. The residue was dissolved in anhyd CH_2_Cl_2_ or CH_3_CN (10.0 mL), molecular sieves AW 300 (1.25 g) were added and the resulting mixture was stirred under an argon atmosphere at 0 °C for 30 min. TMSOTf (36 μL, 0.20 mmol, 0.2 equiv) was added and the reaction mixture was stirred for 30 min at 0 °C and then at room temp until TLC analysis showed the complete disappearance of the *N*-hydroxyindole derivative **1**. The reaction mixture was diluted with CH_2_Cl_2_ or CH_3_CN (10.0 mL), neutralized with Et_3_N, filtered through a short pad of Celite^®^ and organic solution was concentrated under diminished pressure. The crude residue was subjected to flash chromatography on silica gel.

#### 3.1.2. Method B (with BF_3_·Et_2_O in CH_2_Cl_2_)

The appropriate glycosyl donor **5α** or **8α** (1.50 mmol, 1.5 equiv) was dried under diminished pressure (1 h). Anhyd CH_2_Cl_2_ (26.0 mL), molecular sieves AW 300 (500 mg), the *N*-hydroxyindole derivative **1** (1.00 mmol, 1.0 equiv) was added and the resulting mixture was stirred under an argon atmosphere at room temp for 1 h. The suspension was cooled to −10 °C, treated with BF_3_·Et_2_O (160 μL, 1.30 mmol, 1.3 equiv) and slowly warmed to room temp. When TLC analysis showed the complete disappearance of the *N*-hydroxyindole derivative **1** the reaction mixture was diluted with CH_2_Cl_2_ (10.0 mL), neutralized with Et_3_N, filtered through a short pad of Celite^®^ and organic solution was concentrated under diminished pressure. The crude residue was subjected to flash chromatography on silica gel.

### 3.2. Methyl 1-(2,3,4,6-Tetra-O-Acetyl-α-d-Galactopyranosyl)-6-Phenyl-4-Trifluoromethyl-1H-Indole-2-Carboxylate (**6α**) and Methyl 1-(2,3,4,6-Tetra-O-Acetyl-β-d-Galactopyranosyl)-6-Phenyl-4-Trifluoromethyl-1H-Indole-2-Carboxylate (**6β**)

The glycosylation of **5α** [11] (100 mg, 0.20 mmol, 1.3 equiv) with *N*-hydroxyindole derivative **1** [14] (52 mg, 0.16 mmol, 1.0 equiv), was performed in dry CH_2_Cl_2_ (1.6 mL) in according to the general procedure (Method A). The reaction was stirred until TLC (4 h, TLC, 7:3 hexane-EtOAc, double elution) showed the complete disappearance of the acceptor **1** (*R*_f_ 0.66) and the formation of two spots at *R*_f_ 0.37 and 0.35. The crude product was constituted (NMR) by a mixture of the anomers **6α** and **6β** in ratio of 35:65, calculated on the basis of the relative H-1 signal intensities. Purification of crude product by flash chromatography on silica gel (7:3 hexane-EtOAc) afforded pure **6α** (31 mg, 29%) and **6β** (44 mg, 42%).

The glycosylation of **5α** [11] (100 mg, 0.20 mmol, 1.3 equiv) with *N*-hydroxyindole derivative **1** [14] (52 mg, 0.16 mmol, 1.0 equiv), was performed in dry CH_3_CN (1.6 mL) in according to the general procedure (Method A). The reaction was stirred until TLC (3 h, TLC, 7:3 hexane-EtOAc, double elution) showed the complete disappearance of the acceptor **1** (*R*_f_ 0.66) and the formation of two spots at *R*_f_ 0.37 and 0.35. The crude product was constituted (NMR) by a mixture of the anomers **6α** and **6β** in ratio of 78:22, calculated on the basis of the relative H-1 signal intensities. Purification of crude product by flash chromatography on silica gel (7:3 hexane-EtOAc) afforded pure **6α** (57.4 mg, 54%) and **6β** (14.4 mg, 13%).

The glycosylation of **5α** [11] (200 mg, 0.40 mmol, 1.5 equiv) with *N*-hydroxyindole derivative **1** [14] (91 mg, 0.28 mmol, 1.0 equiv) was performed in dry CH_2_Cl_2_ (7.0 mL) according to the general procedure (Method B). The reaction was stirred until TLC (2 h, TLC, 7:3 hexane-EtOAc, double elution) showed the complete disappearance of the acceptor **1** (*R*_f_ 0.66) and the formation of two spots at R_f_ 0.37 and 0.35. The crude product was constituted (NMR) by a mixture of the anomers **6α** and **6β** in ratio of 5:95, calculated on the basis of the relative H-1 signal intensities. Purification of crude product by flash chromatography on silica gel (7:3 hexane-EtOAc) afforded pure **6β** (142 mg, 78%) and **6α** (5 mg, 3%).

Compound **6α** is a yellow foam; *R*_f_ 0.37 (7:3 hexane-EtOAc, double elution); mp 64–67 °C (crom); [α]_D_ -10.3 (*c* 0.90 in CHCl_3_); ^1^H NMR (250.13 MHz, CD_3_CN) δ: 7.99 (m, 1H, Ar-*H*), 7.86 (m, 1H, Ar-*H*), 7.80–7.74 (m, 2H, Ar-*H*), 7.55–7.40 (m, 3H, Ar-*H*), 7.30 (qd, 1H, *J* 1.0 Hz, *J* 1.6 Hz, Ar-*H*), 5.91 (d, 1H, *J*_1,2_ 4.0 Hz, H-1), 5.61 (ddd, 1H, *J*_3,4_ 3.0 Hz, *J*_4,5_ 1.4 Hz, *J*_4,6b_ 0.6 Hz, H-4), 5.47 (dd, 1H, *J*_2,3_ 11.2 Hz, H-2), 5.36 (dd, 1H, H-3), 5.02 (m, 1H, H-5), 4.05 (ddd, 1H, *J*_5,6b_ 3.3 Hz, *J*_6a,6b_ 12.1 Hz, H-6b), 4.00 (dd, 1H, *J*_5,6a_ 4.7 Hz, H-6a), 3.91 (s, 3H, COO*Me*), 2.12, 2.11, 1.99, 1.67 (4s, each 3H, 4 × CO*Me*); ^13^C NMR (62.9 MHz, CD_3_CN) δ: 171.2, 171.1, 170.8, 170.7 (4 × *C*OMe), 160.6 (*C*OOMe), 140.4, 139.7, 138.0, 130.4 (4 × Ar-*C*), 130.1, 129.2, 128.5 (Ar-*C*H), 124.8–123.2 (*C*F_3_ + Ar-*C*), 120.4 (Ar-*C*H), 117.2 (Ar-*C*), 113.3, 107.1 (2 × Ar-*C*H), 104.4 (C-1), 70.6 (C-5), 68.9 (C-4), 67.6 (C-2), 66.8 (C-3), 62.5 (C-6), 53.1 (COO*Me*), 21.8, 20.4 (4 × CO*Me*). Anal. Calcd for C_31_H_30_F_3_NO_12_: C, 55.94; H, 4.54%; N, 2.10%; Found: C, 55.98; H, 4.56%; N, 2.14%.

Compound **6β** is a pale yellow solid; *R*_f_ 0.35 (hexane-AcOEt 7:3, double elution); mp 139–142 °C (crom); [α]_D_ + 26.3 (*c* 1.0 in CHCl_3_); ^1^H NMR (250.13 MHz, CD_3_CN) δ: 8.12 (m, 1H, Ar-*H*), 7.84 (m, 1H, Ar-*H*), 7.75 (m, 1H, Ar-*H*), 7.71 (m, 1H, Ar-*H*), 7.54–7.40 (m, 3H, Ar-*H*), 7.23 (qd, 1H, *J* 1.0 Hz, *J* 1.7 Hz, Ar-*H*), 5.58 (d, 1H, *J*_1,2_ 8.1 Hz, H-1), 5.40 (m, 2H, 2-H, H-4), 5.27 (dd, 1H, *J*_2,3_ 9.1 Hz, *J*_3,4_ 3.3 Hz, H-3), 4.10–3.98 (m, 3H, H-5, H-6a, H-6b), 3.91 (s, 3H, COO*Me*), 2.18, 2.16, 1.98, 1.60 (4s, each 3H, 4 × CO*Me*); ^13^C NMR (62.9 MHz, CD_3_CN) δ: 171.2, 171.1, 170.8, 170.7 (4 × *C*OMe), 160.5 (*C*OOMe), 140.5, 139.8, 139.5, 130.4 (4 × Ar-*C*), 130.1, 129.1, 128.2 (Ph-CH), 124.1–123.2 (*C*F_3_ + Ar-*C*), 120.3 (Ar-*C*H), 117.2 (Ar-*C*), 114.8, 106.8 (2 × Ar-*C*H), 106.2 (C-1), 72.1 (C-5), 71.3 (C-3), 68.4 (C-4), 68.1 (C-2), 62.4 (C-6), 52.9 (COOMe), 21.2, 20.8, 20.7, 20.4 (4 × COMe). Anal. Calcd for C_31_H_30_F_3_NO_12_: C, 55.94; H, 4.54%; N, 2.10%; Found: C, 55.97; H, 4.58%; N, 2.13%.

### 3.3. Methyl 1-[4-O-(2,3,4,6-Tetra-O-Acetyl-β-d-Galactopyranosyl)-2,3,6-Tri-O-Acetyl-β-d-Glucopyranosyl)]-6-Phenyl-4-Trifluoromethyl-1H-Indole-2-Carboxylate (**9β**)

The glycosylation of **8α** [12,13] (175 mg, 0.22 mmol, 1.5 equiv) with *N*-hydroxyindole derivative **1** [19] (50 mg, 0.15 mmol, 1.0 equiv), was performed in dry CH_2_Cl_2_ (4.0 mL) in according to the general procedure (Method B). The reaction was stirred until TLC (2 h, TLC, 1:1 hexane-EtOAc, double elution) showed the complete disappearance of the acceptor **1** (*R*_f_ 0.76) and the formation of one spot at *R*_f_ 0.45. Purification of crude product by flash chromatography on silica gel (1:1 hexane-EtOAc) afforded pure **9β** (93 mg, 65%) as solid; *R*_f_ 0.76 (7:3 hexane-EtOAc, double elution); mp 100–103 °C (crom); [α]_D_ +49.4 (*c* 1.0 in CHCl_3_); ^1^H NMR (250.13 MHz, CD_3_CN-CDCl_3_) δ: 8.08 (m, 1H, Ar-*H*), 7.80 (m, 1H, Ar-*H*), 7.71–7.66 (m, 2H, Ar-*H*), 7.58–7.38 (m, 3H, Ar-*H*), 7.22 (qd, 1H, *J* 1.0 Hz, *J* 1.8 Hz, Ar-*H*), 5.57 (d, 1H, *J*_1,2_ 8.0 Hz, 1-H), 5.33 (dd, 1H, *J*_2,3_ 9.8 Hz, *J*_3,4_ 8.7 Hz, 3-H), 5.30 (dd, 1H, *J*_3′,4′_ 3.5 Hz, *J*_4′,5′_ 1.3 Hz, H-4′), 5.23 (dd, 1H, H-2), 5.03 (dd, 1H, *J*_2′,3′_ 10.4 Hz, H-3′), 4.90 (dd, 1H, *J*_1′,2′_ 7.8 Hz, H-2′), 4.59 (d, 1H, H-1′), 4.19 (dd, 1H, *J*_5,6b_ 2.3 Hz, *J*_6a,6b_ 12.1 Hz, H-6b), 4.13–4.02 (m, 4H, H-6a, H-5′, H-6′a, H-6′b), 3.99 (dd, 1H, *J*_4,5_ 9.9 Hz, H-4), 3.91 (s, 3H, COO*Me*), 3.75 (ddd, 1H, *J*_5,6a_ 5.6 Hz H-5), 2.13, 2.09, 2.06, 2.02, 1.93, 1.89, 1.58 (7s, each 3H, 7 × CO*Me*); ^13^C NMR (62.9 MHZ, CD_3_CN-CDCl_3_) δ: 171.1–170.2 (7 × *C*OMe), 160.3 (*C*OOMe), 140.6, 139.8, 135.5, 130.4 (4 × Ar-*C*), 129.9, 128.9, 128.2 (Ph-*C*H), 123.9–122.9 (*C*F_3_ + Ar-*C*), 120.3 (Ar-*C*H), 117.3 (Ar-*C*), 114.9, 106.8 (2 × Ar-*C*H), 105.6 (C-1), 101.4 (C-1′), 76.9 (C-4), 73.4 (C-5), 72.7 (C-3), 71.5 (C-3′, C-5′), 70.5 (C-2), 69.8 (C-2′), 68.0 (C-4′), 62.7 (C-6′), 62.0 (C-6), 52.8 (COO*Me*), 21.1–20.4 (7 × COMe). Anal. Calcd for C_43_H_46_F_3_NO_20_: C, 54.15; H, 4.86%; N, 1.47%; Found: C, 54.18; H, 4.89%; N, 1.50%.

### 3.4. General Procedures for Mitsunobu Reaction

A solution of appropriate 6-OH-sugar **11** [19], **14 [20]**, **15** [21], or **20** [26] (1.00 mmol, 1.0 equiv) and the *N*-hydroxyindole derivative **1** (1.30 mmol, 1.3 equiv) in anhyd THF (20.0 mL) was cooled to 0 °C and treated with dry PPh_3_ (3.50 mmol, 3.5 equiv) and di-isopropyl azodicarboxylate (DIAD) or di-2-methoxyethyl azodicarboxylate (DMEAD) (3.50 mmol, 3.5 equiv). The reaction mixture was stirred at room temp until TLC analysis showed the complete disappearance of the starting compound. The reaction mixture was diluted with CH_2_Cl_2_ (35.0 mL) and washed with satd aq Na_2_HCO_3_ (35 mL). The aqueous phases were extracted with CH_2_Cl_2_ (2 × 25 mL) and the combined organic layers were dried over MgSO_4_, filtered and concentrated under diminished pressure. The crude residue was subjected to flash chromatography on silica gel.

### 3.5. Methyl 2,3,4-Tri-O-Acetyl-6-O-(2-Methoxycarbonyl-6-Phenyl-4-Trifluoromethyl-1H-Indol-1-Yl)-α-d-Mannopyranoside (**12**)

Mitsunobu reaction applied to **11** (120 mg, 0.38 mmol, 1.0 equiv) was performed with DIAD (263 μL, 1.33 mmol, 3.5 equiv) in accordance with the general procedure. The reaction was stopped after 3 h and the crude product was purified by flash chromatography on silica gel (3:7 hexane-EtOAc) gave pure **12** (108 mg, 46%) as a colorless oil; *R*_f_ 0.58 (1:1 hexane-EtOAc); [α]_D_ +34.2 (*c* 0.89 in CHCl_3_); ^1^H NMR (250.13 MHz, CD_3_CN) δ: 8.13 (m, 1H, Ar-*H*), 7.81 (m, 1H, Ar-*H*), 7.77–7.64 (m, 2H, Ar-*H*), 7.55–7.39 (m, 3H, Ar-*H*), 7.22 (qd, 1H, Ar-*H*), 5.33 (dd, 1H, *J*_3,4_ 10.2 Hz, *J*_4,5_ 9.8 Hz, H-4), 5.23 (dd, 1H, *J*_2,3_ 6.2 Hz, H-3), 4.90 (dd, 1H, *J*_1,2_ 1.4 Hz, H-2), 4.86 (d, 1H, H-1), 4.68 (dd, 1H, *J*_5,6b_ 5.6 Hz, *J*_6a,6b_ 10.1 Hz, H-6b), 4.52 (dd, 1H, *J*_5,6a_ 2.3 Hz, H-6a), 4.28 (ddd, 1H, H-5), 3.92 (s, 3H, COO*Me*), 3.44 (s, 3H, OMe), 2.04, 2.02, 1.95 (3s, each 3H, 3 × CO*Me*); ^13^C NMR (62.9 MHz, CD_3_CN) δ: 171.0–170.8 (3 × *C*OMe), 160.5 (*C*OOMe), 140.5, 139.4, 137.2, 130.1 (4 × Ar-*C*), 130.1, 129.1, 128.3 (Ph-*C*H), 124.3–123.3 (*C*F_3_ + Ar-*C*), 120.0 (Ar-*C*H), 117.6 (Ar-*C*), 112.7, 105.4 (2 × Ar-*C*H), 99.5 (C-1), 78.9 (C-6), 72.7 (C-2), 70.1 (C-3), 69.4 (C-5), 66.4 (C-4), 55.9 (OMe), 52.9 (COO*Me*), 22.1, 21.0, 20.9 (3 × COMe). Anal. Calcd for C_30_H_30_F_3_NO_11_: C, 56.52; H, 4.74%; N, 2.20%; Found: C, 56.55; H, 4.77%; N, 2.23%.

### 3.6. Methyl 2,3,4-Tri-O-Acetyl-6-O-(2-Methoxycarbonyl-6-Phenyl-4-Trifluoromethyl-1H-Indol-1-Yl)-β-d-Glucopyranoside (**16**)

Mitsunobu reaction applied to **14** (72 mg, 0.22 mmol, 1.0 equiv) was performed with DMEAD (186 mg, 0.80 mmol, 3.5 equiv) in accordance with the general procedure. The reaction was stopped after 4 h and the crude product was purified by flash chromatography on silica gel (65:35 hexane-EtOAc) gave pure **16** (104 mg, 86%) as a yellow solid; *R*_f_ 0.53 (1:1 hexane-AcOEt); mp 191–194 °C (crom); [α]_D_ –11.5 (*c* 1.0 in CHCl_3_); ^1^H NMR (250.13 MHz, CD_3_CN) δ: 8.04 (1H, m, Ar-*H*), 7.80 (m, 1H, Ar-*H*), 7.76 (m, 1H, Ar-*H*), 7.73 (m, 1H, Ar-*H*), 7.59–7.40 (m, 3H, Ar-*H*), 7.18 (qd, 1H, *J* 0.9 Hz, *J* 1.7 Hz, Ar-*H*), 5.27 (dd, 1H, *J*_2,3_ 9.8 Hz, *J*_3,4_ 9.5 Hz, H-3), 5.10 (dd, 1H, *J*_4,5_ 10.1 Hz, H-4), 4.93 (dd, 1H, *J*_1,2_ 8.0 Hz, H-2), 4.63 (dd, 1H, *J*_5,6b_ 5.8 Hz, *J*_6a,6b_ 10.8 Hz, H-6b), 4.56 (d, 1H, H-1), 4.49 (dd, 1H, *J*_5,6a_ 2.1 Hz, H-6a), 4.10 (ddd, 1H, H-5), 3.91 (s, 3H, COO*Me*), 3.33 (s, 3H, OMe), 2.02, 2.00, 1.95 (3s, each 3H, 3 × CO*Me*); ^13^C NMR (62.9 MHz, CD_3_CN): δ 170.9–170.4 (3 × *C*OMe), 160.5 (*C*OOMe), 140.5, 139.5, 137.2, 130.4 (4 × Ar-*C*), 130.0, 129.1, 128.3 (Ph-*C*H), 124.3–123.3 (*C*F_3_ + Ar-*C*), 120.1 (Ar-*C*H), 117.5 (Ar-C), 112.5, 105.4 (2 × Ar-*C*H), 102.1 (C-1), 78.4 (C-6), 73.5 (C-3), 72.5 (C-5), 71.9 (C-2), 69.2 (C-4), 57.3 (OMe), 52.9 (COOMe), 21.0–20.8 (3 × COMe). Anal. Calcd for C_30_H_30_F_3_NO_11_: C, 56.52; H, 4.74%; N, 2.20%; Found: C, 56.54; H, 4.76%; N, 2.24%.

### 3.7. Methyl 2,3,4-Tri-O-Acety-6-O-(2-Methoxycarbonyl)-6-Phenyl-4-Trifluoromethyl-1H-Indol-1-Yl)-β-d-Galactopyranoside (**17**)

Mitsunobu reaction applied to **15** [5] (120 mg, 0.38 mmol, 1.0 equiv) was performed with DMEAD (306 mg, 1.30 mmol, 3.5 equiv) in accordance with the general procedure. The reaction was stopped after 4 h and the crude product was purified by flash chromatography on silica gel (7:3 hexane-EtOAc) gave pure **17** (162 mg, 68%) as a yellow solid; *R*_f_ 0.55 (1:1 hexane-EtOAc); mp 169–172 °C (crom); [α]_D_ + 28.5 (*c* 0.96 in CHCl_3_); ^1^H NMR (250.13 MHz, CD_3_CN) δ: 7.99 (m, 1H, Ar-*H*), 7.78 (m, 1H, Ar-*H*), 7.77–7.72 (m, 2H, Ar-*H*), 7.54–7.38 (m, 3H, Ar-*H*), 7.18 (qd, 1H, *J* 1.0 Hz, *J* 1.7 Hz, Ar-*H*), 5.46 (dd, 1H, *J*_3,4_ 3.4 Hz, *J*_4,5_ 1.1 Hz, H-4), 5.10 (dd, 1H, *J*_2,3_ 10.5 Hz, H-3), 5.03 (dd, 1H, *J*_1,2_ 7.8 Hz, H-2), 4.64 (dd, 1H, *J*_5,6a_ 7.5 Hz, *J*_6a,6b_ 9.9 Hz, H-6a), 4.62 (dd, 1H, *J*_5,6b_ 3.1 Hz, H-6b), 4.51 (d, 1H, H-1), 4.35 (ddd, 1H, H-5), 3.91 (s, 3H, COO*Me*), 3.35 (s, 3H, OMe), 2.07, 2.01, 1.92 (3s, each 3H, 3 × CO*Me*); ^13^C NMR (62.9 MHz, CD_3_CN) δ: 171.4, 170.9, 170.7 (3 × *C*OMe), 160.5 (*C*OOMe), 140.5, 139.6, 137.2 130.8 (4 × Ar-*C*), 130.0, 129.5, 128.3 (Ph-*C*H), 124.3–123.2 (*C*F_3_ + Ar-*C*), 120.0 (Ar-*C*H), 117.4 (Ar-*C*), 112.5, 105.4 (2 × Ar-*CH*), 102.5 (C-1), 78.9 (C-6), 71.7 (C-3), 71.6 (C-5), 69.7 (C-2), 69.1 (C-4), 57.5 (OMe), 52.9 (COO*Me*), 20.9, 20.8, 20.7 (3 × CO*Me*). Anal. Calcd for C_30_H_30_F_3_NO_11_: C, 56.52; H, 4.74%; N, 2.20%; Found: C, 56.57; H, 4.75%; N, 2.25%.

### 3.8. 4-O-[2-O-Acetyl-3,4-O-Isopropylidene-6-O-(2-Methoxycarbonyl-6-Phenyl-4-Trifluoromethyl-1H-Indol-1-Yl)-β-d-Galactopyranosyl]-2,3:5,6-Di-O-Isopropylidene-Aldehydo-d-Glucose Dimethyl Acetal (**21**)

Mitsunobu reaction applied to **20** [7] (170 mg, 0.30 mmol, 1.0 equiv) was performed with DMEAD (330 mg, 1.40 mmol, 3.5 equiv) in accordance with the general procedure. The reaction was stopped after 3.5 h and the crude product was purified by flash chromatography on silica gel (7:3 hexane-EtOAc) gave pure **21** (200 mg, 75%) as a yellow solid foam; *R*_f_ 0.63 (1:1 hexane-EtOAc); mp 77–80 °C (crom); [α]_D_ -14.1 (*c* 1.0 in CHCl_3_); ^1^H NMR (250.13 MHz, CD_3_CN) δ: 8.05 (m, 1H, Ar-*H*), 7.77 (m, 1H, Ar-*H*), 7.76–7.72 (m, 2H, Ar-*H*), 7.56–7.40 (m, 3H, Ar-*H*), 7.25 (qd, 1H, *J* 0.9 Hz, *J* 1.7 Hz, Ar-*H*), 4.92 (dd, 1H, *J*_1′,2′_ 8.3 Hz, *J*_2′,3′_ 7.6 Hz, H-2′), 4.79 (d, 1H, H-1′), 4.73 (dd, 1H, *J*_5′,6′b_ 6.5 Hz, *J*_6′a,6′b_ 10.1 Hz, H-6′b), 4.68 (dd, 1H, *J*_5′,6′a_ 5.2 Hz, H-6′a), 4.39 (dd, 1H, *J*_1,2_ 5.6 Hz, *J*_2,3_ 6.7 Hz, H-2), 4.30 (d, 1H, H-1), 4.29–4.21 (m, 2H, H-4′, H-5′), 4.16 (dd, 1H, *J*_3′,4′_ 5.2 Hz, H-3′), 4.15–3.96 (m, 2H, H-3, H-5), 3.99 (dd, 1H, *J*_3,4_ 1.6 Hz, *J*_4,5_ 3.1 Hz, H-4), 3.96 (s, 3H, COO*Me*), 3.83 m, (2H, H-6a, H-6b), 3.29, 3.24 (2s, each 3H, 2 × OMe), 2.06 (s, 3H, CO*Me*), 1.49, 1.38 (2s, each 3H, C*Me_2_*), 1.28 (s, 6H, C*Me_2_*), 1.27, 1.25 (2s, each 3H, C*Me_2_*); ^13^C NMR (62.9 MHz, CD_3_CN): δ 170.3 (*C*OMe), 160.3 (*C*OOMe), 140.6, 139.8, 137.6, 131.4 (4 × Ar-*C*), 129.8, 128.9, 128.4 (Ph-*C*H), 124.7–123.5 (*C*F_3_ + Ar-*C*), 120.2 (Ar-*C*H), 117.3 (Ar-*C*), 112.3 (Ar-*C*H), 111.1, 110.8, 108.5 (3 × *C*Me_2_), 106.1 (C-1), 105.6 (Ar-CH), 101.0 (C-1′), 78.7 (C-6′), 78.4 (C-5), 78.2 (C-3), 77.6 (C-3′), 76.7 (C-2), 76.0 (C-4), 74.7 (C-4′), 73.3 (C-2′), 70.5 (C-5′), 65.2 (C-6), 56.2, 54.5 (2 × OMe), 52.8 (COO*Me*), 28.1, 27.7, 26.8, 26.5, 26.3, 24.8 (3 × C*Me_2_*), 21.1 (COMe). Anal. Calcd for C_42_H_52_F_3_NO_15_: C, 58.13; H, 6.04%; N, 1.61%; Found: C, 58.16; H, 6.07%; N, 1.64%.

### 3.9. General Procedures for Deacetylation

To a solution of appropriate acetylated NHI-glycoconjugates **6α**, **6β**, **9β**, **12**, **16**, or **17** (1.00 mmol, 1.0 equiv) in a 2:3 mixture of CH_2_Cl_2_-MeOH (46.0 mL) was cooled at 0 °C and treated with a methanolic solution of MeONa (0.33 M, 0.575 mL). The reaction mixture was stirred at room temp until TLC analysis showed the complete disappearance of the starting material (2–7 h) and the formation of a lower moving product. The solution was neutralized with Amberlite^®^ IR120 H resin and the suspension was filtered and concentrated under diminished pressure. Purification of crude product by crystallization or trituration with Et_2_O afforded pure NHI-glycoconjugates **7α**, **7β**, **10β**, **13**, **18**, or **19**.

### 3.10. Methyl 1-(α-d-Galactopyranosyl)-6-Phenyl-4-Trifluoromethyl-1H-Indole-2-Carboxylate (**7α**)

Deacetylation of **6α** (74 mg, 0.11 mmol, 1.0 equiv) was performed in accordance with the general procedure. The reaction was stirred until the starting compound was completely reacted (2 h, TLC, 9:1 EtOAc-MeOH). The crude residue (52 mg, 91% yield) was constituted (NMR) exclusively by a title compound **7α**. The trituration of the crude product (52 mg) with Et_2_O afforded a sample of pure **7α** (28 mg, 49%) as a yellow solid; *R*_f_ 0.54 (9:1 EtOAc-MeOH), mp 159–162 °C (Et_2_O); [α]_D_ -15.3 (*c* 0.63 in MeOH); ^1^H NMR (250.12 MHz, CD_3_OD-CDCl_3_) δ: 8.41 (m, 1H, Ar-*H*), 7.86–7.62 (m, 3H, Ar-*H*), 7.48–7.33 (m, 3H, Ar-*H*), 7.27 (bs, 1H, Ar-*H*), 5.37 (d, 1H, *J*_1,2_ 3.9 Hz, H-1), 4.51 (m, 1H, H-5), 4.15 (m, 1H, H-4), 4.08 (dd, 1H, *J*_2,3_ 10.6 Hz, H-2), 4.00 (dd, 1H, *J*_3,4_ 2.8 Hz, H-3), 3.95 (s, 3H, COO*Me*), 3.91 (bs, 1H, OH), 3.80–3.65 (m, 4H, H-6a, H-6b, 2 × OH), 3.36 (bs, 1H, OH); ^13^C NMR (62.9 MHz, CD_3_OD-CDCl_3_) δ: 161.5 (CO), 140.6, 139.6, 137.5, 131.9 (4 × Ar-*C*), 129.7, 128.7, 128.0 (Ph-*C*H), 125.2–123.0 (*C*F_3_ + Ar-*C*), 119.8 (Ar-*C*H), 118.1 (Ar-*C*), 113.4 (Ar-*C*H), 109.4 (C-1), 106.4 (Ar-CH), 75.1 (C-5), 70.4 (C-2), 69.9 (C-3), 69.3 (C-4), 61.9 (C-6), 52.8 (OMe). Anal. Calcd for C_23_H_22_F_3_NO_8_: C, 55.54; H, 4.46%; N, 2.82%; Found: C, 55.57; H, 4.49%; N, 2.86%.

### 3.11. Methyl 1-(β-d-Galactopyranosyl)-6-Phenyl-4-Trifluoromethyl-1H-Indole-2-Carboxylate (***7β***)

Deacetylation of **6β** (60 mg, 0.089 mmol, 1.0 equiv) was performed in accordance with the general procedure. The reaction was stirred until the starting compound was completely reacted (2 h, TLC, 9:1 EtOAc-MeOH). The crude residue (43 mg, 91% yield) was constituted (NMR) exclusively by a title compound **7β**. The trituration of the crude product with Et_2_O afforded a sample of pure **7β** (30 mg, 66%) as a white solid; *R*_f_ 0.83 (9:1 EtOAc-MeOH); mp 207–210 °C (Et_2_O); [α]_D_ +39.4 (*c* 0.505 in MeOH); ^1^H NMR (250.13 MHz, CD_3_OD-CDCl_3_) δ: 8.24 (bs, 1H, Ar-*H*), 7.71 (m, 1H, Ar-*H*), 7.68–7.73 (2m, each 1H, Ar-*H*), 7.48–7.39 (m, 2H, Ar-*H*), 7.38–7.32 (m, 1H, Ar-*H*), 7.27 (qd, 1H, Ar-*H*), 5.09 (d, 1H, *J*_1,2_ 8.1 Hz, H-1), 3.95 (m, 1H, H-2), 3.94 (s, 3H, COO*Me*), 3.81 (dd, 1H, *J*_5,6b_ 6.9 Hz, *J*_6a,6b_ 11.0 Hz, H-6b), 3.67 (dd, 1H, *J*_5,6a_ 5.5 Hz, H-6a), 3.62 (dd, 1H, *J*_2,3_ 9.4 Hz, *J*_3,4_ 3.4 Hz, H-3), 3.60 (m, 1H, H-4), 3.49 (ddd, 1H, *J*_4,5_ 0.8 Hz, H-5); ^13^C NMR (62.9 MHz, CD_3_OD-CDCl_3_) δ: 161.8 (CO), 140.5, 139.5, 137.4, 132.1 (4 × Ar-*C*), 129.6, 128.9, 128.4 (Ph-*C*H), 125.4–123.6 (*C*F_3_ + Ar-*C*), 119.7 (Ar-*C*H), 118.3 (Ar-*C*), 113.6 (Ar-*C*H), 110.3 (C-1), 106.8 (Ar-*C*H), 76.7 (C-5), 74.3 (C-3), 72.3 (C-2), 69.9 (C-4), 62.2 (C-6), 53.4 (COO*Me*). Anal. Calcd for C_23_H_22_F_3_NO_8_: C, 55.54; H, 4.46%; N, 2.82%; Found: C, 55.57; H, 4.49%; N, 2.85%.

### 3.12. Methyl 1-[4-O-(β-d-Galactopyranosyl)-β-d-Glucopyranosyl)]-6-Phenyl-4-Trifluoromethyl-1H-Indole-2-Carboxylate (***10β***)

Deacetylation of **9β** (53 mg, 0.055 mmol, 1.0 equiv) was performed in accordance with the general procedure. The reaction was stirred until the starting compound was completely reacted (8 h, TLC, 9:1 EtOAc-MeOH). The crude residue (37 mg, 96% yield) was constituted (NMR) exclusively by a title compound **10β**. The trituration of the crude product (37 mg) with Et_2_O afforded a sample of pure **10β** (27 mg, 74%) as a white solid: *R*_f_ 0.10 (9:1 EtOAc-MeOH); mp 199–202 °C. [α]_D_ +63.3 (*c* 0.59 in MeOH); ^1^H NMR (250.13 MHz, DMSO-*d*_6_) δ: 8.27 (m, 1H, Ar-*H*), 7.83–7.80 (m, 3H, Ar-*H*), 7.55–7.49 (m, 3H, Ar-*H*), 7.10 (bs, 1H, Ar-*H*), 5.78 (bd, 1H, *J* 5.0 Hz, OH), 5.14–5.01 (m, 2H, 2 × OH), 4.87–4.78 (m, 2H, 1-H, OH), 4.69–4.61 (m, 2H, 2 × OH), 4.56–4.43 (m, 2H, H-1′, OH), 4.31–4.16 (m, 3H, H-6a, H-6b, H-5′), 3.88 (s, 3H, COOMe), 3.46–3.22 (m, 9H, H-2, H-3, H-4, H-5, H-2′, H-3′, H-4′, H-6′a, H-6′b; ^13^C NMR (62.9 MHz, DMSO-*d*_6_) δ: 159.7 (CO), 139.0, 139.0, 137.0, 130.0 (4 × Ar-*C*), 129.2, 128.2, 127.3, 127.2 (Ph-*C*H), 124.0–121.8 (CF_3_ + Ar-*C*), 118.8 (Ar-*C*H), 116.7 (Ar-*C*), 113.5, 113.0 (2 × Ar-*C*H), 107.7 (C-1), 103.7 (C-1′), 79.4 (C-4), 75.6 (C-5′), 74.8, 74.3 (C-5, C-3), 73.3, 72.0 (C-2, C-3′), 70.5 (C-2′), 68.1 (C-4′), 60.4, 60.3 (C-6′, C-6), 52.4 (COO*Me*). Anal. Calcd for C_29_H_32_F_3_NO_13_: C, 52.81; H, 4.89%; N, 2.12%; Found: C, 52.84; H, 4.91%; N, 2.15%.

### 3.13. Methyl 6-O-(2-Methoxycarbonyl-6-Phenyl-4-Trifluoromethyl-1H-Indol-1-Yl)-α-d-Mannopyra-Noside (***13***)

Deacetylation of **12** (54 mg, 0.085 mmol, 1.0 equiv) was performed in accordance with the general procedure. The reaction was stirred until the starting compound was completely reacted (3.5 h, TLC, 9:1 EtOAc-MeOH). The crude residue (42 mg, 95% yield) was constituted (NMR) exclusively by a title compound **13**. The crystallization (Et_2_O-hexane) of crude product (42 mg) afforded a sample of pure **13** (32 mg, 73%) as a pale yellow solid; *R*_f_ 0.69 (9:1 EtOAc-MeOH); mp 59–62 °C (Et_2_O-hexane); [α]_D_ +21.6(*c* 0.92 in MeOH); ^1^H NMR (250.13 MHz, CD_3_OD-CDCl_3_) δ: 7.95 (bs, 1H, Ar-*H*), 7.67–7.61 (m, 3H, Ar-*H*), 7.49–7.32 (m, 3H, Ar-*H*), 7.16 (bs, 1H, Ar-*H*), 4.81 (bs, 1H, H-1), 4.64 (dd, 1H, *J*_5,6b_ 3.8 Hz, *J*_6a,6b_ 9.4 Hz, H-6b), 4.53 (dd, 1H, *J*_5,6a_ 1.8 Hz, H-6a), 4.19 (dd, 1H, *J*_3,4_ 9.6 Hz, *J*_4,5_ 9.4 Hz, H-4), 4.02 (bs, 1H, H-2), 3.93–3.75 (m, 5H, H-3, H-5, 3 × OH), 3.89 (s, 3H, COO*Me*), 3.35 (s, 3H, OMe); ^13^C NMR (62.9 MHz, CD_3_OD-CDCl_3_) δ: 161.2 (CO), 140.6, 139.5, 136.8, 131.9 (4 × Ar-*C*), 129.7, 128.6, 128.1 (3 × Ph-*C*H), 124.8–122.7 (*C*F_3_ + Ar-*C*), 120.1 (Ar-*C*H), 117.2 (Ar-*C*), 112.0, 106.4 (2 × Ar-*C*H), 101.9 (C-1), 78.1 (C-6), 72.1 (C-3), 71.2 (C-2), 71.0 (C-5), 67.5 (C-4), 55.9 (OMe), 51.3 (COO*Me*). Anal. Calcd for C_24_H_24_F_3_NO_8_: C, 56.36; H, 4.73%; N, 2.74%; Found: C, 56.39; H, 4.75%; N, 2.77%.

### 3.14. Methyl 6-O-(2-Methoxycarbonyl-6-Phenyl-4-Trifluoromethyl-1H-Indol-1-Yl)-β-d-Glucopyra-Noside (***18***)

Deacetylation of **16** (52 mg, 0.081 mmol, 1.0 equiv) was performed in accordance with the general procedure. The reaction was stirred until the starting compound was completely reacted (3.5 h, TLC, 9:1 EtOAc-MeOH). The crude residue (40 mg, 96% yield) was constituted (NMR) exclusively by a title compound **18**. The trituration of the crude product (40 mg) with Et_2_O afforded a sample pure **18** (18 mg, 43% yield) as a white solid; *R*_f_ 0.53 (9:1 EtOAc-MeOH); mp 211–214 °C (Et_2_O); [α]_D_ -73.7 (*c* 0.66 in MeOH); ^1^H NMR (250.13 MHz, CD_3_OD) δ: 8.10 (m, 1H, Ar-*H*), 7.70–7.62 (m, 3H, Ar-*H*), 7.50–7.38 (m, 3H, Ar-*H*), 7.15 (qd, 1H, Ar-*H*), 4.79 (dd, 1H, *J*_5,6b_ 1.6 Hz, *J*_6a,6b_ 10.5 Hz, H-6b), 4.58 (dd, 1H, *J*_5,6a_ 6.3 Hz, H-6a), 4.18 (d, 1H, *J*_1,2_ 7.7 Hz, H-1), 3.95 (s, 3H, COO*Me*), 3.70 (ddd, 1H, *J*_4,5_ 9.8 Hz, H-5), 3.41–3.30 (m, 2H, H-3, H-4), 3.36 (s, 3H, OMe), 3.24 (dd, 1H, *J*_2,3_ 9.4 Hz, H-2); ^13^C NMR (62.9 MHz, CD_3_OD) δ: 161.2 (CO), 141.0, 140.1, 137.8, 131.8 (4 × Ar-*C*), 130.1, 129.1, 128.4 (Ph-*C*H), 124.9–123.2 (*C*F_3_ + Ar-*C*), 119.9 (Ar-*C*H), 117.8 (Ar-*C*), 112.7 (Ar-*C*H), 105.8 (C-1), 105.4 (Ar-CH), 80.1 (C-6), 77.8, 71.3 (C-3, C-4), 75.5 (C-5), 74.9 (C-2), 57.5 (OMe), 52.7 (COO*Me*). Anal. Calcd for C_24_H_24_F_3_NO_8_: C, 56.36; H, 4.73%; N, 2.74%; Found: C, 56.40; H, 4.76%; N, 2.78%.

### 3.15. Methyl 6-O-(2-Methoxycarbonyl-6-Phenyl-4-Trifluoromethyl-1H-Indol-1-Yl)-β-d-Galactopyra-Noside (***19***)

Deacetylation of **17** (68 mg, 0.11 mmol, 1.0 equiv) was performed in accordance with the general procedure. The reaction was stirred until the starting compound was completely reacted (3 h, TLC, 9:1 EtOAc-MeOH). The crude residue (52 mg, 96% yield) was constituted (NMR) exclusively by a title compound **19**. The trituration of the crude product (52 mg) with Et_2_O afforded a sample pure **19** (41 mg, 76%) as a white solid; *R*_f_ 0.60 (9:1 EtOAc-MeOH); mp 191–194 °C (Et_2_O); [α]_D_ –21.6 (*c* 0.70 in MeOH); ^1^H NMR (250.13 MHz, CD_3_OD-CDCl_3_) δ: 7.99 (m, 1H, Ar-*H*), 7.68–7.62 (m, 3H, Ar-*H*), 7.47–7.32 (m, 3H, Ar-*H*), 7.24 (m, 1H, Ar-*H*), 4.74 (dd, 1H, *J*_5,6b_ 3.7 Hz, *J*_6a,6b_ 9.5 Hz, H-6b), 4.59 (dd, 1H, *J*_5,6a_ 7.2 Hz, H-6a), 4.15 (d, 1H, *J*_1,2_ 7.6 Hz, H-1), 4.01 (m, 1H, H-5), 3.96 (m, 1H, H-3), 3.93 (s, 3H, COO*Me*), 3.53 (m, 2H, H-2, H-4), 3.44 (s, 3H, OMe); ^13^C NMR (62.9 MHz, CD_3_OD-CDCl_3_): δ 160.6 (CO), 140.3, 139.4, 139.3, 131.7 (4 × Ar-*C*), 129.2, 128.3, 127.7 (Ph-*C*H), 124.7–123.3 (*C*F_3_ + Ar-*C*), 119.6 (Ar-*C*H), 117.2 (Ar-*C*), 111.6, 105.8 (2 × Ar-*C*H), 104.5 (C-1), 79.0 (C-6), 73.5 (C-2), 72.5 (C-5), 71.3 (C-4), 69.5 (C-3), 57.2 (OMe), 52.4 (COO*Me*). Anal. Calcd for C_24_H_24_F_3_NO_8_: C, 56.36; H, 4.73%; N, 2.74%; Found: C, 56.38; H, 4.76%; N, 2.78%.

### 3.16. 4-O-[3,4-O-Isopropylidene-6-O-(2-Methoxycarbonyl-6-Phenyl-4-Trifluoromethyl-1H-Indol-1-Yl)-β-d-Galactopyranosyl]-2,3:5,6-Di-O-Isopropylidene-Aldehydo-d-Glucose Dimethyl Acetal (***22***)

Deacetylation of **21** (75 mg, 0.087 mmol, 1.0 equiv) was performed in accordance with the general procedure. The reaction was stirred until the starting compound was completely reacted (8 h, TLC, 1:1 hexane-EtOAc). Purification of the crude product by flash chromatography on silica gel (1:1 hexane-EtOAc) gave pure **22** (67 mg, 93%) as a white foam; *R*_f_ 0.43 (1:1 hexane-EtOAc); mp 70–73 °C (chrom); [α]_D_ -23.2 (*c* 1.1 in CHCl_3_); ^1^H NMR (250.13 MHz, CD_3_CN) δ: 8.03 (m, 1H, Ar-*H*), 7.79 (bs, 1H, Ar-*H*), 7.76–7.71 (m, 2H, Ar-*H*), 7.54–7.39 (m, 3H, Ar-*H*), 7.23 (qd, 1H, *J* 1.0 Hz, *J* 1.7 Hz, Ar-*H*), 4.70 (dd, 1H, *J*_5′,6′b_ 6.9 Hz, *J*_6′a,6′b_ 9.4 Hz, H-6′b), 4.63 (dd, 1H, *J*_5′,6′a_ 5.0 Hz, H-6′a), 4.51 (d, 1H, *J*_1′,2′_ 8.1 Hz, H-1′), 4.46 (dd, 1H, *J*_1,2_ 5.9 Hz, *J*_2,3_ 7.2 Hz, H-2), 4.30 (dd, 1H, *J*_4′,5′_ 2.2 Hz, H-5′), 4.29 (d, 1H, H-1), 4.23 (dd, 1H, *J*_3′,4′_ 5.5 Hz, H-4′), 4.15 (m, 1H, H-5), 4.14–4.00 (m, 3H, H-6a, H-6b, H-3′), 4.03 (dd, 1H, *J*_3,4_ 1.6 Hz, H-3), 3.94 (s, 3H, COO*Me*), 3.88 (dd, 1H, *J*_4,5_ 4.0 Hz, H-4), 3.55 (bs, 1H, 2-OH), 3.43 (m, 1H, H-2′), 3.27, 3.20 (2s, each 3H, 2 × OMe), 1.46, 1.37, 1.33, 1.29, 1.27, 1.26 (6s, each 3H, 3 × C*Me*_2_); ^13^C NMR (62.9 MHz, CD_3_CN) δ: 160.4 (*C*O), 140.7, 139.8, 137.5, 131.4 (4 × Ar-*C*), 130.0, 129.1, 128.6 (Ph-*C*H), 124.7–123.3 (*C*F_3_ + Ar-*C*), 120.3 (Ar-*C*H), 117.5 (Ar-*C*), 112.6 (Ar-*C*H), 110.7, 110.5, 109.0 (3 × *C*Me_2_), 106.2 (C-1), 105.5 (Ar-*C*H), 104.4 (C-1′), 80.0 (C-3), 78.6 (C-6′), 78.5 (C-3′), 78.3 (C-4), 77.8 (C-5), 74.5 (C-4′), 74.4 (C-2′), 72.6 (C-2), 71.0 (C-5′), 65.9 (C-6), 56.3, 54.2 (2 × OMe), 52.9 (COO*Me*), 28.4, 27.5, 27.0, 26.5, 26.3, 25.0 (3 × C*Me*_2_). Anal. Calcd for C_40_H_50_F_3_NO_14_: C, 58.18; H, 6.10%; N, 1.70%; Found: C, 58.21; H, 6.13%; N, 1.74%.

### 3.17. 4-O-[6-O-(2-Methoxycarbonyl-6-Phenyl-4-Trifluoromethyl-1H-Indol-1-Yl)-β-d-Galactopyra-Nosyl]-α,β-d-Glucopyranose (***23***)

A solution of **22** (59 mg, 0.072 mmol, 1.0 equiv) in 80% aq AcOH (1.3 mL) was stirred at 80 °C. After 4 h, the TLC analysis (9:1 EtOAc-MeOH) showed the complete disappearance of the starting material and the formation of a lower moving product (*R*_f_ 0.10). The reaction mixture was concentrated under diminished pressure and repeatedly coevaporated with toluene (4 × 20 mL). Trituration of the residue (45 mg) with Et_2_O afforded an amorphous white solid (23 mg, 48% yield) composed (^13^C NMR, DMSO-d_6_) by a α-anomer **23** (>95%). ^1^H NMR (250.13 MHz, DMSO-*d*_6_) δ: 8.15 (m, 1H, Ar-*H*), 7.92–7.83 (m, 3H, Ar-*H*), 7.57–7.41 (m, 3H, Ar-*H*), 7.15 (m, 1H, Ar-*H*), 6.40 (d, 1H, *J*_1,OH_ 4.6 Hz, OH-1), 5.23 (d, 1H, *J* 7.2 Hz, OH), 4.92 (m, 2H, 2 × OH), 4.83 (d, 1H, *J*_1,2_ 5.1 Hz, H-1), 4.72–4.67 (m, 2H, 2 × OH), 4.58–4.47 (m, 3H, H-6′a, H-6′b*,* OH), 4.34 (d, 1H, *J*_1′,2′_ 7.2 Hz, H-1′), 4.12 (bt, 1H, H-5′), 3.91 (s, 3H, COO*Me*), 3.78–3.48 (m, 5H, H-2, H-3, H-6a, H-6b, H-3′), 3.40–3.20 (m, 4H, H-4, H-5, H-4′, H-2′); ^13^C NMR (62.9 MHz, DMSO-*d*_6_) δ: 159.0 (CO), 138.8, 138.2, 135.8, 129.2 (4 × Ar-*C*), 129.3, 128.1, 127.6 (Ph-*C*H), 123.5–122.3 (*C*F_3_ + Ar-*C*), 118.8 (Ar-*C*H), 115.9 (Ar-*C*), 111.8, 104.1 (2 × Ar-*C*H), 104.0 (C-1′), 92.1 (C-1), 82.2 (C-4), 79.4 (C-6′), 72.7 (C-2′), 72.3 (C-5′), 72.0 (C-5), 71.7 (C-3), 70.3 (C-4′), 69.8 (C-2), 68.9 (C-3′), 60.7 (C-6), 52.4 (COO*Me*). Anal. Calcd for C_29_H_32_F_3_NO_13_: C, 52.81; H, 4.89%; N, 2.12%; Found: C, 52.84; H, 4.92%; N, 2.15%.

### 3.18. Enzymatic Assays

The LDH inhibition activities of the compounds were evaluated against purified human lactate dehydrogenase isoform 5 (Lee Biosolution, Inc.). The ‘forward’ direction (pyruvate→lactate) of the lactate dehydrogenase reaction was conducted, and the IC_50_ measurements were performed by fluorescence (emission wavelength at 460 nm, excitation wavelength at 340 nm) to monitor the amount of consumed NADH. Assays were carried out in wells containing 200 μL of a reagents solution, dissolved in 100 mM phosphate buffer (pH = 7.4), in the presence of 200 μM pyruvate and 40 μM NADH. For the IC_50_ calculations of the compounds, seven different concentrations (in duplicate for each concentration) were used to produce the concentration–response curve. As well as the sample test wells, maximum and minimum controls were also included in each plate. After 15 min of incubation, the final measurements were carried out by using a Victor X3 Microplates Reader (PerkinElmer^®^). IC_50_ values were produced using GraphPad Prism software (GraphPad, San Diego, CA, USA).

### 3.19. Molecular Modeling Studies

The seven compounds were built using Maestro 9.0 and were minimized using the conjugate gradient method until a convergence value of 0.05 kcal (mol Å)^−1^ was reached. The minimization was carried out in a water environment model (generalized-Born/surface-area model) using the MMFF force field and a distance-dependent dielectric constant of 1.0. The *h*LDH5 chain was extracted from the minimized average structure previously obtained by us [14]. AUTODOCK 4.2 software [32] was employed for molecular docking. The identification of the torsion angles in the ligands, the addition of the solvent model and the determination of protein and ligand atomic charges was carried out using Autodock Tools. Kollmann charges were assigned to the protein and Gasteiger charges to the ligand. A grid spacing of 0.375 Å and a distance-dependent function of the dielectric constant were used for the energetic map calculations. The compounds were subjected to a robust docking procedure by applying 200 runs of Autodock search, using the Lamarckian Genetic Algorithm with 10,000,000 steps of energy evaluations [33]. The number of individuals in the initial population was set to 500 and a maximum of 10,000,000 generations were simulated during each docking run.

### 3.20. Cancer Cell Antiproliferative Potency Assays

Determination of cellular IC_50_ values was performed under normoxic conditions as described previously [10]. Briefly, HeLa and A549 cells, grown in RPMI 1640 medium supplemented with 10% FBS and 1% Penicillin/Streptomycin, were added at a density of 5000 cells per well to 96 well plates to which 31.6 nM–200 µM compound in DMSO was already added (1% final concentration DMSO in all wells; triplicate wells at the same concentration per repetition). Plates were incubated at 37 °C in a 95% air/5% CO_2_ atmosphere for 72 h. Medium was removed and cells were fixed by the addition of 50 µL 10% trichloroacetic acid in water, 4 °C, to each well. Plates were incubated at 4 °C for at least one hour, and the sulforhodamine B colorimetric assay was performed to assess remaining biomass in each well. Cells treated with 1% DMSO were used as the 100% live control for biomass, and wells incubated with medium alone were used as the baseline zero biomass control. IC_50_ values were calculated using SoftMax Pro software (Molecular Devices, Sunnyvale, CA).

### 3.21. Cellular Lactate Production Inhibition Assays

This assay was performed under normoxic conditions as previously described [10]. Briefly, confluent HeLa cervical carcinoma cells (ATCC, Manassas, VA, USA) in a 96-well plate were treated with compound or vehicle control (1% DMSO final concentration in all samples) in DMEM minus phenol red + 10% dialyzed FBS + 1% Penstrep, supplemented with 10 mM glucose, 1 mM sodium pyruvate and 4 mM glutamine, in a final volume of 125 µL per well. Immediately following compound addition, plates were incubated for 8 h at 37 °C in a 95% air/5% CO_2_ atmosphere. Duplicate wells were prepared for each treatment. Following treatment, medium was collected, and 100 µL were added to 2 µL 50 mM chlorophenylalanine (CPA; internal standard for GC-MS analysis). Samples were concentrated, derivatized by a four-hour incubation with MTBSTFA + 1% TBDMCS (Thermo Scientific, Walthman, MA, USA) in acetonitrile at 85 °C, and immediately analyzed using GC-MS (Agilent 6890N GC/5973 MS, equipped with an Agilent DB-5 capillary column, 30 M × 320 µM × 0.25 µM, model number J&W 123–5032, Agilent Technologies, Santa Clara, CA, USA) and an electron impact ionization source. One microliter of each sample was injected using an automated injector, and a solvent delay of 8.20 min was implemented. The initial oven temperature was 120 °C, held for 5 min; then the temperature was increased at a rate of 10 °C min^−1^ until a temperature of 250 °C was reached. Temperature was then increased by 40 °C min^−1^ until a final temperature of 310 °C was reached. Total run time per sample was 22.5 min. Compounds were identified using AMDIS Chromatogram software (Amdis, freeware available from amdis.net) and programmed WIST and Niley commercial libraries. The integration area of lactate in each sample was divided by the integration area of CPA in the same sample to achieve a lactate/internal standard ratio. The ratios were averaged for duplicates, and percent lactate production over the vehicle was calculated for each independent experiment. The mean lactate production/vehicle was then averaged between three or more independent experiments.

## 4. Conclusions

Seven NHI glycoconjugates, which differ for the kind of sugar and for the conjugation branching point, were prepared and studied as potential LDH inhibitors. The chemical strategy was aimed at successfully accessing a wide structural variation while keeping the synthetic plan simple. Therefore, C-6 and C-1 were selected as the two anchoring options, as it is quite straightforward to functionalize these positions in a large variety of carbohydrates. For the C-6 glycoconjugation, the Mistunobu protocol was employed, while classical trichloroacetimidate precursors were employed in the C-1 glycosylation reactions. For the galactose donor, it was possible to switch the glycosylation stereochemical outcome by changing the nature of the solvent used. Indeed, despite the presence of an acetate participating group at C-2, the alpha anomer was obtained with high stereoselectivity by exploiting the nitrile solvent effect. After cleavage of the protecting groups, the biological results of the resulting glycoconjugates revealed that only those linked at the anomeric carbon inhibited *h*LDH5, whereas conjugates at C(6) were completely devoid of any enzyme inhibitory activity. Among the anomerically-linked conjugates, monosaccharide derivatives **7α** and **7β** displayed potent antiproliferative activities against lung and cervical cancer cells, which are comparable to those observed with reference gluco-conjugate **2**, whereas disaccharide-linked **10β** proved to be much less potent in the same assays. Finally, the configuration of the anomeric carbon atom seems to strongly affect the ability of these compounds to reduce the cellular production of lactate. In fact, an α-anomeric configuration (**7α**) proved to be positively correlated to a significant and dose-dependent inhibition of lactate production in HeLa cancer cells, when compared to its β- configured anomer (**7β**). Overall, these results shed further light about the structural motifs of glycoconjugates that can be further developed with the aim of exploiting the Warburg effect and selectively counteract the peculiar metabolism displayed by cancer cells. The studies reported here, therefore, have the potential to help develop therapeutic agents characterized by fewer side effects.

## Figures and Tables

**Table 1:** Glycosylation of acceptor **1** with glycosyl donors **5α** and **6α**.

Glycosyl Donor	Promoter	Solvent	Anomeric Ratio *	Isolated Yield
**5α**	TMSOTf	CH_2_Cl_2_	6α:6β = 35:65	6α: 29% 6β: 42%
**5α**	TMSOTf	MeCN	6α:6β = 78:22	6α: 54% 6β: 13%
**5α**	BF_3_·Et_2_O	CH_2_Cl_2_	6α:6β = 5:95	6α: 3%6β: 78%
**8α**	BF_3_·Et_2_O	CH_2_Cl_2_	9α:9β < 1:99	9β: 65%

* Ratios were determined by NMR analysis of the crude product on the basis of the relative H-1 signal intensities.

**Table 2:** *h*LDH5 inhibition activities of glycoconjugates.

Compound	*h*LDH5 (IC_50_, μM) ^a^
**7α**	229 ± 16
**7β**	246 ± 36
**10β**	232 ± 47
**13**	>500
**18**	>500
**19**	>500
**23**	>500
**Galloflavin** ^b^	110 ± 4.0

^a^ Values are reported as the means ± SD of three or more independent experiments. ^b^ Reference *h*LDH5 inhibitor [29].

**Table 3:** Antiproliferative effect (72 h IC_50_ values, µM ± standard error, *n* = 3) of glycoconjugates in HeLa and A549 human cancer cell lines.

Compound	A549 (NSCLC)	HeLa (Cervical Carcinoma)
	IC_50_, μM	
**7α**	20.4 ± 2.1	7.5 ± 1.1
**7β**	17.6 ± 2.0	7.6 ± 1.1
**10β**	>100	81.5 ± 7.1
**2** ^a^	17.2 ± 3.0	7.2 ± 0.2

^a^ Data from [9].

## Supplementary Materials

The following are available online at https://www.mdpi.com/1420-3049/24/19/3520/s1, NMR spectra of prepared compounds and binding disposition of **7β**, **10β**, **13**, **18**, **19**, **23** into *h*LDH5.
