# Supplementary material for: Synthesis and Biological Evaluation of New Glycoconjugated LDH Inhibitors as Anticancer Agents
Source: Molecules. 2019 Sep 28;24(19):3520. doi: 10.3390/molecules24193520 (PMC6804087; doi:10.3390/molecules24193520)
Supplement: Supplementary file 1 [file molecules-24-03520-s001.pdf]

Supporting Information File 1

for

Synthesis and biological evaluation of new glycoconjugated LDH inhibitors as anticancer agents

Felicia D'Andrea[1,*], Giulia Vagelli[2], Carlotta Granchi[1], Lorenzo Guazzelli[1], Tiziano Tuccinardi[1], Giulio Poli[1], Dalila Iacopini[2], Filippo Minutolo[1] and Valeria Di Bussolo[2,*]

[1]*Dipartimento di Farmacia, Università di Pisa, Via Bonanno 33, 56126 Pisa, Italy;*

[2]*Dipartimento di Chimica e Chimica Industriale, Università di Pisa, Via G. Moruzzi 3, 56124 Pisa, Italy*

**Corresponding authors: felicia.dandrea@unipi.it, valeria.dibussolo@unipi.it.*

Contents

^{1}H-, ^{13}C NMR, and HSQC spectrum of **6α** S2-S3

^{1}H- and ^{13}C NMR spectrum of **6β** S3-S4

^{1}H- and ^{13}C NMR spectrum of **7α** S4-S5

^{1}H NMR and COSY spectrum of **7β** S5-S6

^{1}H-, ^{13}C NMR, COSY and HSQC spectrum of **9β** S6-S8

^{1}H- and ^{13}C NMR spectrum of **10β** S8-S9

^{1}H- and ^{13}C NMR spectrum of **12** S9-S10

^{1}H-, ^{13}C NMR and HSQC spectrum of **13** S10-S11

^{1}H-, ^{13}C-NMR, COSY and HSQC spectrum of **16** S12-S13

^{1}H- and ^{13}C-NMR, COSY and HSQC spectrum of **17** S14-S15

^{1}H-, ^{13}C-NMR and HSQC spectrum of **18** S16-S17

^{1}H-, ^{13}C-NMR and COSY spectrum of **19** S17-S18

^{1}H-, ^{13}C-NMR and HSQC spectrum of **21** S19-S20

^{1}H-, ^{13}C-NMR, COSY and HSQC spectrum of **22** S20-S22

^{1}H-, ^{13}C-NMR and COSY spectrum of **23** S22-S23

Binding disposition of **7β**, **10β**, **13**, **18**, **19**, **23** into *h*LDH5 (Fig. S1-S3) S24-S25

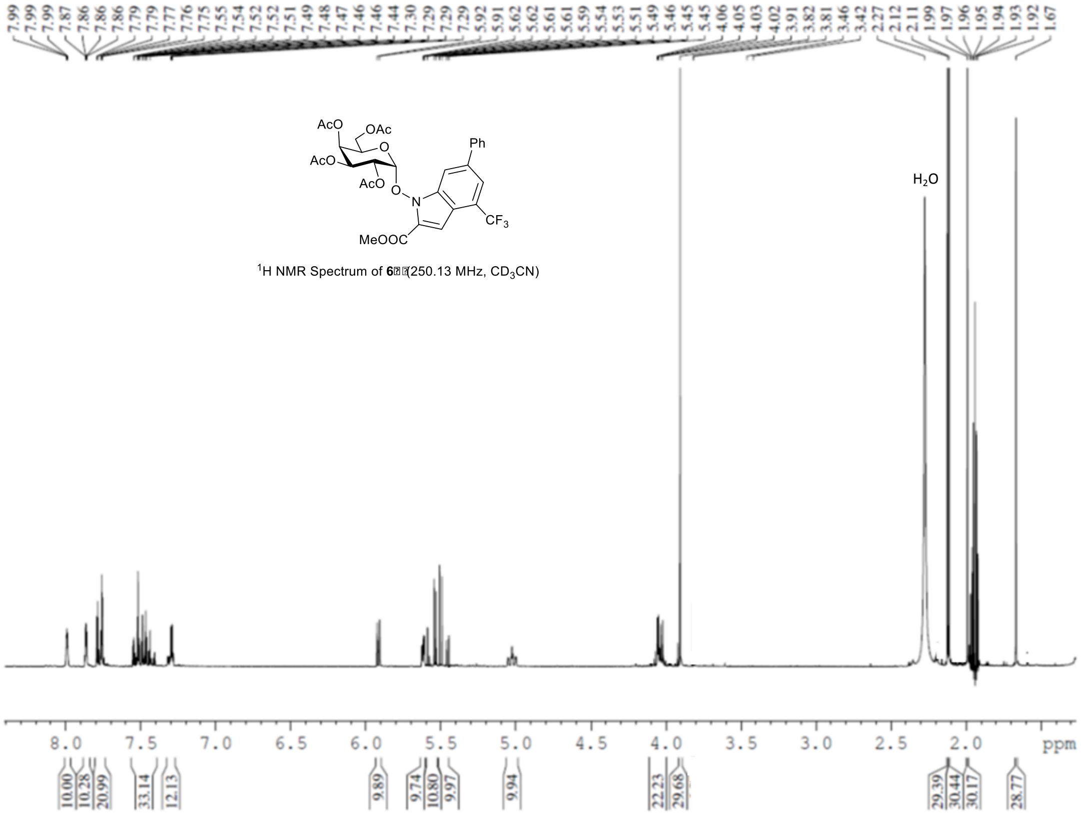

^1H NMR Spectrum of **6**[illegible](250.13 MHz, CD_3CN)

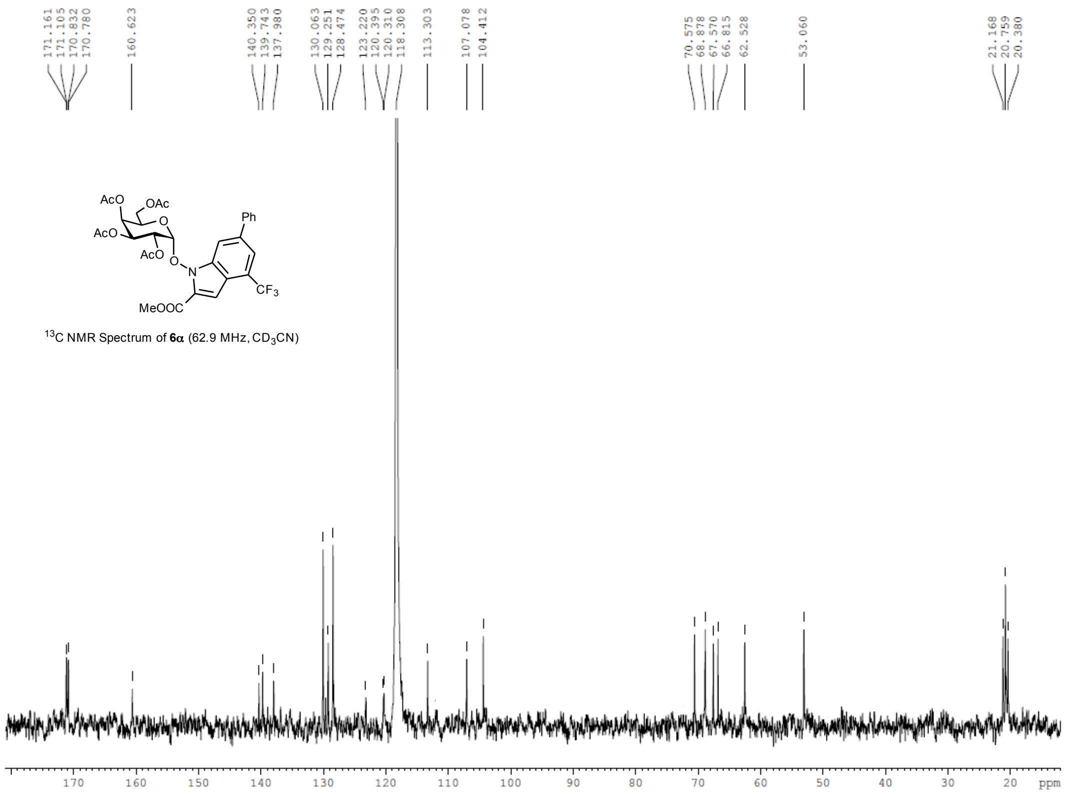

^{13}C NMR Spectrum of **6α** (62.9 MHz, CD_3CN)

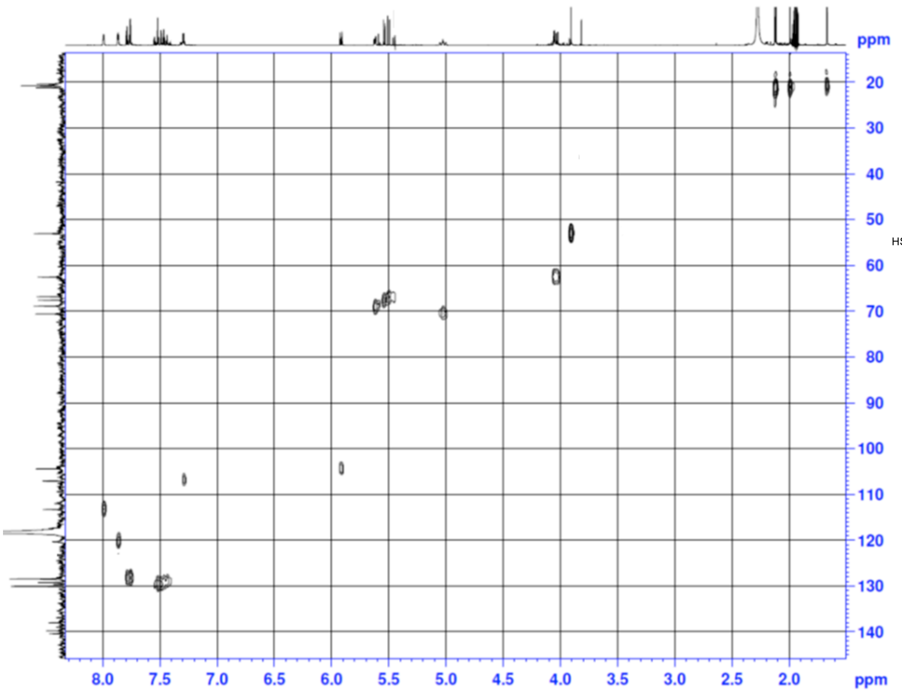

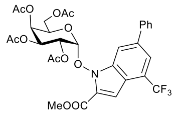

HSQC Spectrum of **6**⍰ (250.13 MHz, CD_3CN)

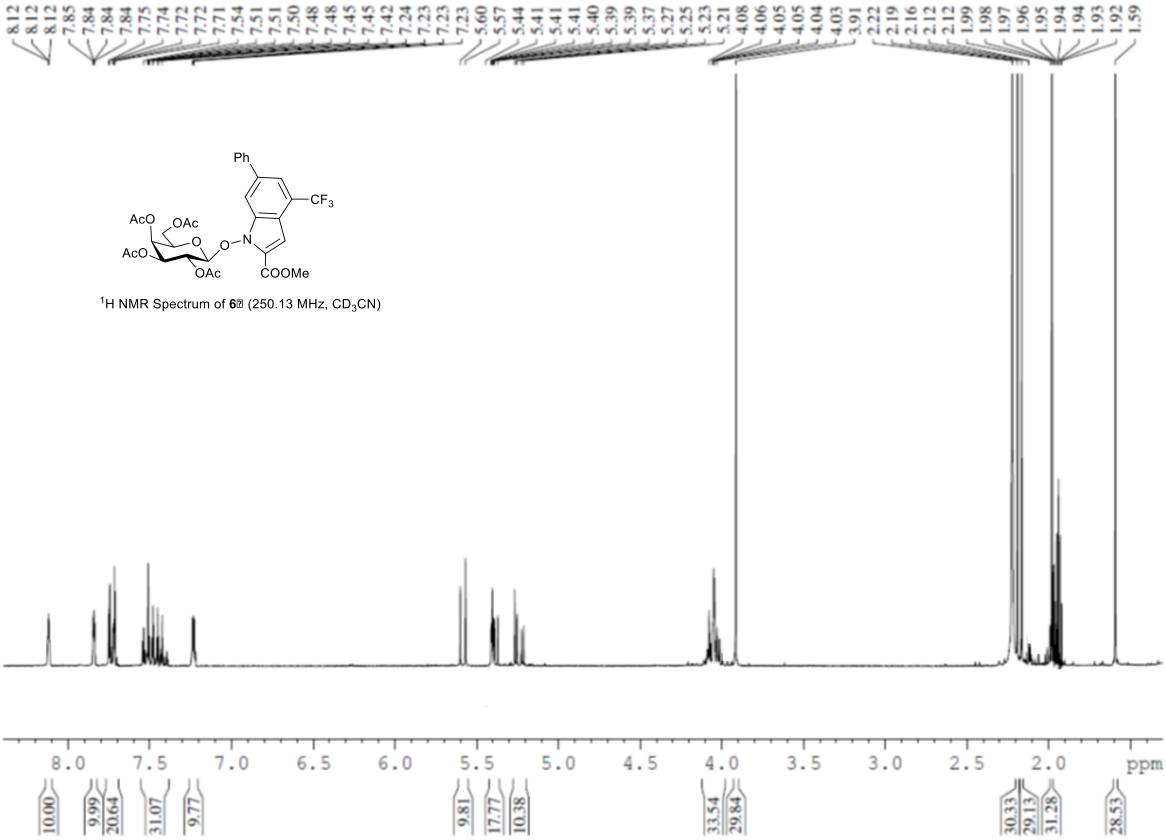

^{1}H NMR Spectrum of **6**⍰ (250.13 MHz, CD_3CN)

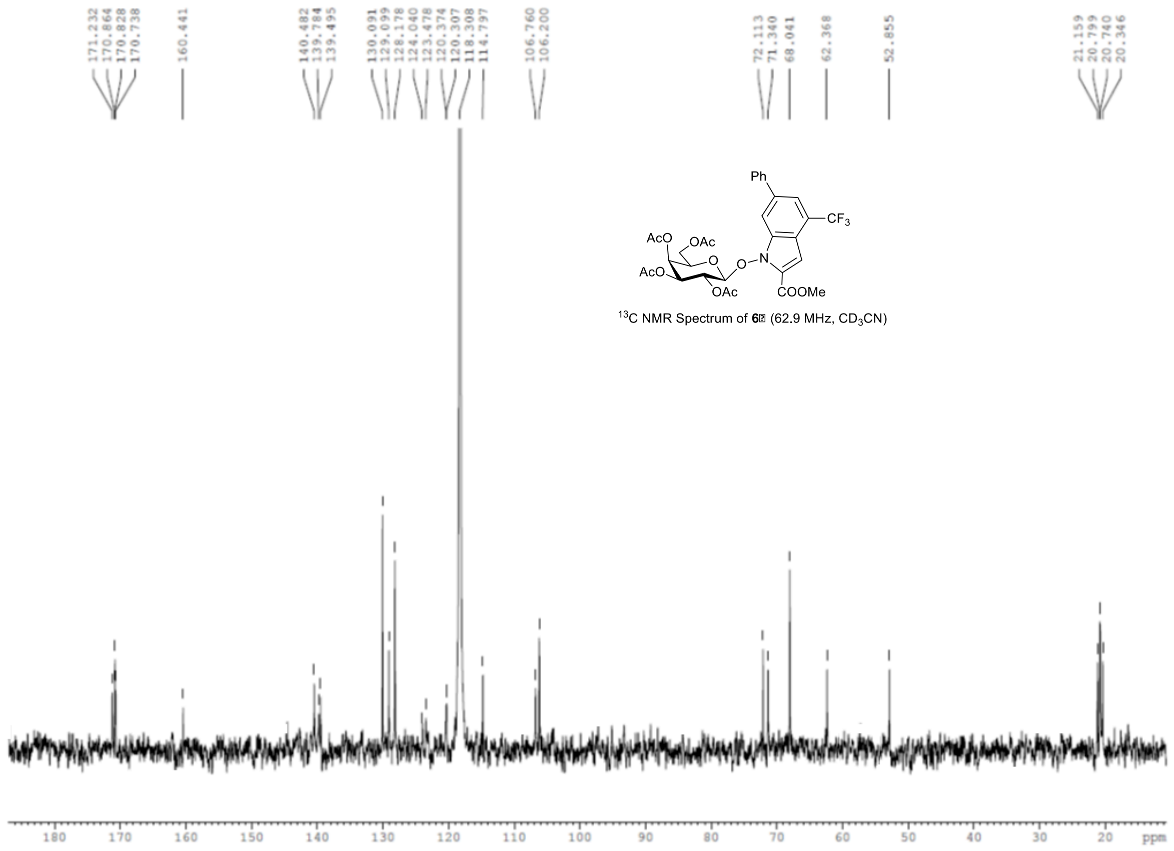

^{13}C NMR Spectrum of **6**⍰ (62.9 MHz, CD_3CN)

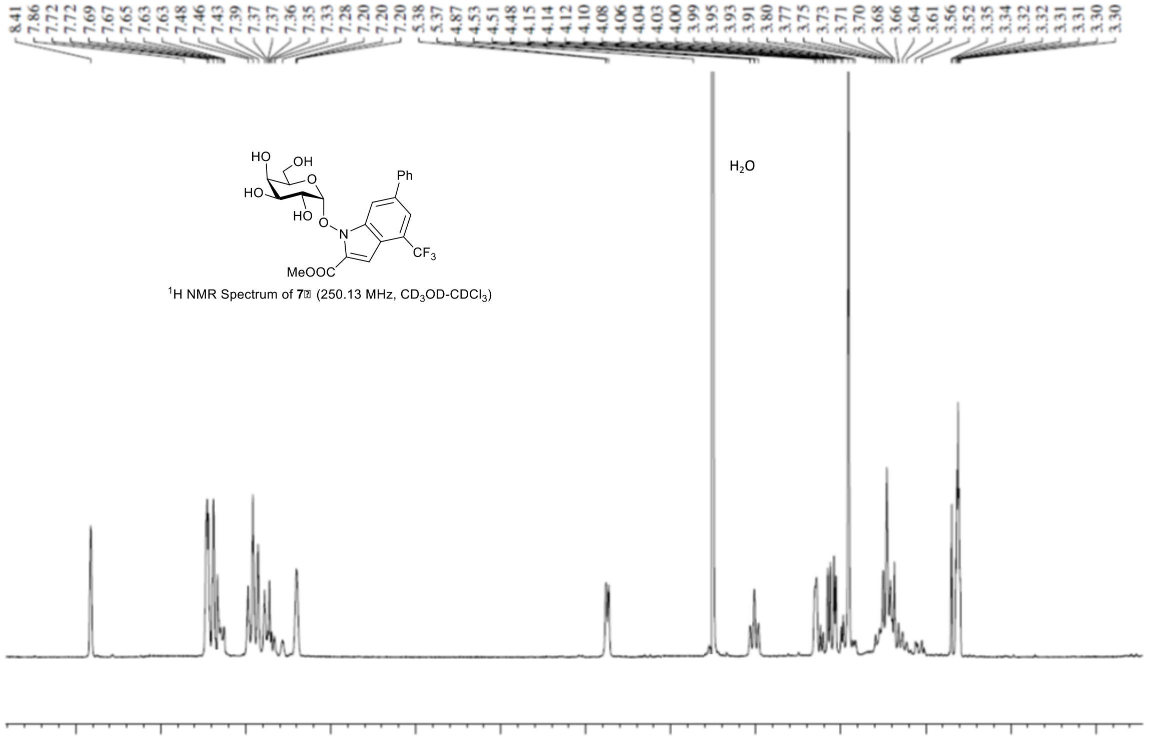

^{1}H NMR Spectrum of **7**⍰ (250.13 MHz, CD_3OD-$CDCl_3$)

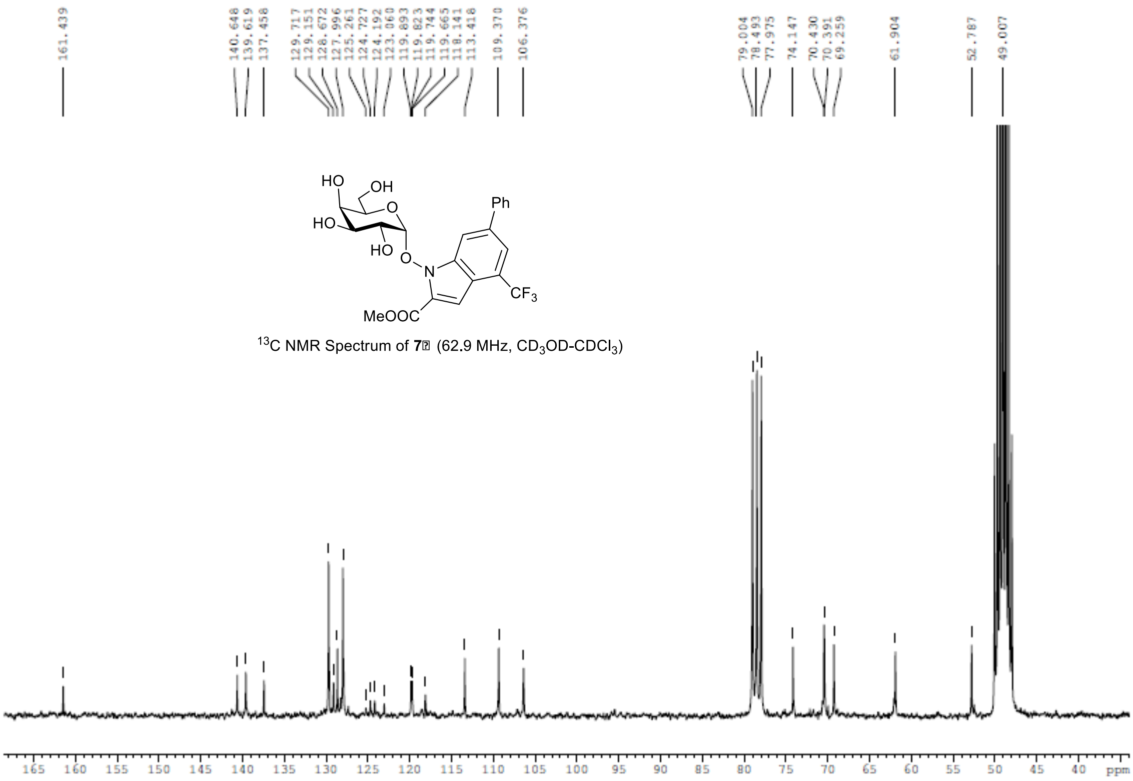

^{13}C NMR Spectrum of **7**[illegible] (62.9 MHz, CD_3OD-$CDCl_3$)

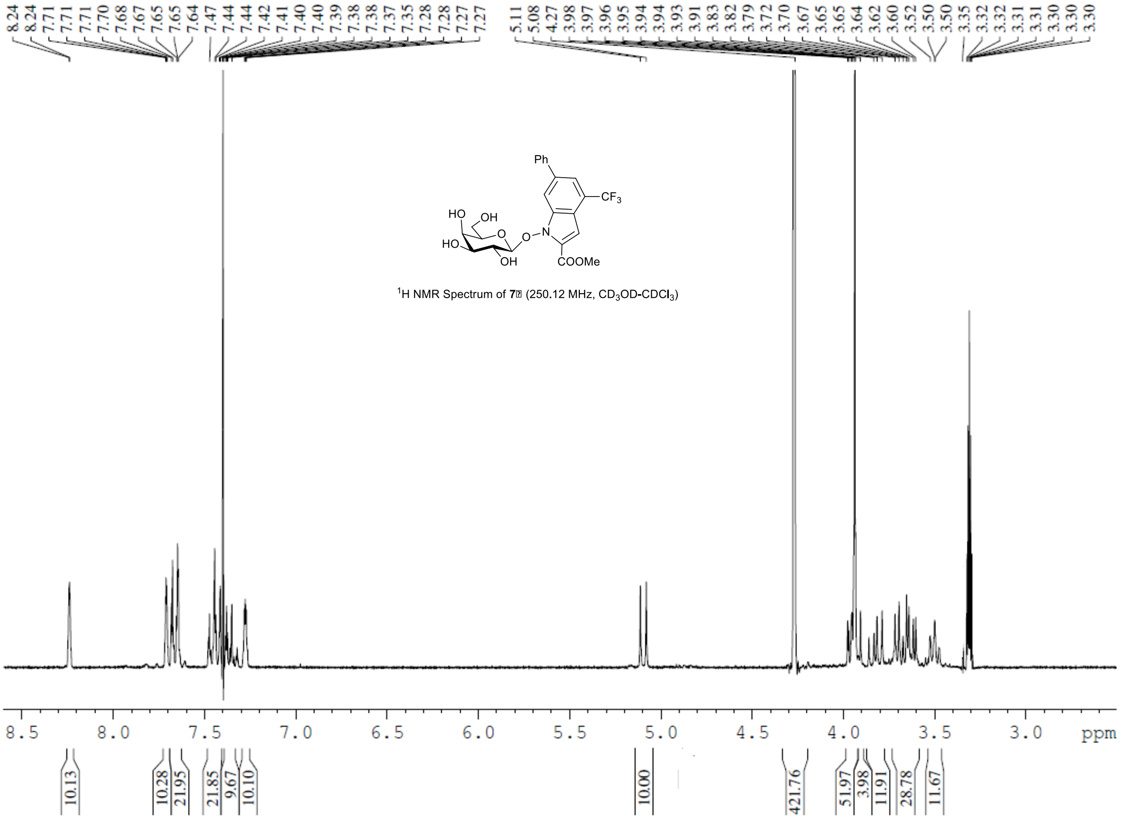

^{1}H NMR Spectrum of **7**[illegible] (250.12 MHz, CD_3OD-$CDCl_3$)

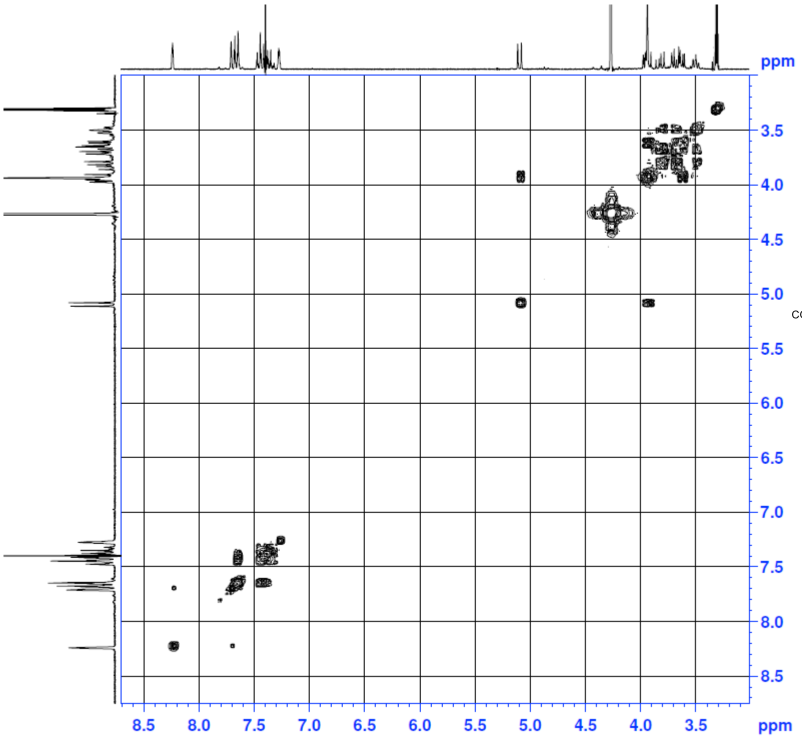

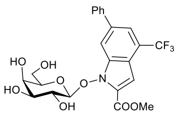

COSY Spectrum of **7** (250.12 MHz, CD_3OD-$CDCl_3$)

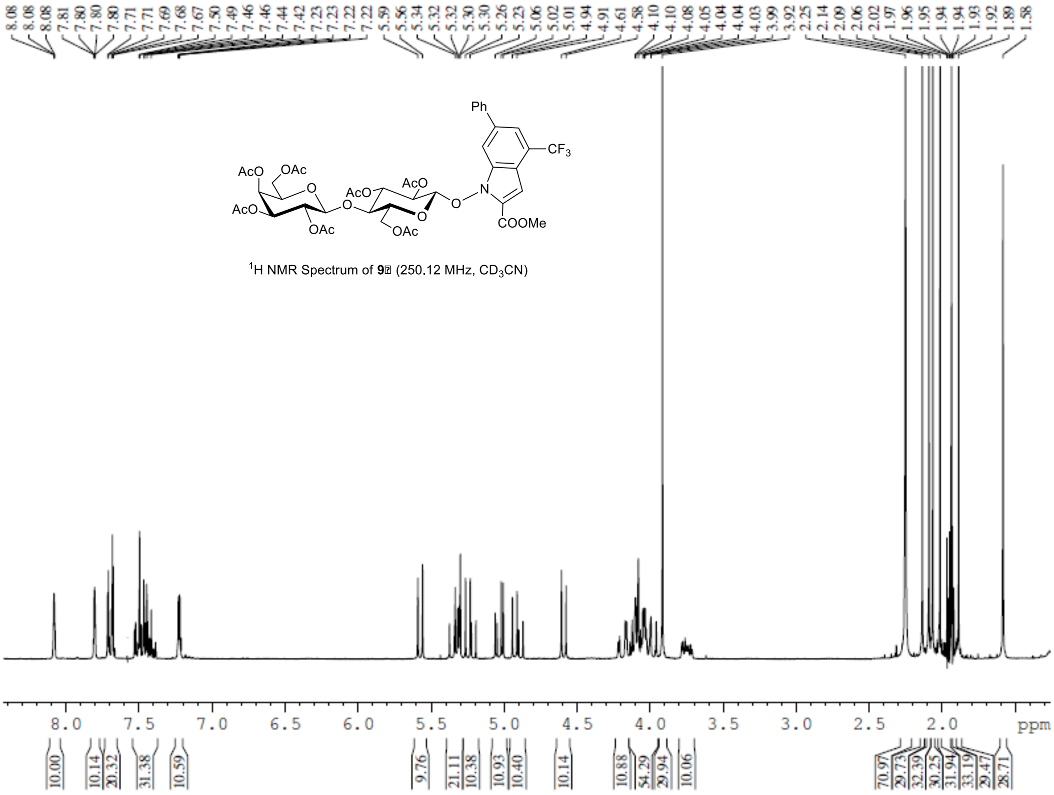

^{1}H NMR Spectrum of **9** (250.12 MHz, CD_3CN)

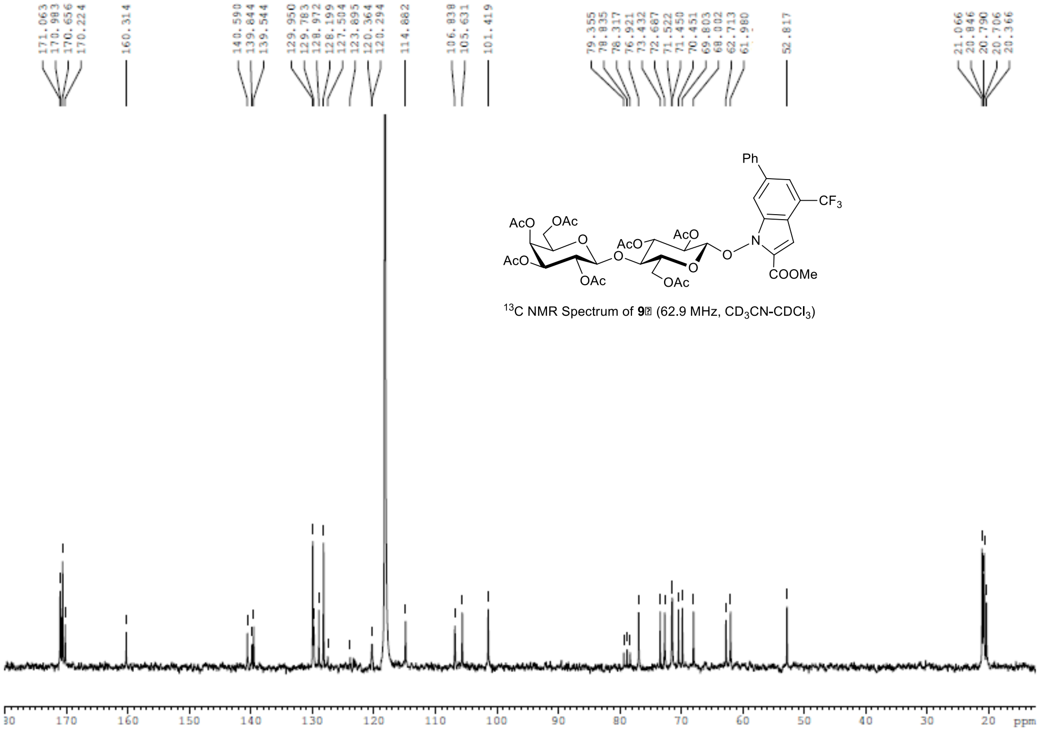

^{13}C NMR Spectrum of **9** (62.9 MHz, CD_3CN-$CDCl_3$)

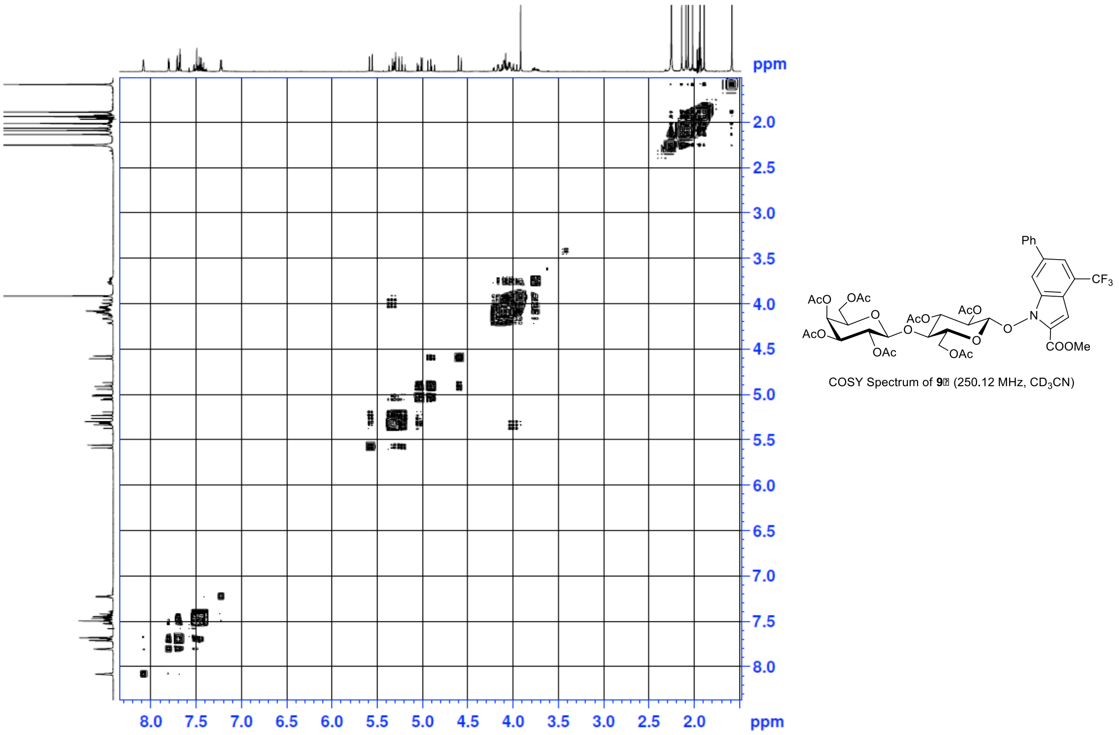

COSY Spectrum of **9** (250.12 MHz, CD_3CN)

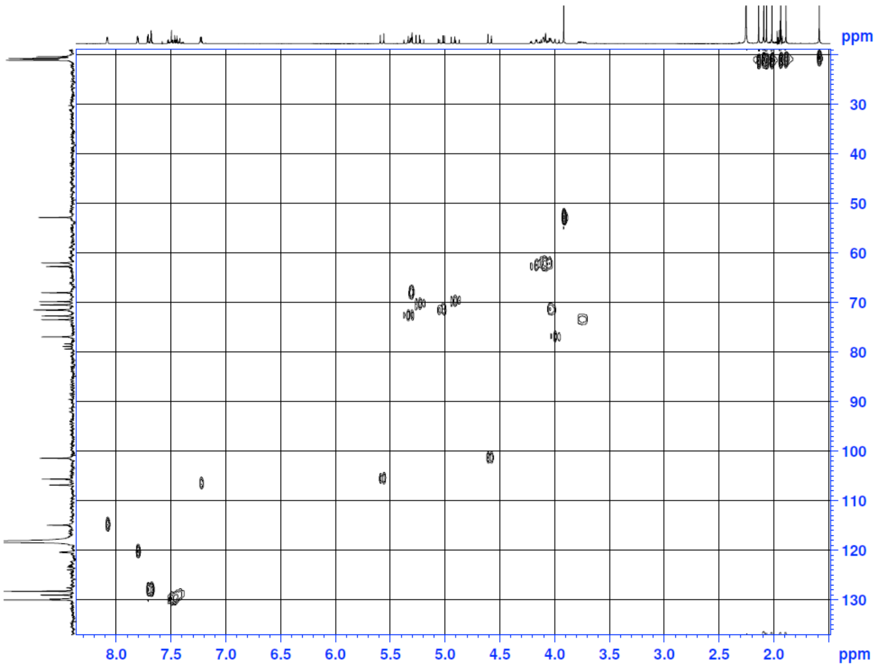

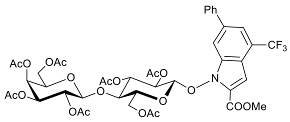

HSQC Spectrum of **9** (250.12 MHz, CD_3CN)

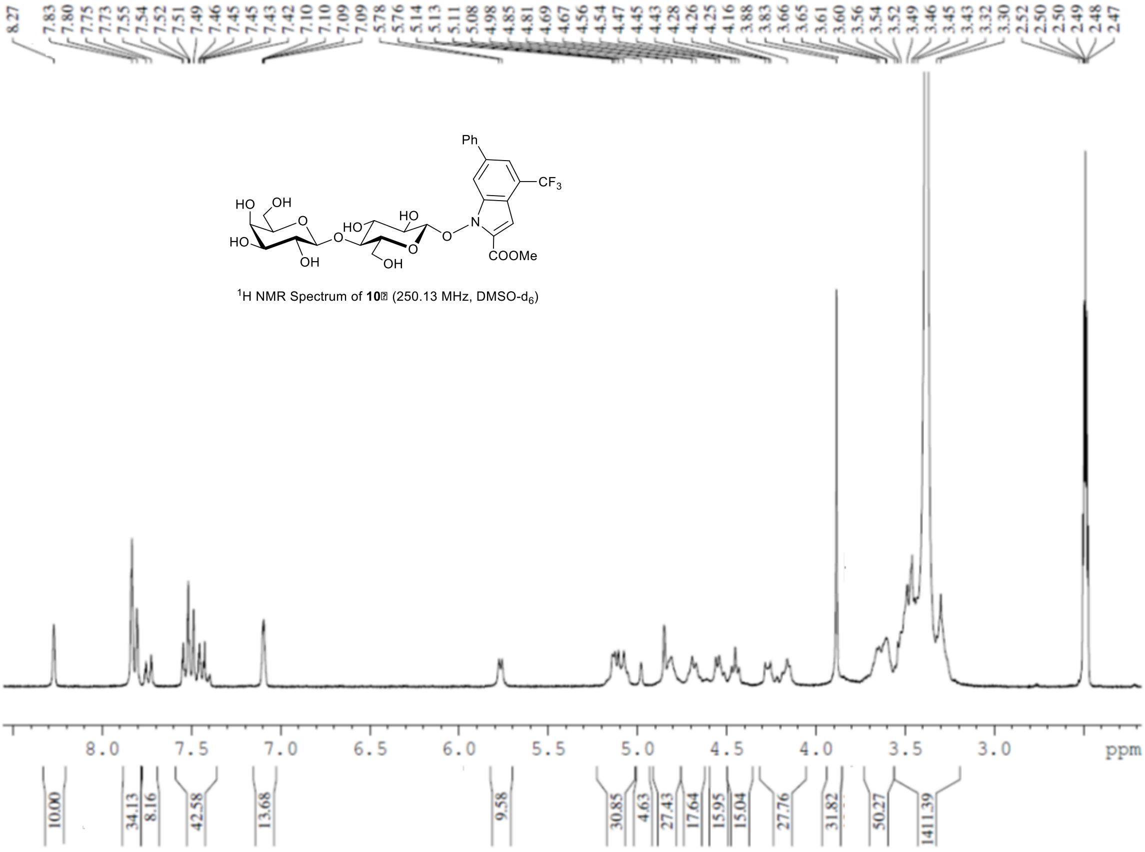

^1H NMR Spectrum of **10** (250.13 MHz, DMSO-d_6)

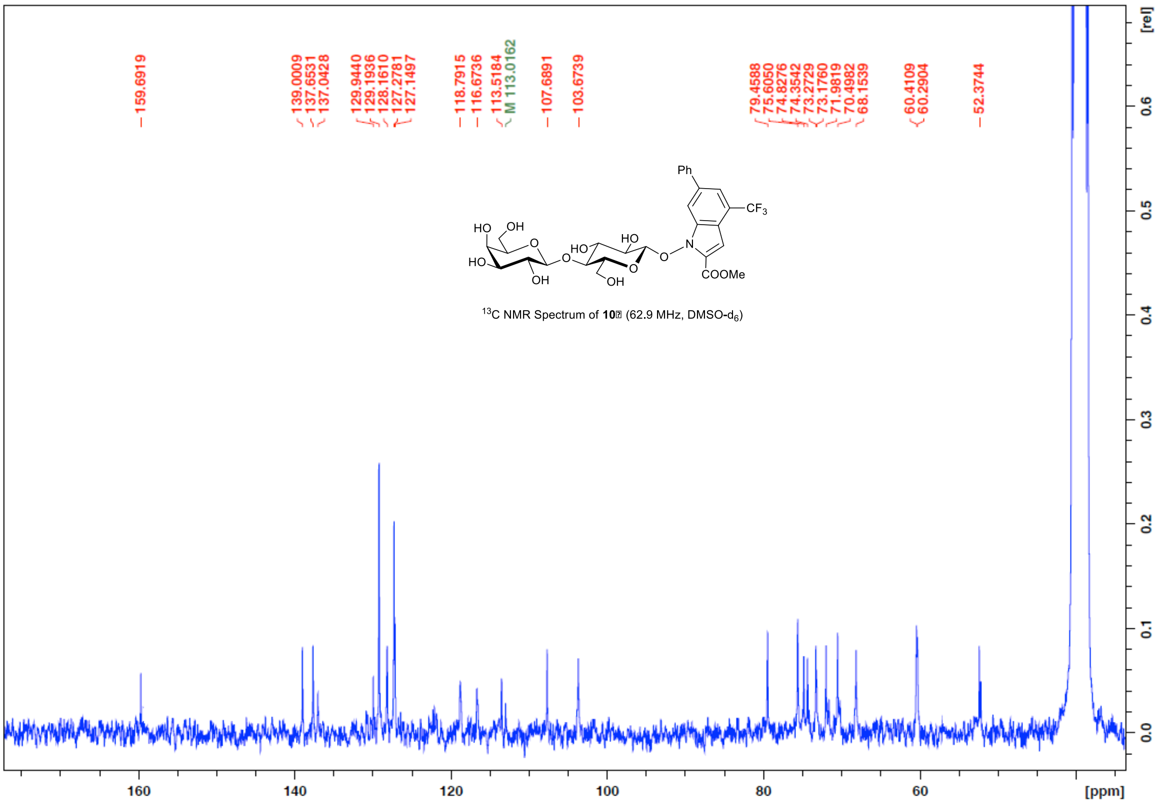

^{13}C NMR Spectrum of **10** (62.9 MHz, DMSO-d$_6$)

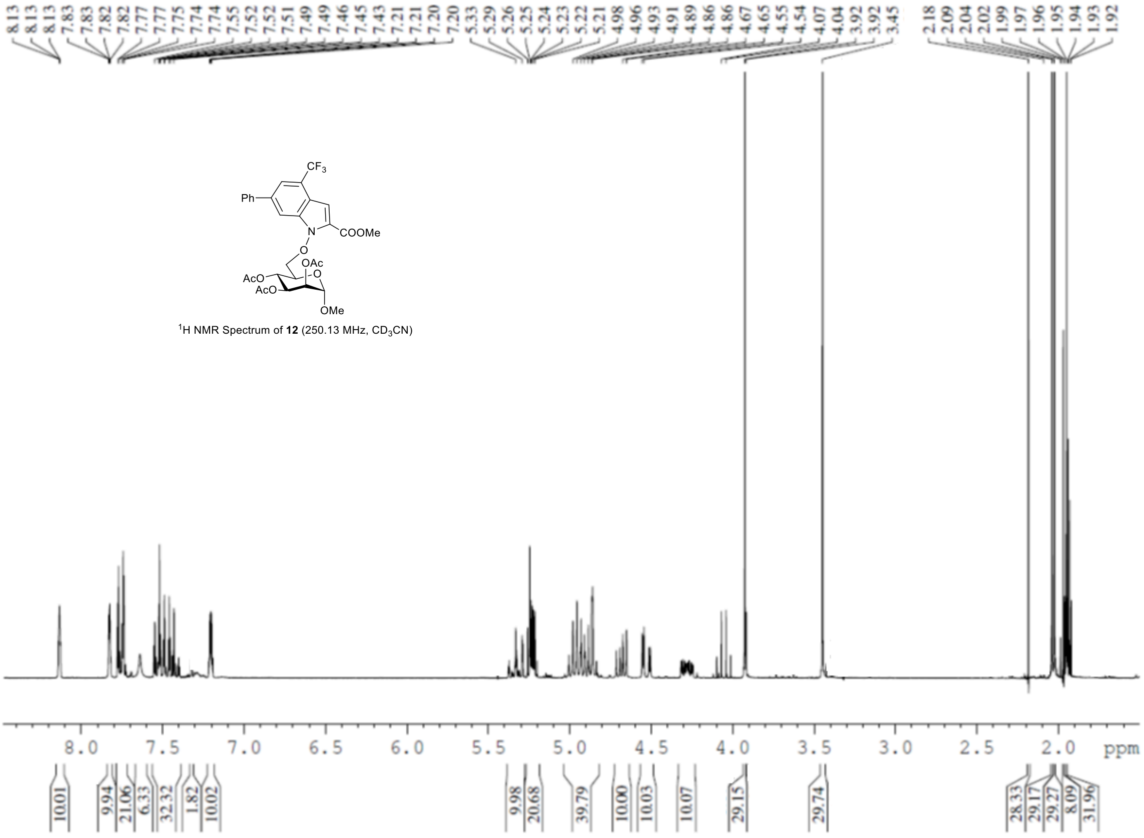

^{1}H NMR Spectrum of **12** (250.13 MHz, CD$_3$CN)

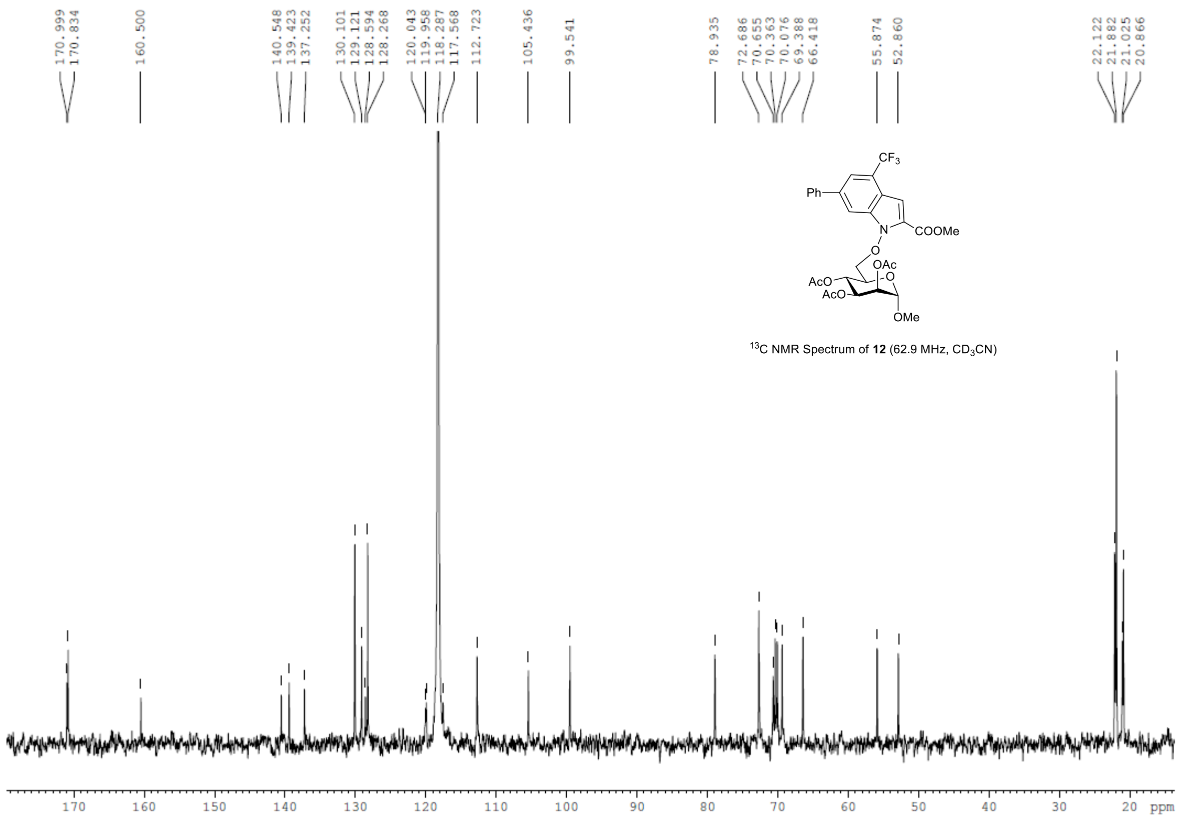

^{13}C NMR Spectrum of **12** (62.9 MHz, CD_3CN)

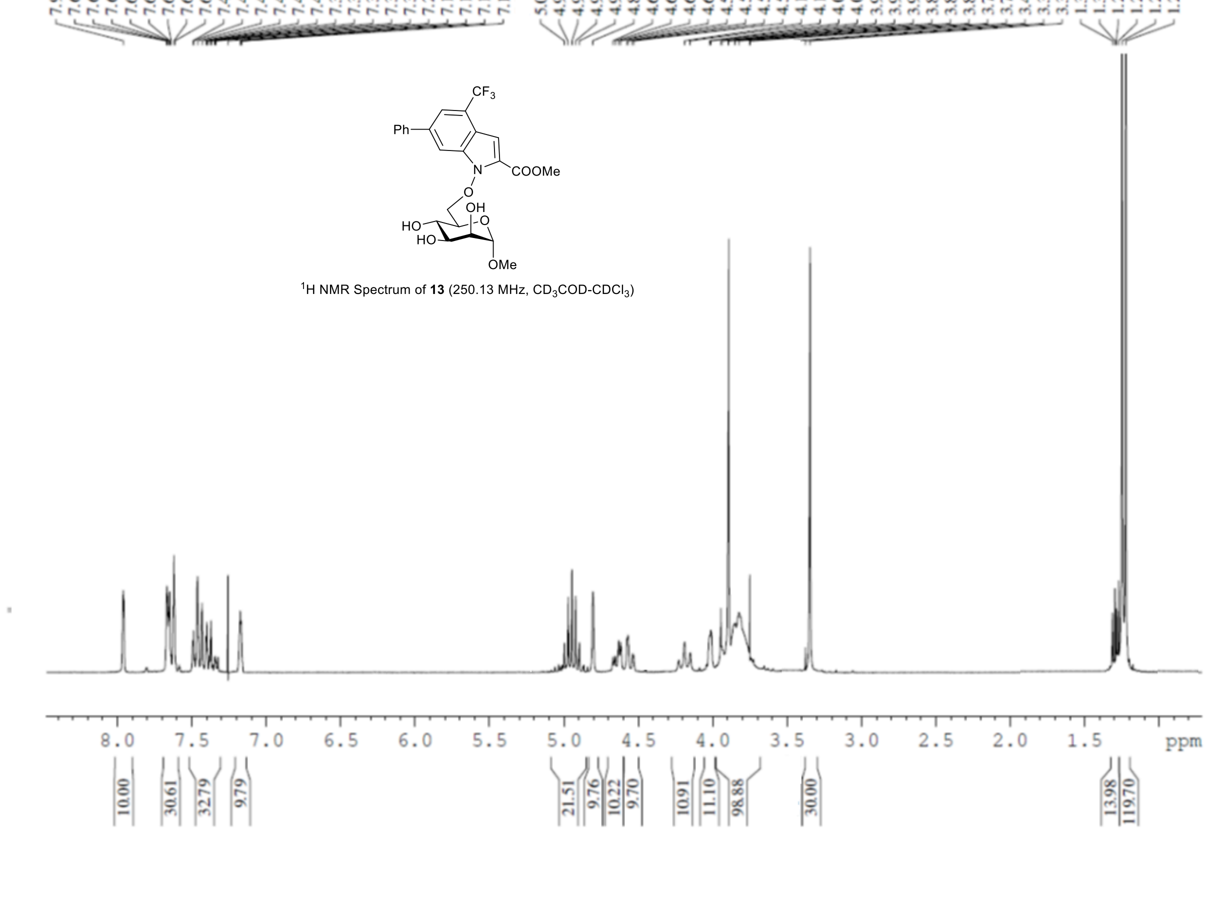

^{1}H NMR Spectrum of **13** (250.13 MHz, CD_3COD-$CDCl_3$)

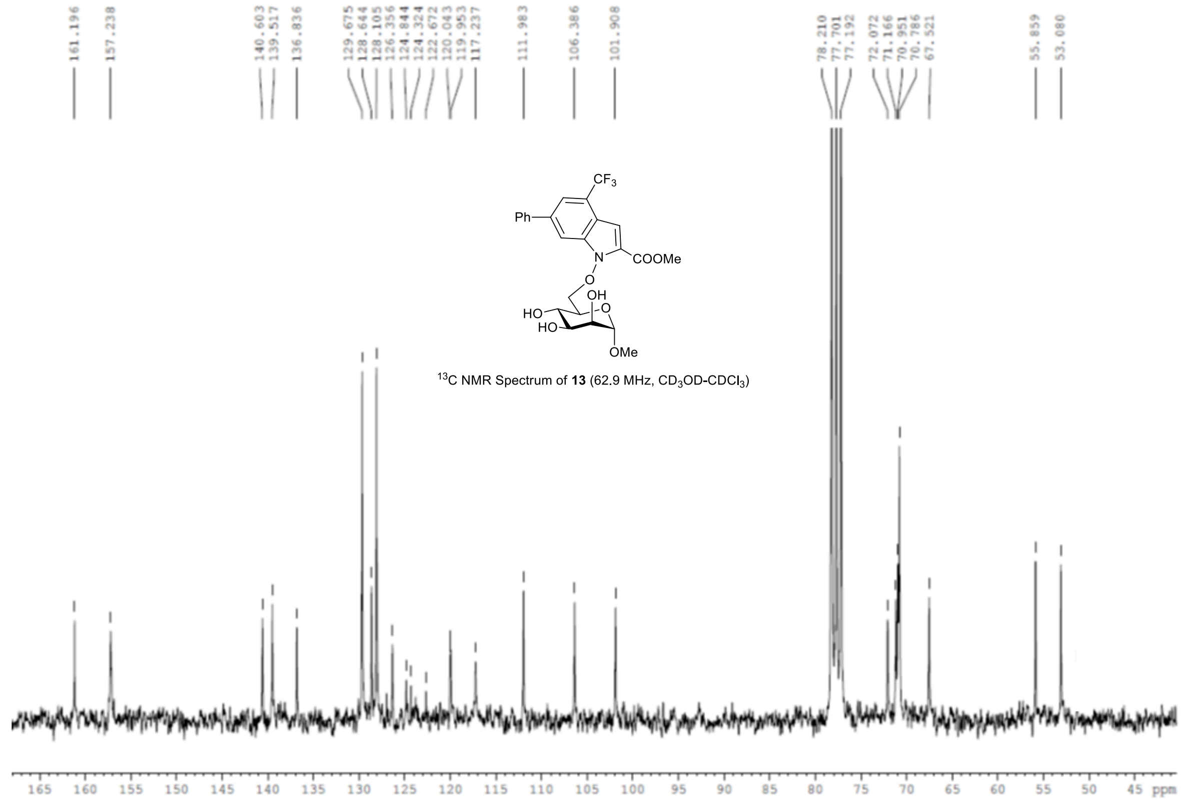

^{13}C NMR Spectrum of **13** (62.9 MHz, $CD_3OD-CDCl_3$)

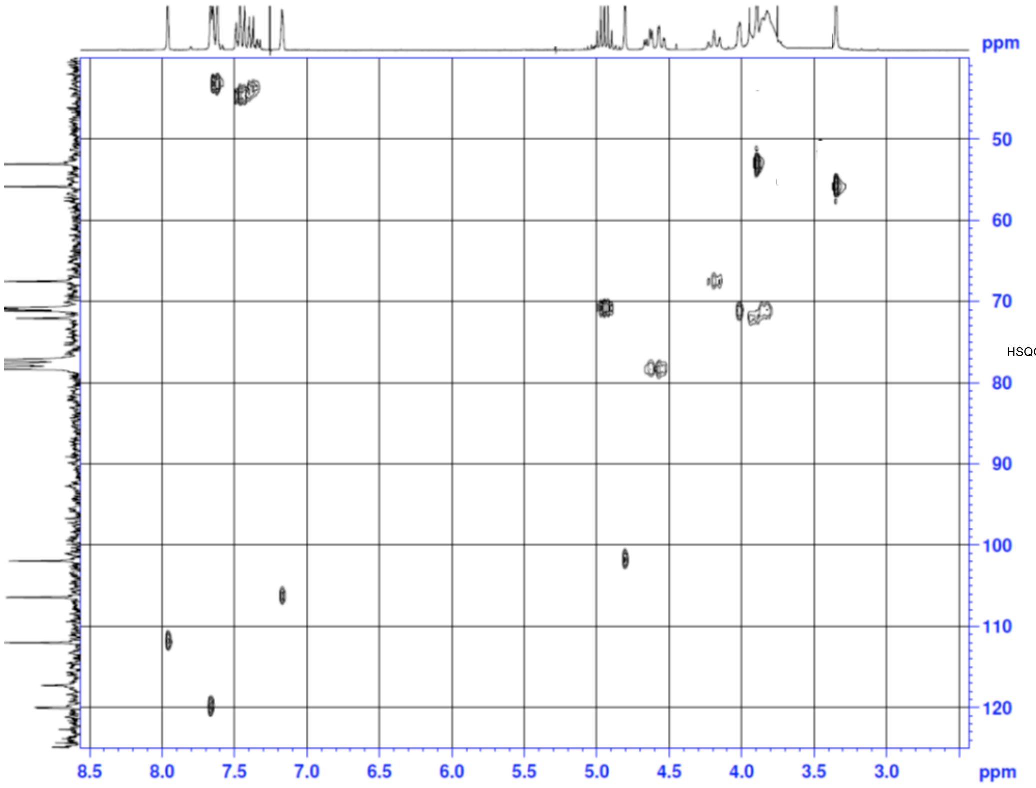

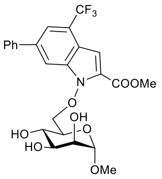

HSQC Spectrum of **13** (250.13 MHz, $CD_3COD-CDCl_3$)

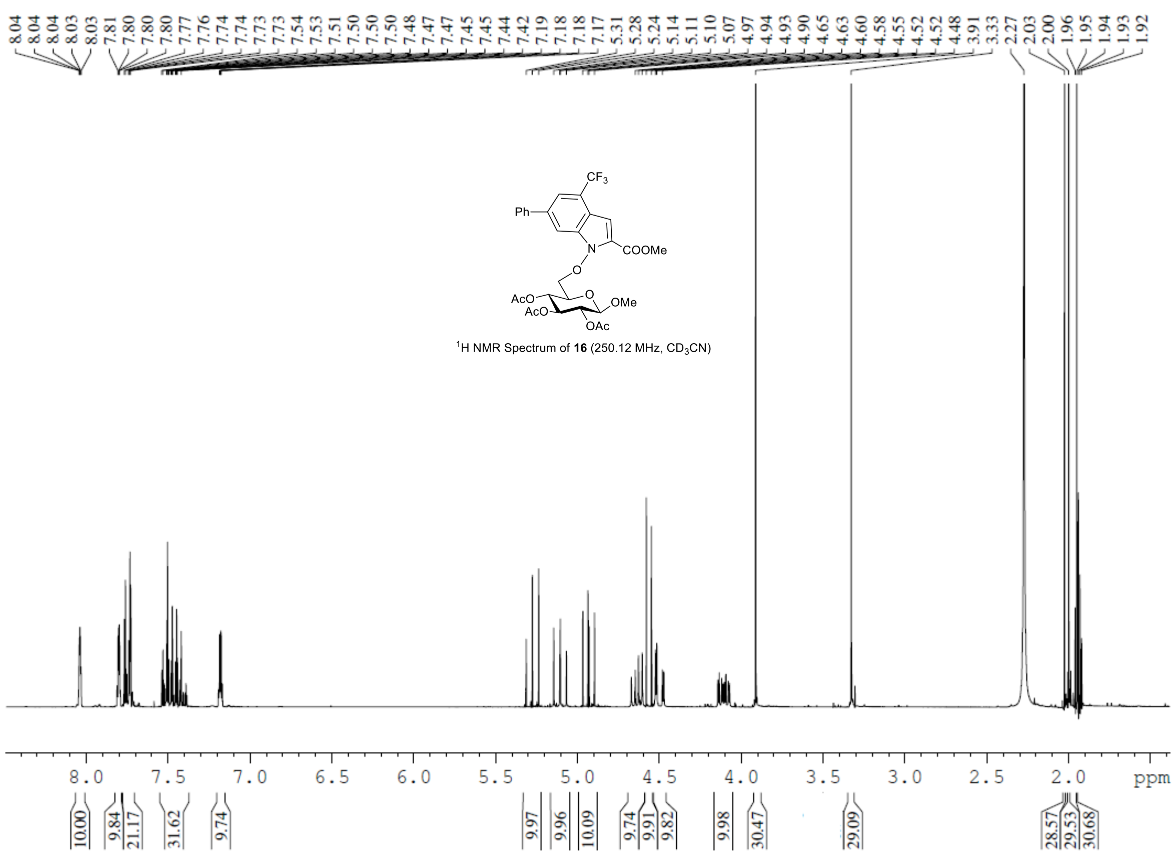

^{1}H NMR Spectrum of **16** (250.12 MHz, CD_3CN)

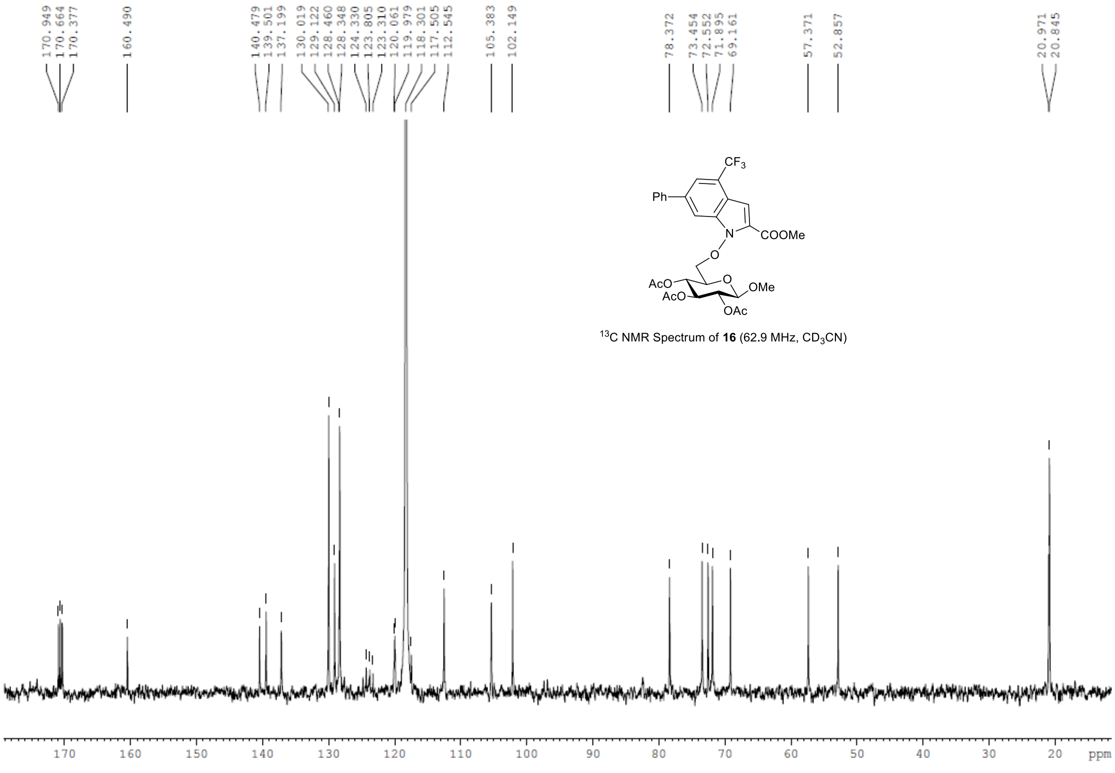

^{13}C NMR Spectrum of **16** (62.9 MHz, CD_3CN)

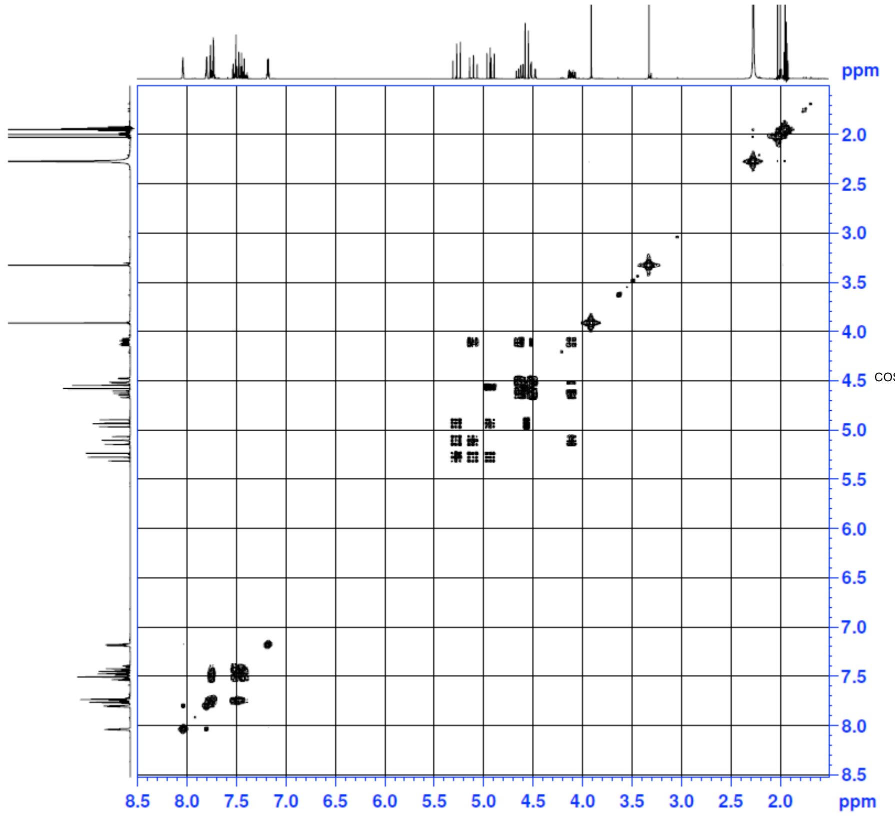

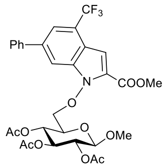

COSY Spectrum of **16** (250.12 MHz, CD_3CN)

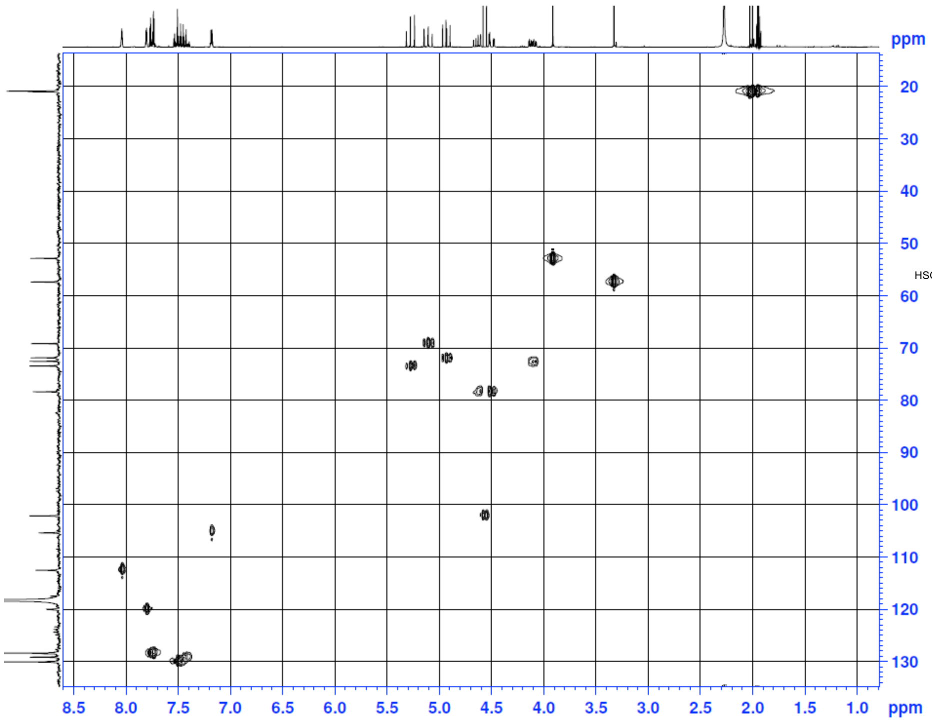

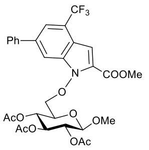

HSQC Spectrum of **16** (250.12 MHz, CD_3CN)

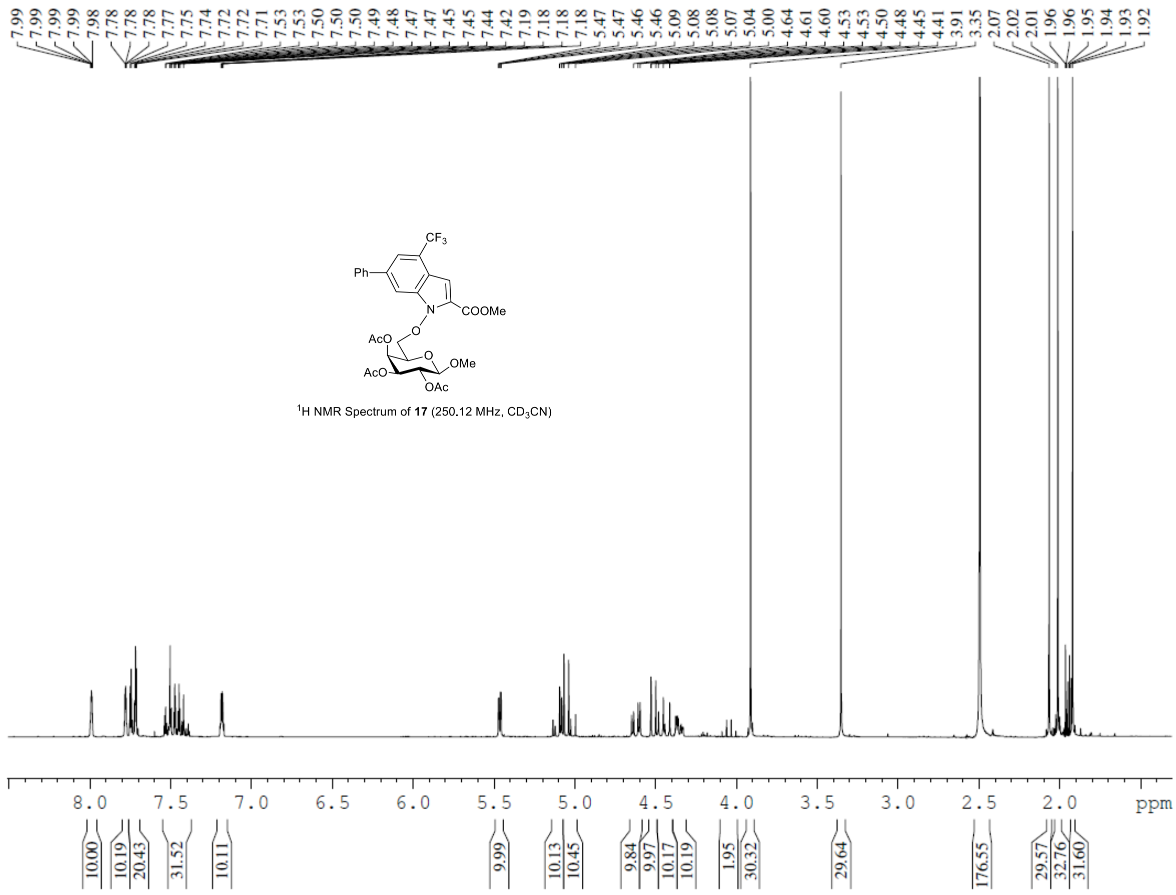

^{1}H NMR Spectrum of **17** (250.12 MHz, CD_3CN)

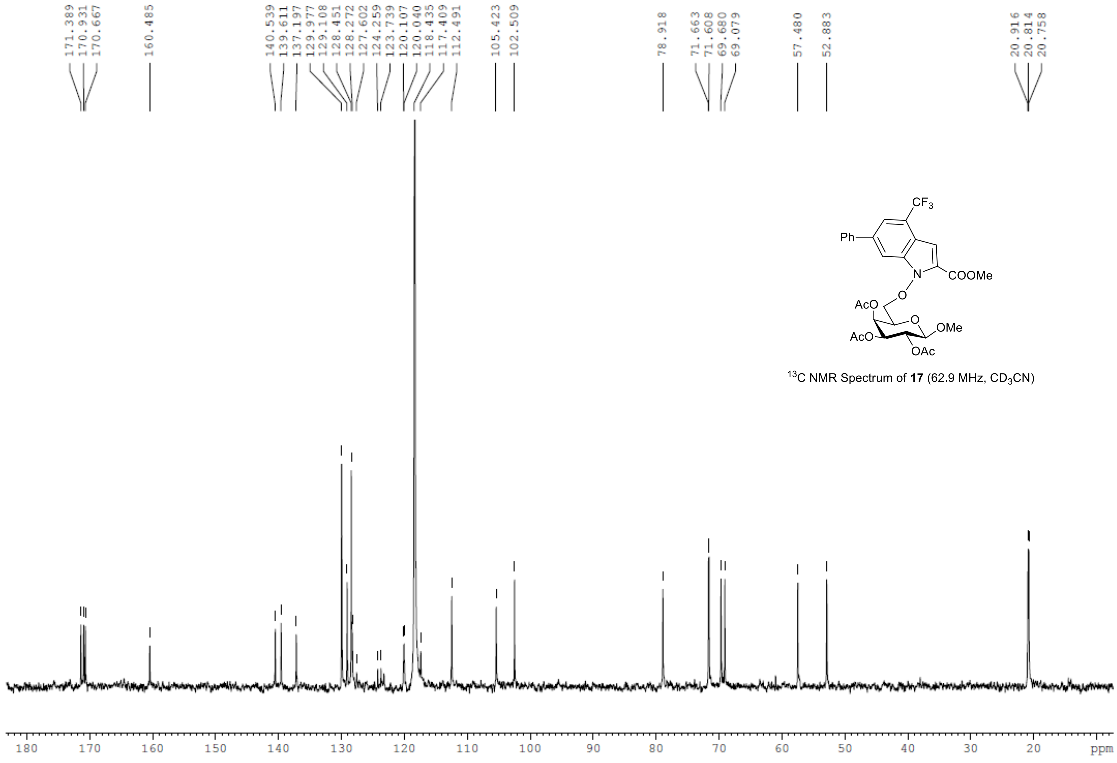

^{13}C NMR Spectrum of **17** (62.9 MHz, CD_3CN)

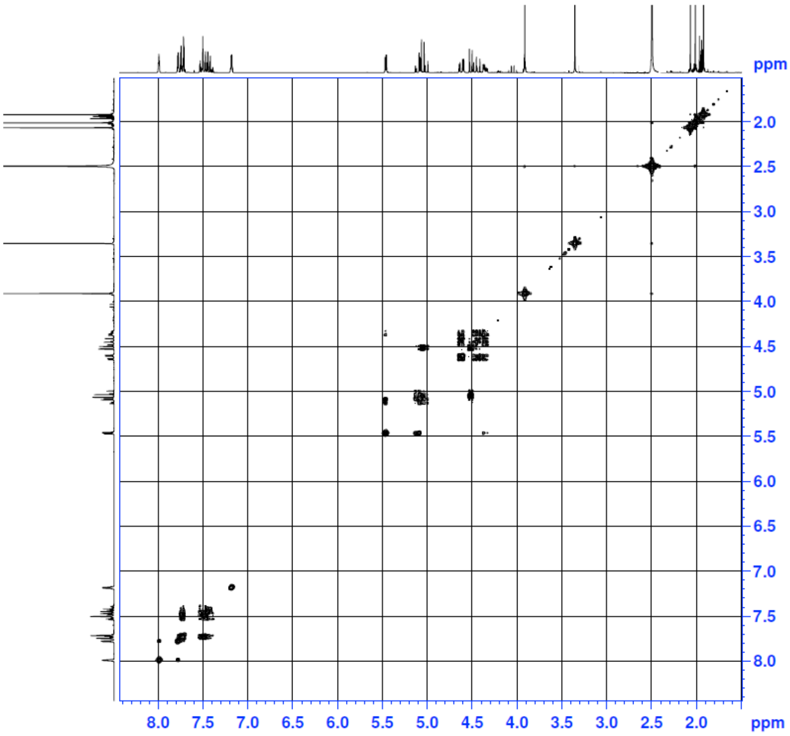

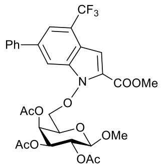

COSY Spectrum of **17** (250.12 MHz, CD_3CN)

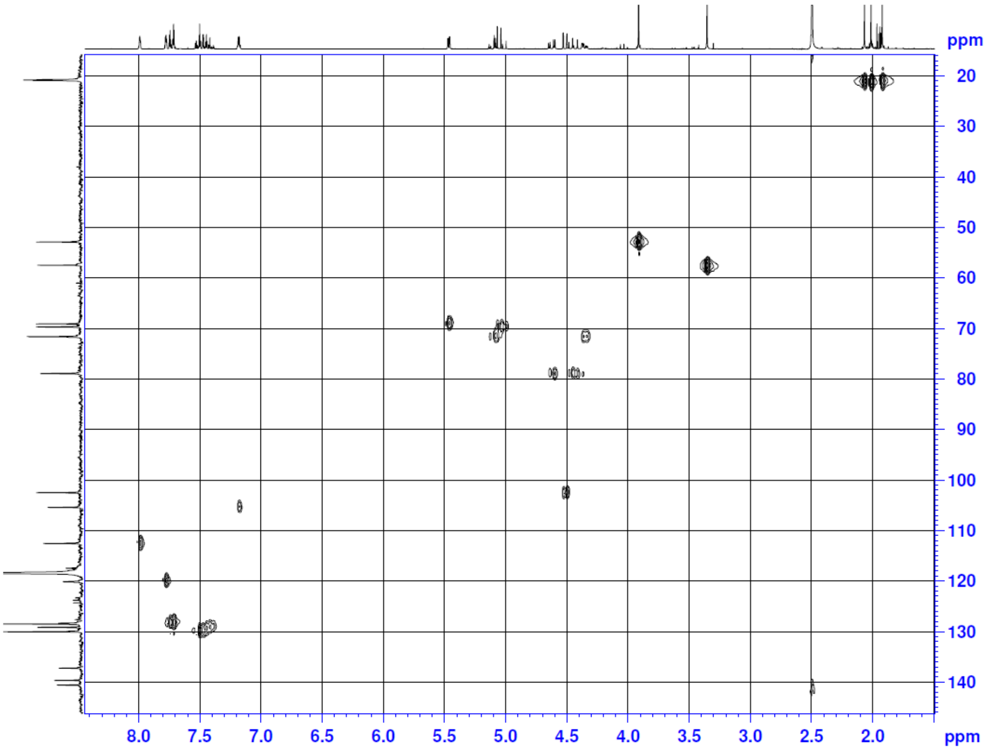

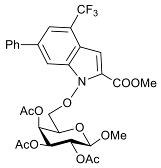

HSQC Spectrum of **17** (250.12 MHz, CD_3CN)

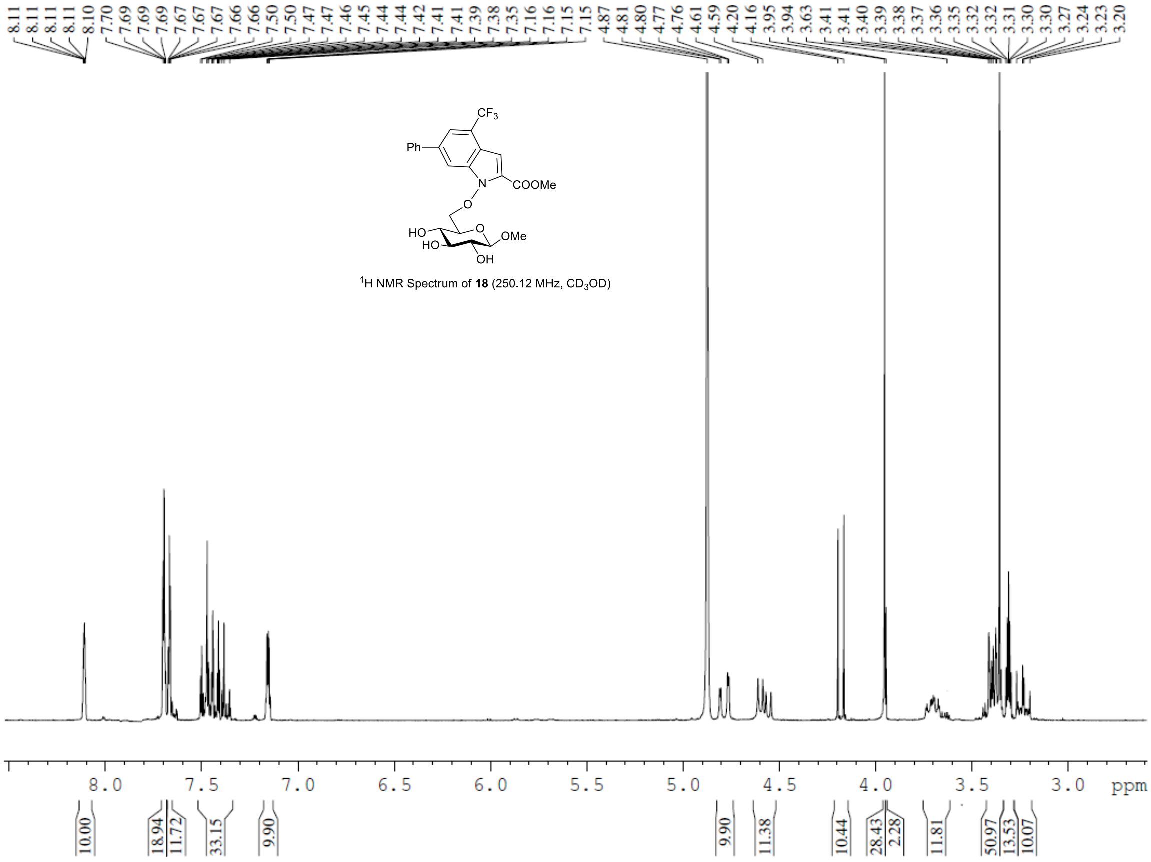

^{1}H NMR Spectrum of **18** (250.12 MHz, CD_3OD)

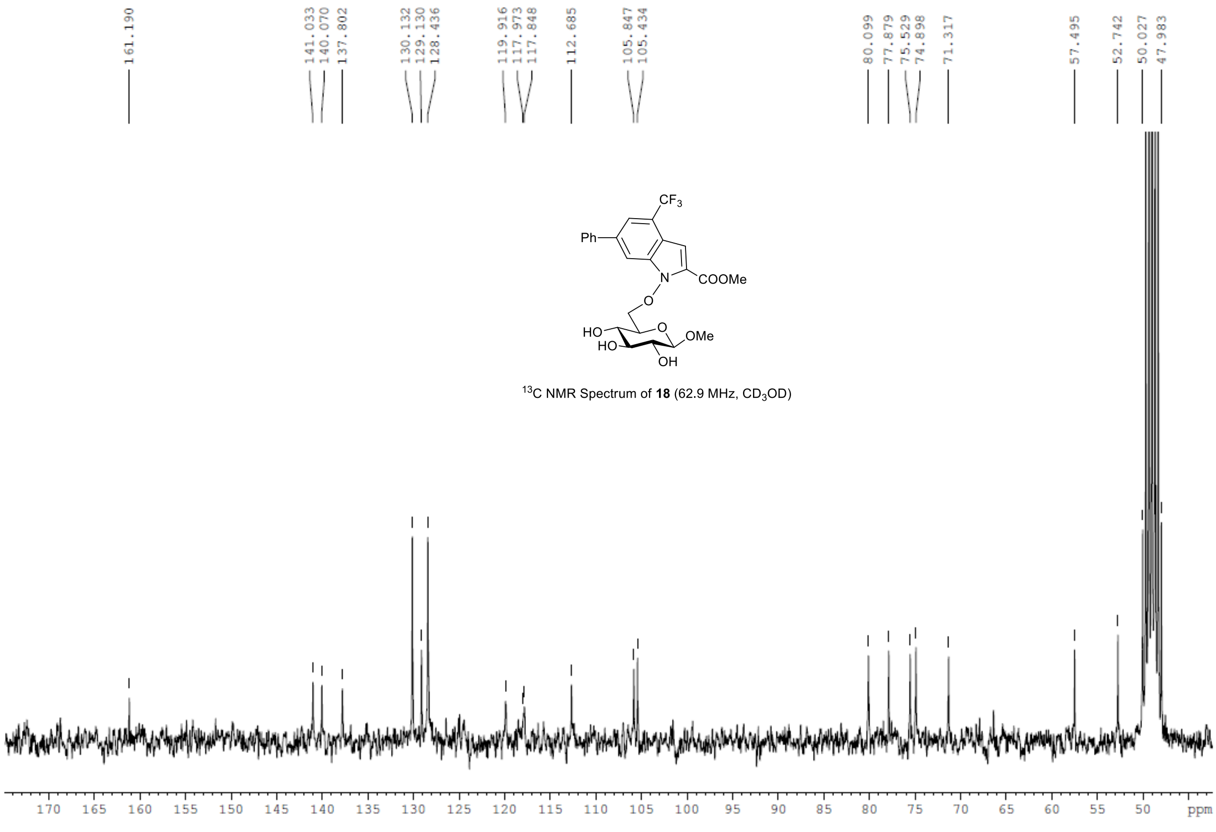

^{13}C NMR Spectrum of **18** (62.9 MHz, CD_3OD)

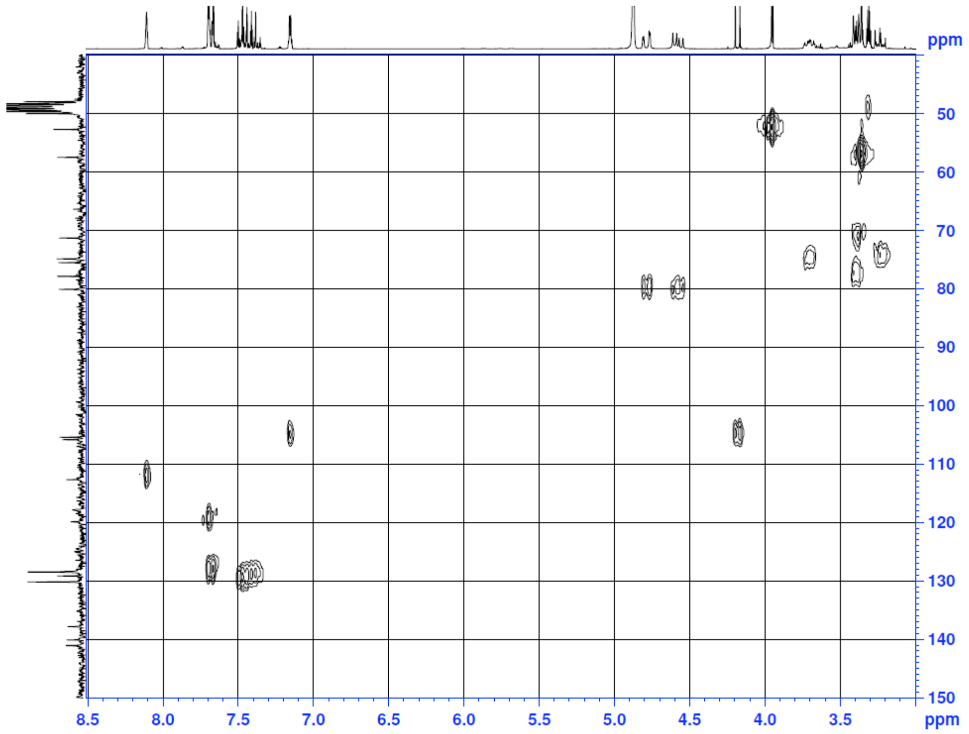

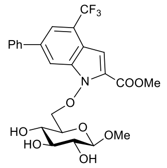

HSQC Spectrum of **18** (250.12 MHz, CD_3OD)

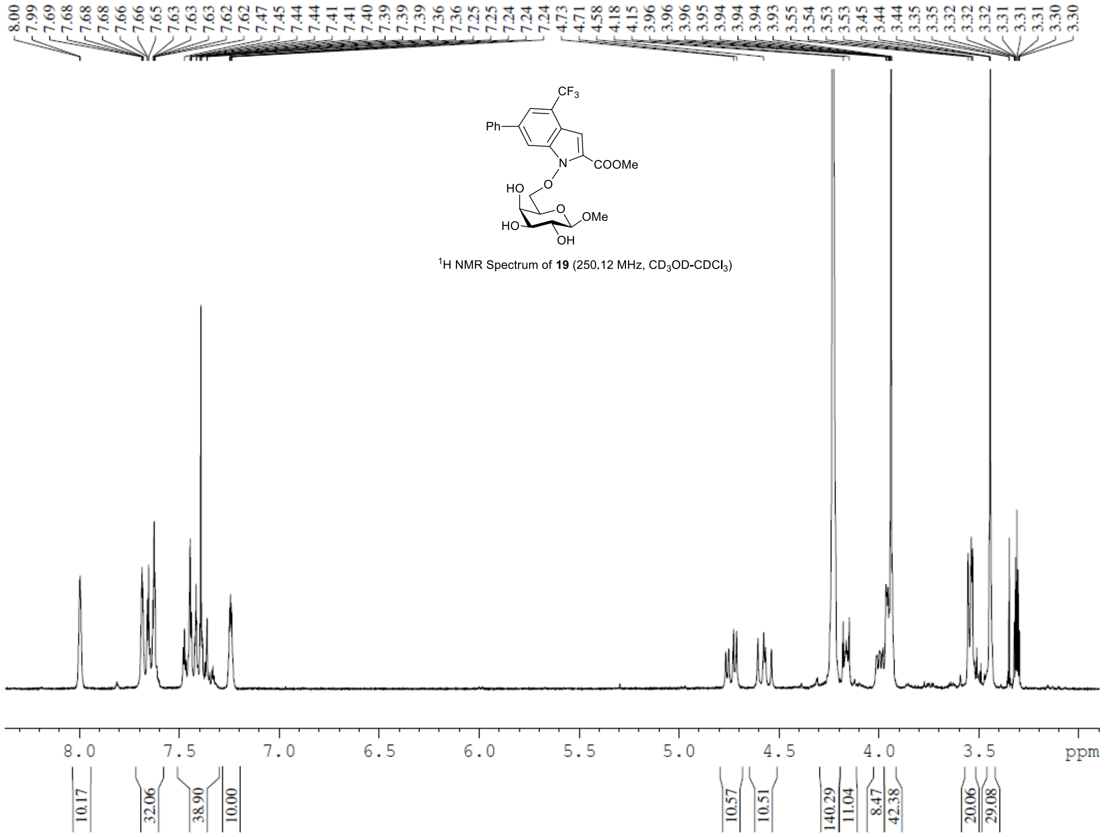

^{1}H NMR Spectrum of **19** (250.12 MHz, CD_3OD-$CDCl_3$)

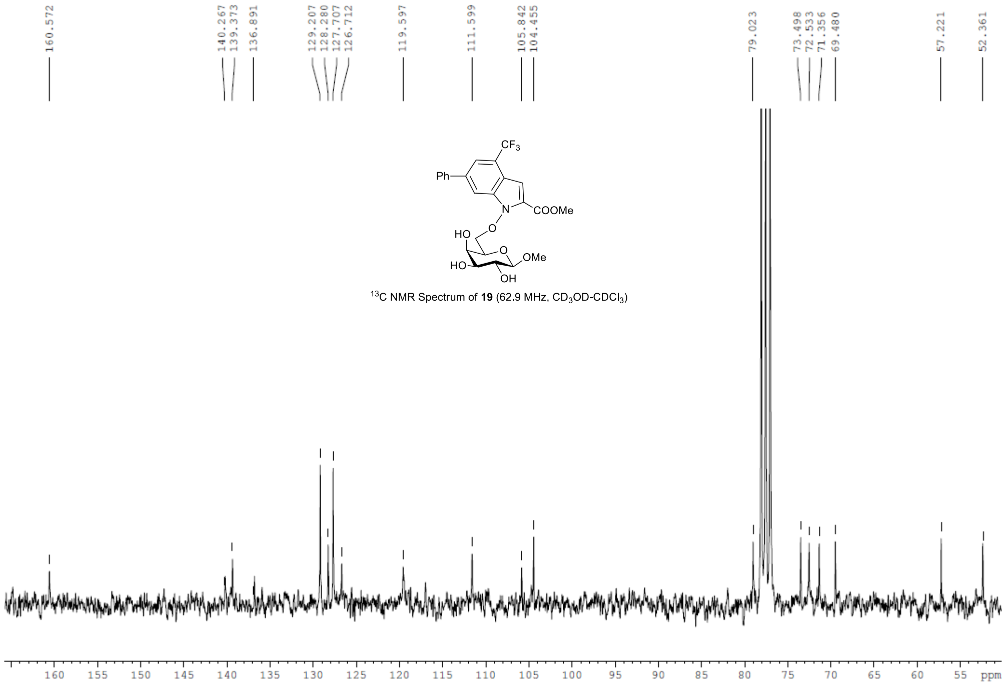

^{13}C NMR Spectrum of **19** (62.9 MHz, CD_3OD-$CDCl_3$)

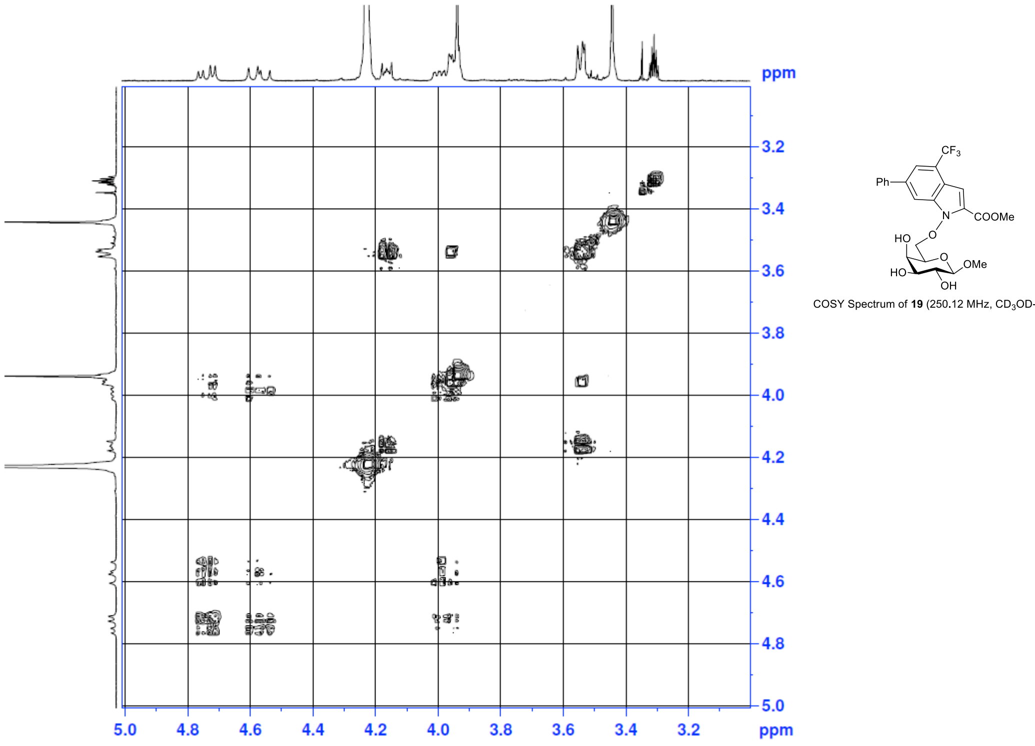

COSY Spectrum of **19** (250.12 MHz, CD_3OD-$CDCl_3$)

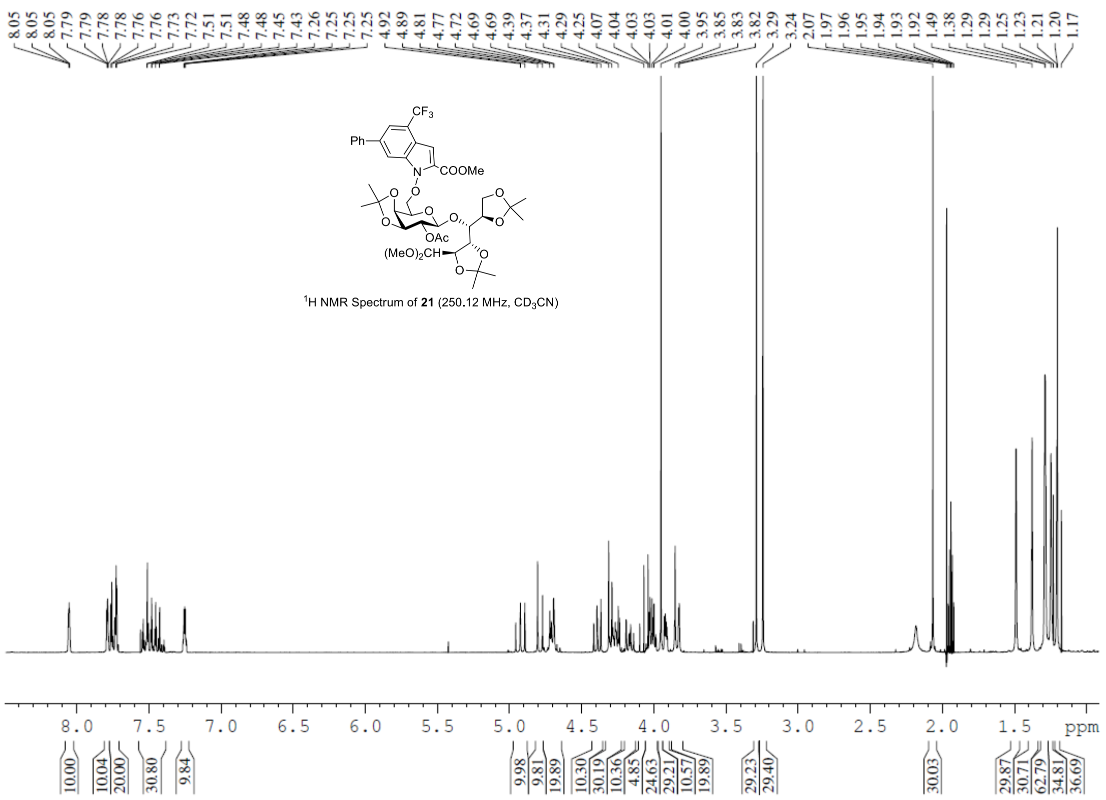

^{1}H NMR Spectrum of **21** (250.12 MHz, CD_3CN)

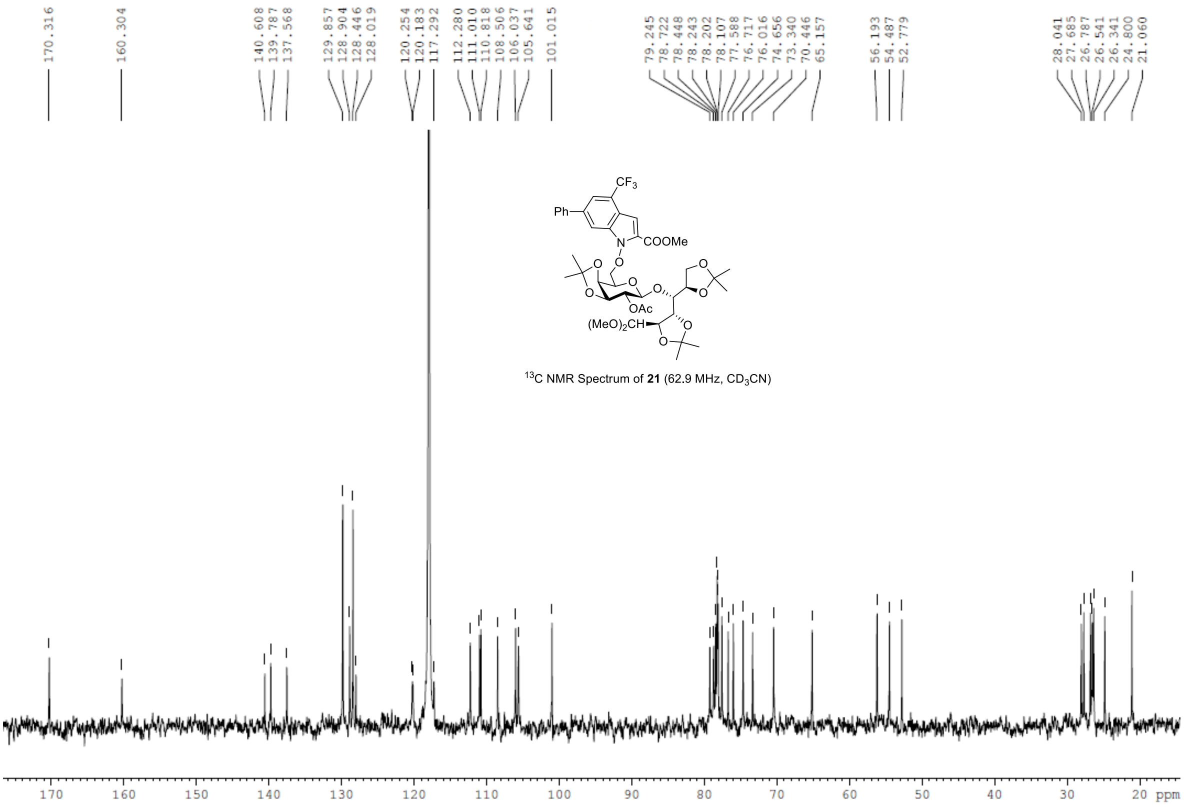

^{13}C NMR Spectrum of **21** (62.9 MHz, CD_3CN)

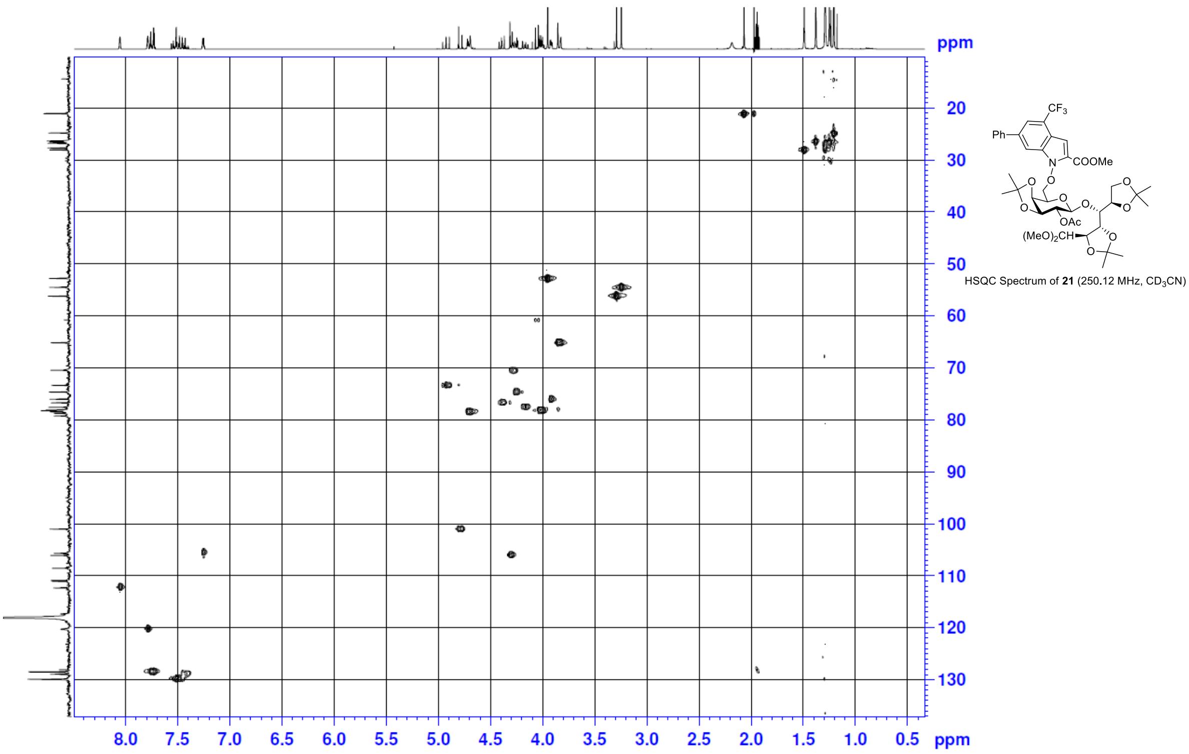

HSQC Spectrum of **21** (250.12 MHz, CD_3CN)

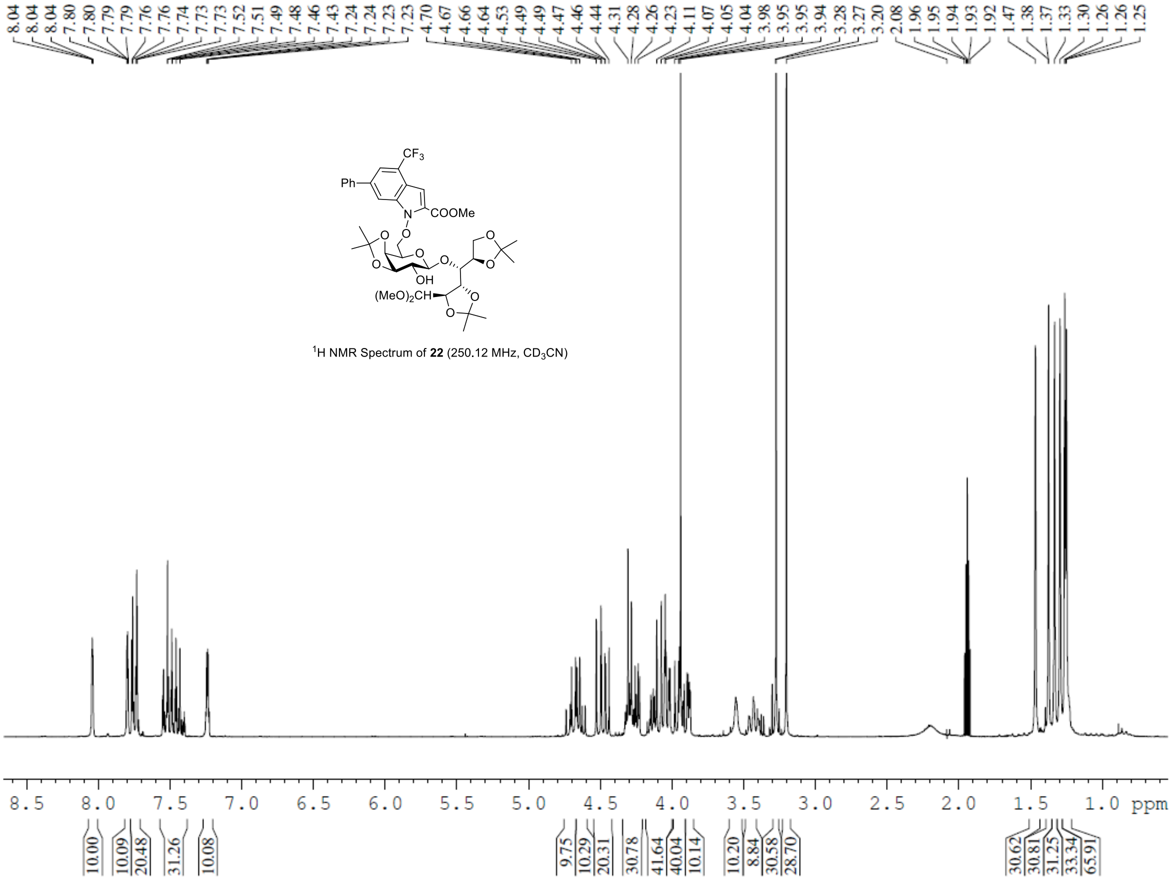

^{1}H NMR Spectrum of **22** (250.12 MHz, CD_3CN)

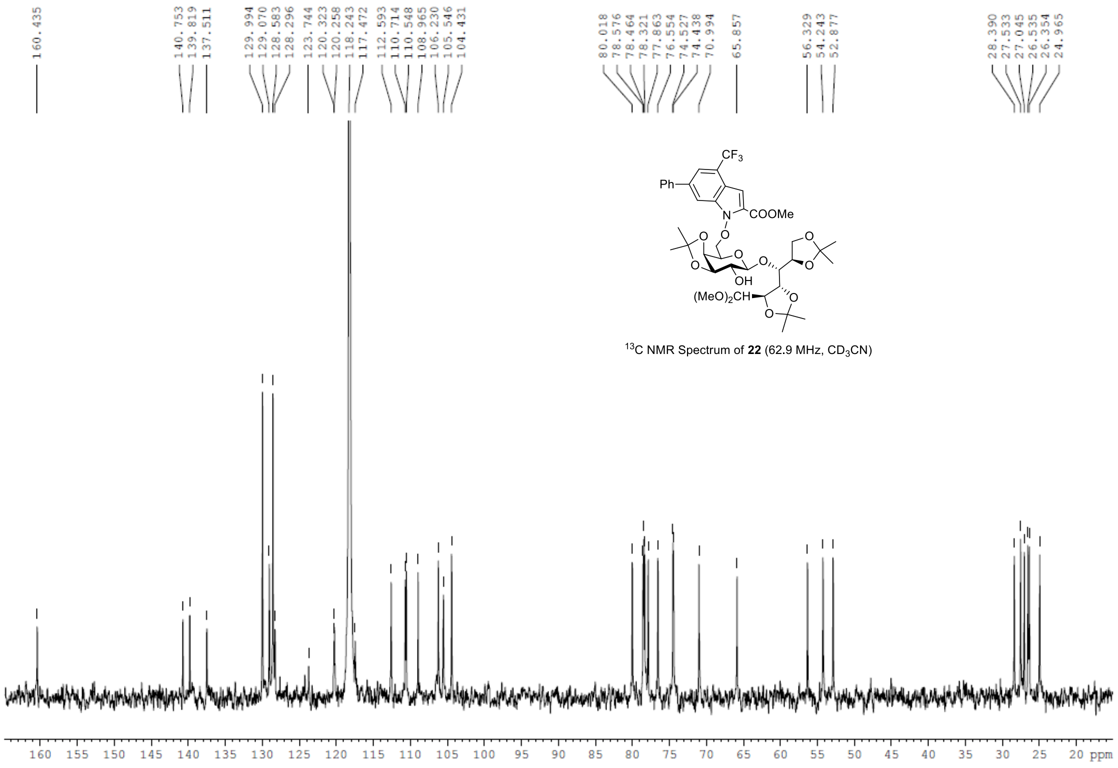

^{13}C NMR Spectrum of **22** (62.9 MHz, CD_3CN)

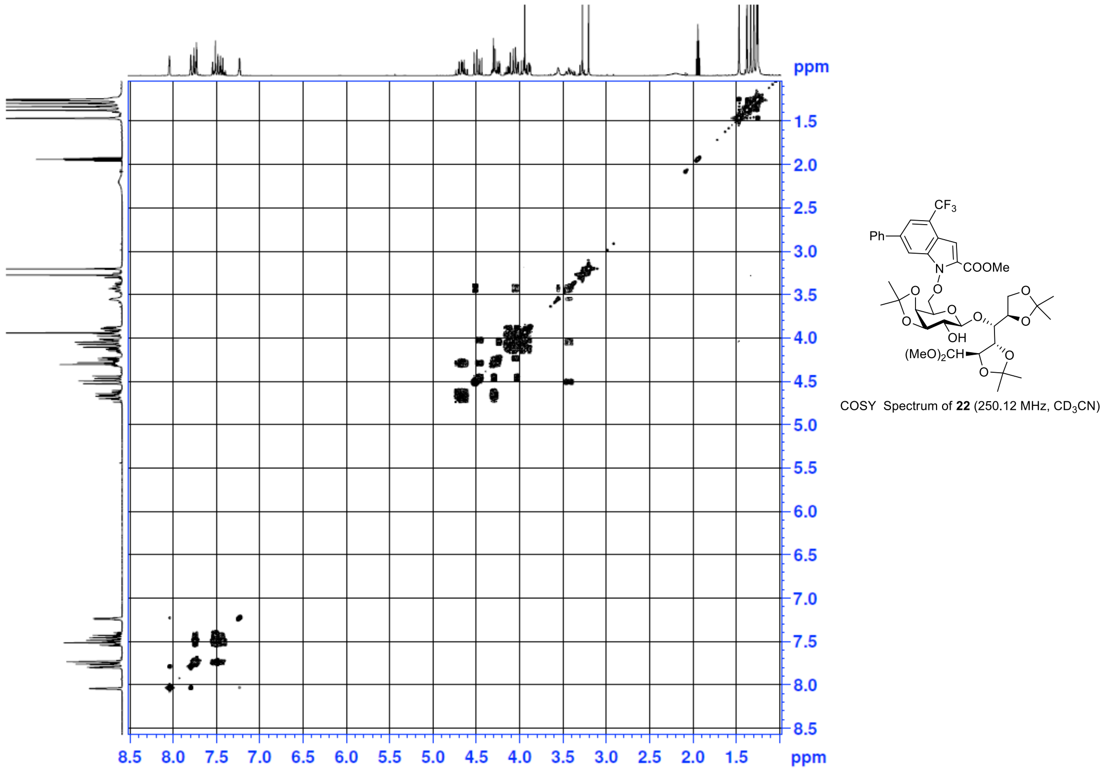

COSY Spectrum of **22** (250.12 MHz, CD_3CN)

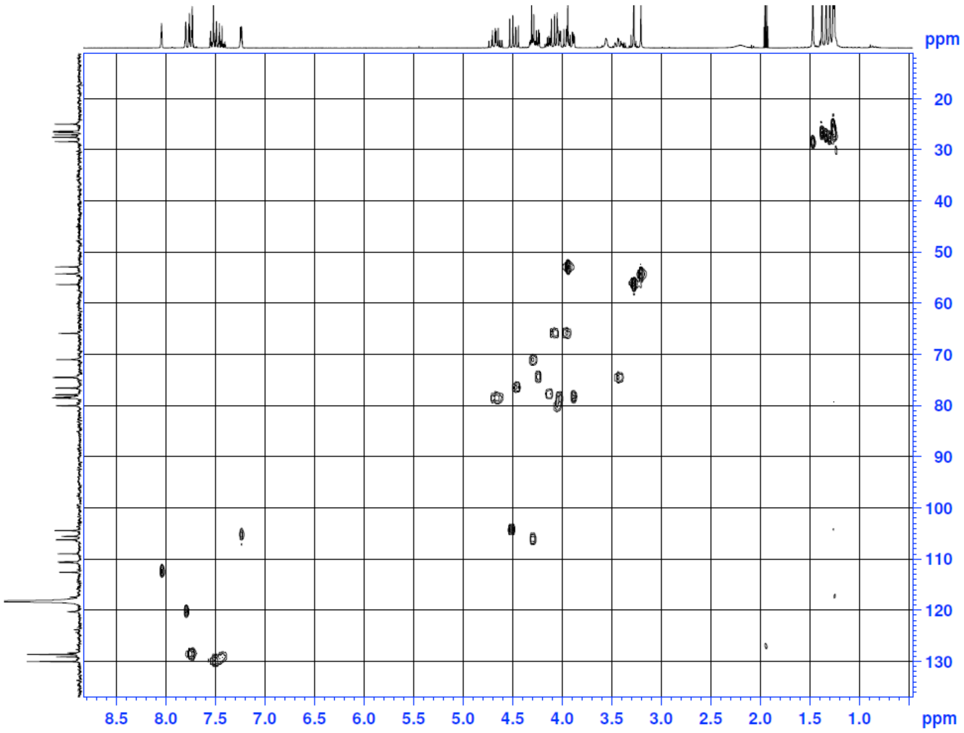

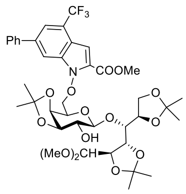

HSQC Spectrum of **22** (250.12 MHz, CD_3CN)

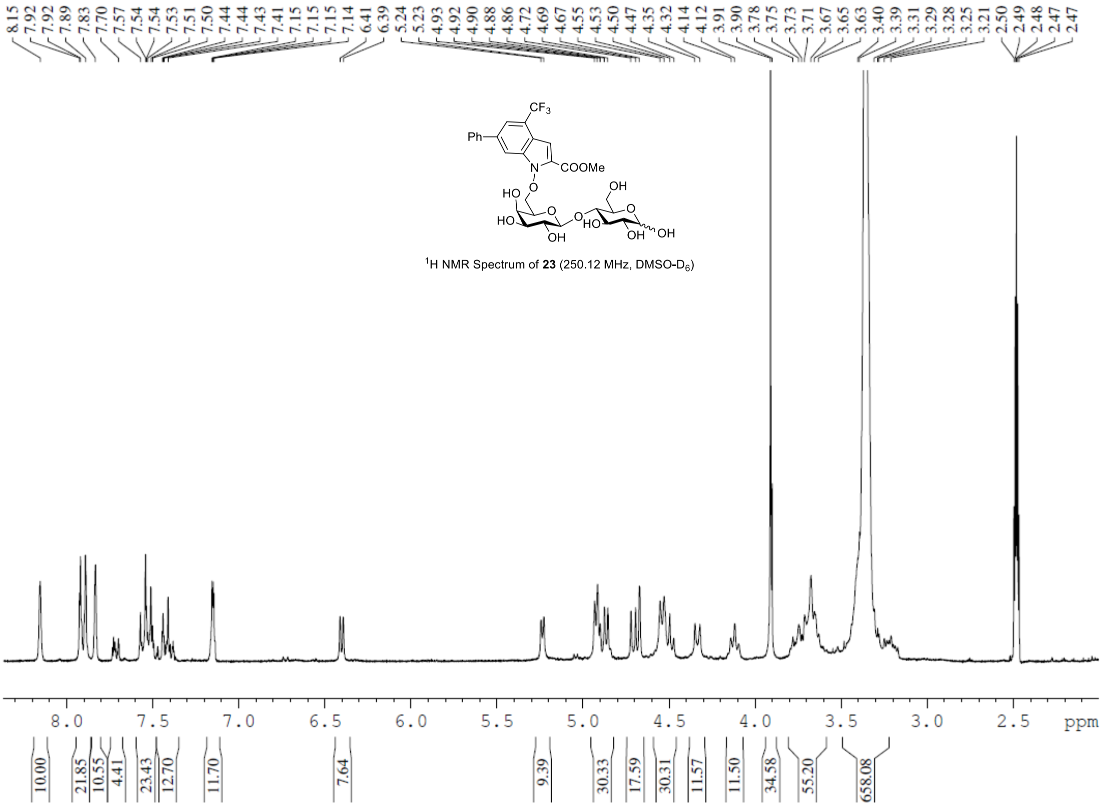

^{1}H NMR Spectrum of **23** (250.12 MHz, DMSO-D_6)

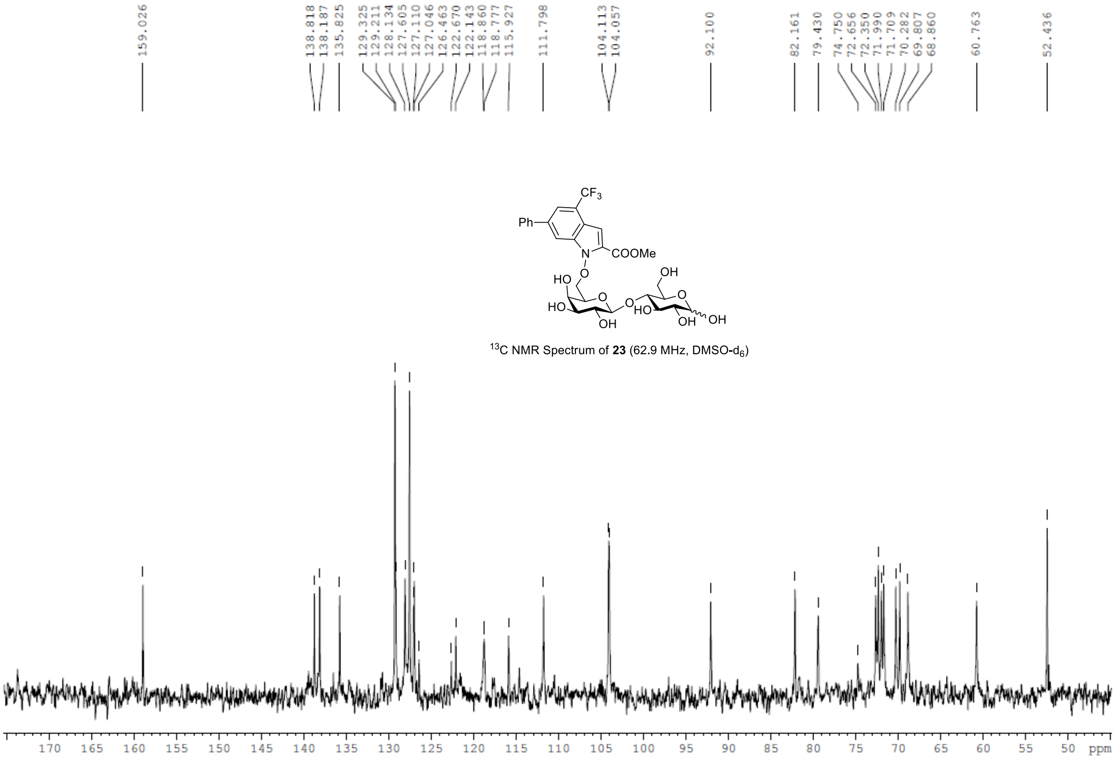

^{13}C NMR Spectrum of **23** (62.9 MHz, DMSO-d_6)

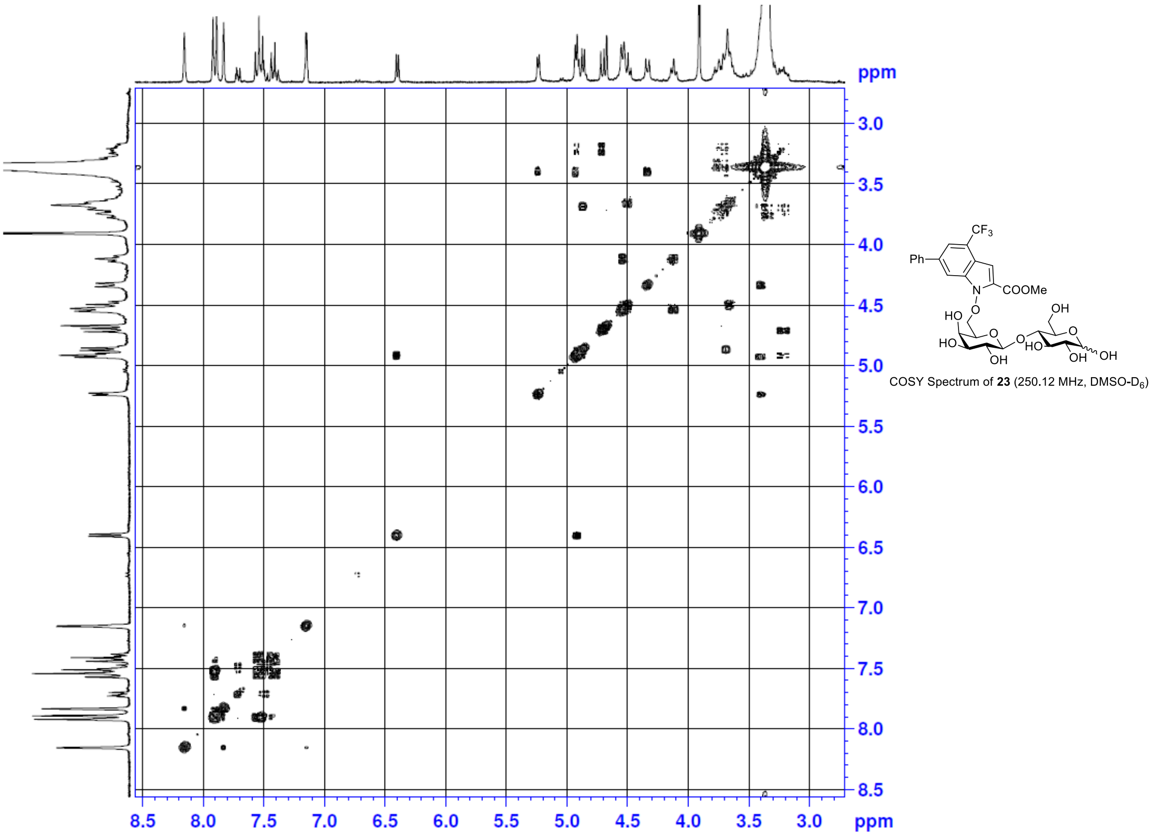

COSY Spectrum of **23** (250.12 MHz, DMSO-D_6)

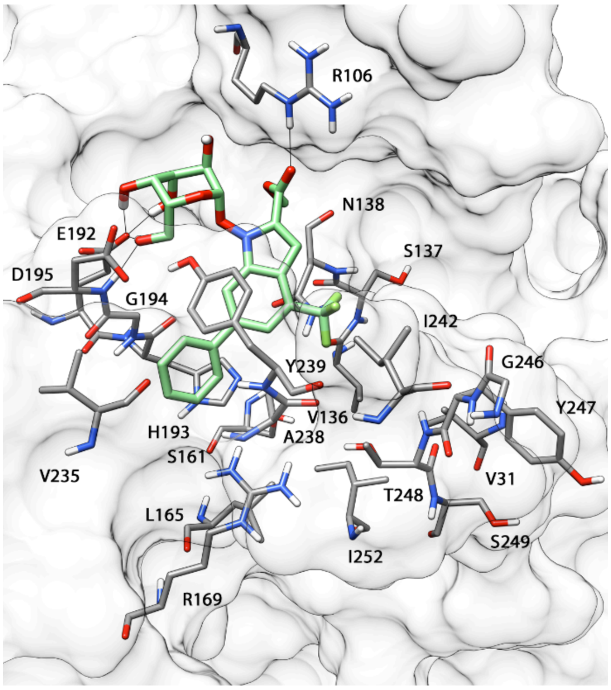

Figure S1. Binding disposition of **7β** into *h*LDH5.

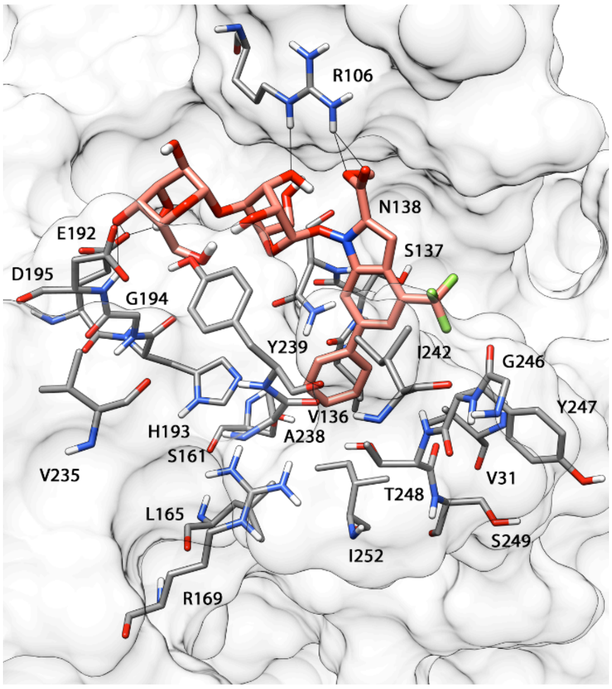

Figure S2. Binding disposition of **10β** into *h*LDH5.

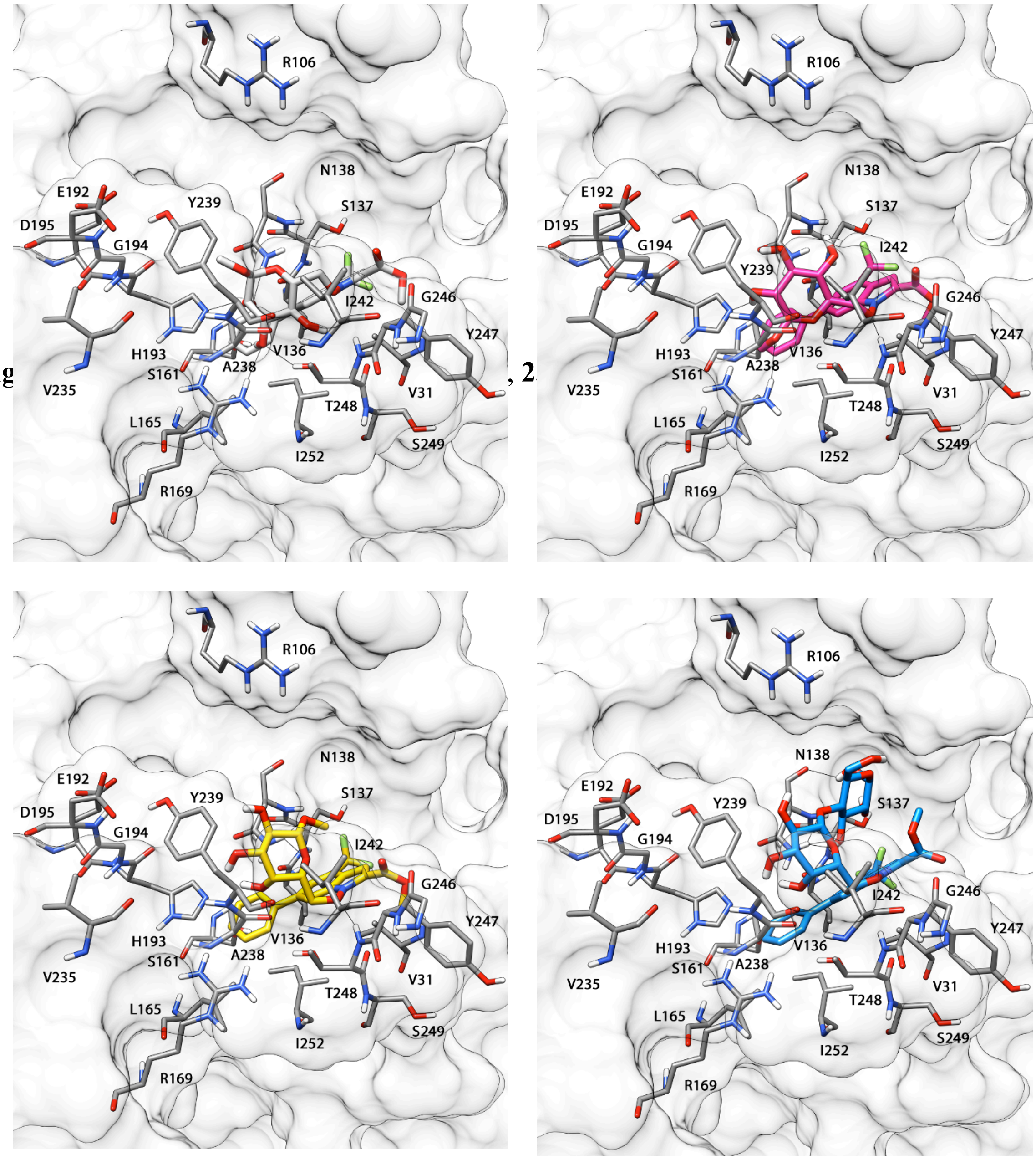

Figure S3. Binding disposition of **13**, **18**, **19** and **23** into *h*LDH5.
